# Hydroxylated Polychlorinated Biphenyls Are Emerging Legacy Pollutants in Contaminated Sediments

**DOI:** 10.1021/acs.est.1c04780

**Published:** 2022-02-02

**Authors:** Panithi Saktrakulkla, Xueshu Li, Andres Martinez, Hans-Joachim Lehmler, Keri C. Hornbuckle

**Affiliations:** † Interdisciplinary Graduate Program in Human Toxicology, The University of Iowa, Iowa City, Iowa 52242, United States; ‡ Department of Civil and Environmental Engineering, IIHR-Hydroscience and Engineering, The University of Iowa, Iowa City, Iowa 52242, United States; § Department of Occupational and Environmental Health, College of Public Health, The University of Iowa, Iowa City, Iowa 52242, United States

**Keywords:** OH-PCBs, sediment, Aroclors, New Bedford Harbor, Altavista, Indiana Harbor and Ship Canal

## Abstract

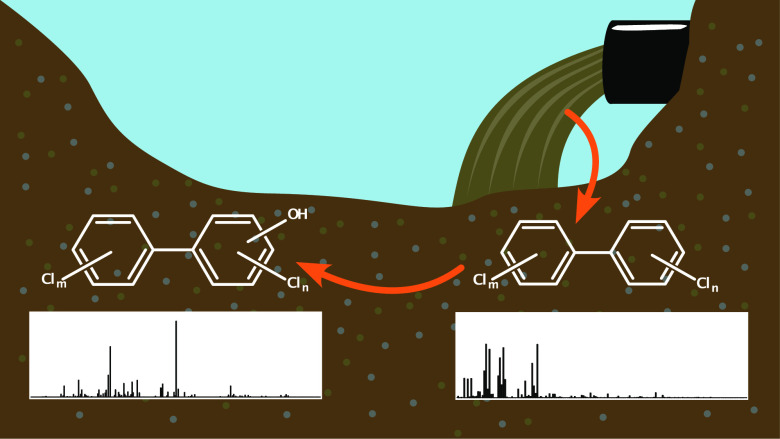

We measured the concentrations of 837 hydroxylated polychlorinated biphenyls (OH-PCBs, in 275 chromatographic peaks) and 209 polychlorinated biphenyls (PCBs, in 174 chromatographic peaks) in sediments from New Bedford Harbor in Massachusetts, Altavista wastewater lagoon in Virginia, and the Indiana Harbor and Ship Canal in Indiana, USA and in the original commercial PCB mixtures Aroclors 1016, 1242, 1248, and 1254. We used the correlation between homologues and the peak responses to quantify the full suite of OH-PCBs including those without authentic standards available. We found that OH-PCB levels are approximately 0.4% of the PCB levels in sediments and less than 0.0025% in Aroclors. The OH-PCB congener distributions of sediments are different from those of Aroclors and are different according to sites. We also identified a previously unknown compound, 4-OH-PCB52, which together with 4′-OH-PCB18 made up almost 30% of the OH-PCBs in New Bedford Harbor sediments but less than 1.2% in the Aroclors and 3.3% in any other sediments. This indicates site-specific environmental transformations of PCBs to OH-PCBs. We conclude that the majority of OH-PCBs in these sediments are generated in the environment. Our findings suggest that these toxic breakdown products of PCBs are prevalent in PCB-contaminated sediments and present an emerging concern for humans and ecosystems.

## Abstract 2

We measured the full suite of hydroxylated polychlorinated biphenyls (OH-PCBs) and found that OH-PCBs are prevalent in PCB-containing sediments with site-specific generation.

## Introduction

Mono-hydroxylated polychlorinated biphenyls (OH-PCBs) are major oxidative products of polychlorinated biphenyls (PCBs), a group of persistent organic pollutants.^1−7^ Although OH-PCBs have been reported in biota, air, and sediments,^8−13^ their prevalence and origin in the environment are not fully understood. They are analytically challenging to measure, and of 837 possible congeners,^14^ only a small fraction is commercially available as analytical standards. Like other unidentified or unknown pollutants, the lack of standards prevents the determination of quantitative assessment of risk to exposure.^15−17^

OH-PCBs may be produced in the environment from PCBs by biological metabolism, atmospheric reaction, and oxidation processes in water treatment.^1−4,8,18^ Biological metabolism of PCBs to OH-PCBs in mammals, plants, and aerobic bacteria is the most widely studied among the three. The metabolism is mediated through cytochrome P450 monooxygenase (CYP450) by direct electrophilic addition of oxygen or by the formation of a transient reactive arene oxide and the spontaneous rearrangement to OH-PCBs.^1−4,19,20^ OH-PCB metabolites formed in the living organisms can enter the food chain and be released into the environment.^2^ Abiotic formation of OH-PCBs has been demonstrated in the laboratory and in silico through the atmospheric reaction between volatile PCBs and hydroxyl radicals,^21−26^ both of which are present in the atmosphere.^23,27^ However, no study has directly observed the formation of OH-PCBs through this mechanism in the environment.^2^ OH-PCBs may be a result of advanced oxidation processes utilized in the treatment process because the concentrations of OH-PCBs in the surface waters collected near sewage treatment plants in urban areas were reported to be relatively higher than those collected from offshore from Lake Ontario.^8^

OH-PCBs have toxicity profiles similar to and distinct from PCBs. OH-PCBs are hormone disruptors.^1−7^ OH-PCBs disrupt estrogen homeostasis by strongly binding to estrogen receptors and acting as either receptor agonists or antagonists.^28−30^ An increase of OH-PCB levels shows a clear relationship with a decrease of thyroid hormone levels.^31−33^ Through the inhibition of sulfotransferases (SULTs), OH-PCBs can also elevate the levels of active estrogens and thyroid hormones and inhibit the sulfation of OH-PCBs.^34−37^ Although OH-PCBs themselves are not known to be carcinogens, they can be metabolized to ultimate carcinogen PCB quinones.^7,38,39^

Despite various toxicities, little is known about the sources and magnitude of OH-PCB levels in the environment. Sediment is one of the biggest environmental reservoirs of PCBs and is a potential source of OH-PCBs to the environment.^5−7,40^ However, we are aware of only three reports of OH-PCBs in sediments. Sakiyama et al. (2007) reported OH-PCBs in sediments from Osaka, Japan.^9^ Sun et al. (2016) detected OH-PCBs in sewage sludge in China.^12^ We also reported OH-PCBs in sediments from the Indiana Harbor and Ship Canal in Indiana, USA (IHSC), in Marek et al. (2013).^10^ To our knowledge, there are no studies of the full suite of OH-PCBs in sediments but only small sets of those with standards available. Furthermore, it remains unclear if these compounds are widely prevalent in PCB-contaminated sediments.

Here, we report for the first time the total OH-PCB concentrations and their congener-specific distributions in sediments and in Aroclors. We hypothesize that the OH-PCBs are present in PCB-contaminated sediments, and the concentrations and distributions are proportional to their PCB contamination level. To address this hypothesis, we analyzed OH-PCBs and PCBs in sediment samples collected from three different sites contaminated with PCBs: New Bedford Harbor in Massachusetts, USA (NBH); Altavista wastewater lagoon in Virginia, USA (AWL); and IHSC. We also consider two hypotheses of the origin of OH-PCBs in sediments: (1) they were present in the original commercial PCB mixtures or (2) they were/are generated later in the environment. Our study therefore includes the determination of OH-PCBs and PCBs in four Aroclors: Aroclor 1016 (A1016), Aroclor 1242 (A1242), Aroclor 1248 (A1248), and Aroclor 1254 (A1254), whose combination composed more than 99% of the Aroclors sold in the US,^5,6^ and these Aroclors are likely to be the original mixtures contaminating the three sites.^41−43^

## Materials and Methods

### PCB Standard Solution

Details of PCB standard solution can be found in Table S1. Briefly, the PCB standard solution was composed of (i) 209 PCB congeners (AccuStandard, New Haven, CT, USA); (ii) 10 ^13^C-PCB surrogate standards (mono- to deca-chlorinated; Wellington Laboratories, Guelph, ON, Canada); and (iii) d_5_-PCB30 (Cambridge Isotope Laboratories, Andover, MA, USA), which is used as an internal standard together with PCB204 (AccuStandard). PCB congener names are in accordance with the United States Environmental Protection Agency (US EPA).^44^

### MeO-PCB Standard Solution

Details of MeO-PCB standard solution can be found in Table S2. Briefly, the MeO-PCB standard solution was composed of (i) 70 mono-MeO-PCBs (9 mono-, 5 di-, 6 tri-, 12 tetra-, 13 penta-, 8 hexa-, 10 hepta-, 6 octa-, and 1 nona-chlorinated; AccuStandard and Wellington Laboratories); (ii) seven ^13^C_12_-mono-MeO-PCB surrogate standards (di- to hepta-chlorinated; Wellington); and (iii) two internal standards (d_5_-PCB30 and PCB204). Synthetic standards 4-MeO-PCB8 and 4-MeO-PCB52 were prepared through the Suzuki coupling of a corresponding benzene boronic acid and a methoxylated bromochlorobenzene as described elsewhere along with their NMR and X-ray diffraction data.^45−47^

### Sediment Samples

Twelve surficial sediment samples from three PCB-contaminated sites were used in this study (Table S3): five sediments collected from the upper harbor of NBH in December 2017 using a piston-core sampling device (predredging samples from the remediation area O);^48,49^ five sediments collected from AWL in September 2015 using hand auger;^43^ and two were collected from the IHSC in May 2009 by a submersible vibrocoring system.^42^ See Table S3 for the geographic coordinates. To protect OH-PCBs from decay and to prevent additional generation, the sediment samples were kept in an airtight container and refrigerated at 4 °C until extraction and analysis. We have previously reported concentrations of PCBs in sediments from these sites, but for this study, all samples were freshly extracted and analyzed as described here.

The PCB extraction method was modified from our previous methods.^41−43^ Sediment samples were weighed and mixed with an equal weight of diatomaceous earth (DE; Thermo Fisher Scientific, Waltham, MA, USA). One gram of 1:1 sediment/DE mixtures were spiked with 25 ng of each of the ^13^C_12_-PCB surrogate standards, before extracting with hexane:acetone (1:1 v/v) (pesticide grade; Fisher Chemical, Fair Lawn, NJ, USA) by pressurized liquid extraction (PLE; Dionex ASE 200, Sunnyvale, CA, USA) using conditions in concordance with EPA methods 8082A and 3545A.^50,51^ The extracts were washed with concentrated sulfuric acid (Fisher Chemical), passed through sulfuric acid/silica gel (1:2 w/w) columns (Flash Chromatography Grade; 70–230 Mesh; Fisher Chemical), and finally spiked with 25 ng of each of the d_5_-PCB30 and PCB204 internal standards.

For OH-PCB analysis, we modified our previous methods to improve the extraction efficiency and to reduce matrix interference.^10,52^ Twenty grams of the 1:1 sediment/DE mixtures were spiked with 25 ng of each of the ^13^C_12_-OH-PCB surrogate standards and then extracted twice with hexane/acetone (1:1 v/v) by PLE. Next, the extracts were washed with concentrated hydrochloric acid (Fisher Chemical), partitioned with 1 N potassium hydroxide/ethanol (1:1 v/v) (Fisher Chemical and Sigma-Aldrich, St. Louis, MO, USA, respectively) to remove PCBs, neutralized with hydrochloric acid, and derivatized to MeO-PCBs with diazomethane in diethyl ether.^53^ MeO-PCBs were then separated from residual lipids by gel permeation chromatography.^52^ Finally, the extracts were passed through hydrochloric acid/silica gel (1:3 w/w) columns and spiked with 25 ng of each of the d_5_-PCB30 and PCB204 internal standards.

### Aroclor Samples

Monsanto Aroclors including A1016, A1242, and A1254 in their original containers were provided by Dr. Larry Robertson through the Synthesis Core of Iowa Superfund Research Program (ISRP) (Figure S1). A1248 was purchased from AccuStandard. Aroclors were analyzed in triplicate. For PCB analysis, 4 μg of Aroclors were diluted with hexane and spiked with 25 ng of each of the surrogate and internal standards.

For OH-PCB analysis, 1 g of Aroclors were spiked with 25 ng of each of the ^13^C_12_-OH-PCB surrogate standards, partitioned with 1 N potassium hydroxide/ethanol (1:1 v/v) to remove PCBs, neutralized with hydrochloric acid, derivatized to MeO-PCBs with diazomethane, passed through hydrochloric acid/silica gel (1:3 w/w) columns, and spiked with 25 ng of each of the internal standards.

### Instruments and Quantifications

Gas chromatography (GC) coupled with mass spectrometry (MS) was employed for identification and quantification of PCBs and OH-PCBs using methods previously reported.^41,42,52,54^ PCBs were analyzed with an Agilent 7890A GC equipped a with Supelco SPB-Octyl capillary column (30 m, 0.25 mm i.d., 0.25 μm film thickness) coupled with an Agilent 7000B Triple Quadrupole (QqQ) MS. OH-PCBs derivatized to MeO-PCBs were analyzed with an Agilent 7890B GC equipped with a Supelco SPB-Octyl capillary column coupled with an Agilent 7000D QqQ MS. See Tables S4 and S5 for chromatographic conditions. Two hundred and eight PCBs (174 chromatographic peaks), 70 OH-PCBs derivatized to MeO-PCBs whose standards were commercially available (64 chromatographic peaks), and surrogate standards were quantified in positive electron ionization (EI) at 70 eV in multiple reaction monitoring (MRM) mode using the internal standard method.^52,54^

OH-PCBs whose standards were not commercially available were quantified with our novel strategy using the correlation between the peak responses and the number of chlorines in the molecules or homologues as described elsewhere.^52^ Briefly, unknown OH-PCBs derivatized to MeO-PCBs were first identified based on chlorine isotope distribution in selected ion monitoring (SIM) mode (Table S6). Then, they were verified by comparing their fragmentation patterns from collision-induced dissociation (CID) at 10–50 eV to those of MeO-PCB standards in product ion mode. Next, the peak responses of MeO-PCB standards were captured in sextuplicate in positive EI at 30 eV in SIM mode and were used to generate a model to predict the peak responses of unknown MeO-PCBs from homologues (Figures S2 and S3). Finally, the peak responses of unknown OH-PCBs as MeO-PCBs were captured under the same conditions and were compared to those from the predictive model with the corresponding homologue to calculate the compound mass in each sample. The full suite of PCB and OH-PCB concentrations and associated metadata has been released to a data repository.^55^

### Quality Assurance (QA) and Quality Control (QC)

The capability of our methods was assessed through the extraction efficiency and the limits of quantification (LOQs) using median (*x̃*) and arithmetic mean ± standard deviation (*x̅* ± *s*). Moreover, internal standard (d_5_-PCB30 and PCB204) and multiple injection methods (≥3 for standards and ≥2 for samples) were employed to ensure precise quantification. We previously reported an assessment of the accuracy of our method using a solution of MeO-PCBs provided by Synthesis Core of ISRP.^52^

The extraction efficiency was represented by the recoveries of surrogate standards: PCBs in sediments (*x̃* = 100%; *x̅* ± *s* = 105 ± 28%); OH-PCBs in sediments (*x̃* = 104%; *x̅* ± *s* = 115 ± 43%); PCBs in Aroclors (*x̃* = 99%; *x̅* ± *s* = 102 ± 11%); and OH-PCBs in Aroclors (*x̃* = 91%; *x̅* ± *s* = 90 ± 20%) (see Tables S7 and S8). We used the surrogate standard recoveries to correct the PCB and OH-PCB masses in samples and method blanks.

The LOQs were obtained from the analysis of method blanks, where DE and hexane were used as method blanks in sediment and Aroclor analysis, respectively (see Tables S9 and S10). For PCB and known OH-PCB analysis, LOQs were calculated using the upper end of the 95% confidence interval (*x̅* + *t*_α = 0.05_ × *s*). LOQs varied among the types of analysis: PCBs in sediments (*x̃* = 0.14 ng/g; *x̅* ± *s* = 1.3 ± 3.1 ng/g); known OH-PCBs in sediments (*x̃* = 0.03; ng/g; *x̅* ± *s* = 0.05 ± 0.10 ng/g); PCBs in Aroclors (*x̃* = 0.09 ng/sample; *x̅* ± *s* = 0.13 ± 0.16 ng/sample); and known OH-PCBs in Aroclors (*x̃* = 0.65 ng/sample; *x̅* ± *s* = 0.82 ± 0.79 ng/sample). For the LOQs of unknown OH-PCBs, we used the upper end of the 95% confidence interval of geometric mean (GM) LOQs of known OH-PCBs ( exp ( ln(LOQ)® + *t*_α = 0.05_ × *s*_ln(LOQ)_)). LOQs of unknown OH-PCBs in sediments are 0.15 ng/g, and those in Aroclors are 2.4 ng/sample. Congener concentration or mass below the LOQ was given a value of zero.

### Statistical Analysis

Statistical analyses were computed in the R statistical computing environment (version 4.0.5).^56^ Packages “boot” (version 1.3–27) and “beeswarm” (version 0.3.1)^57−59^ were respectively used to compute and plot the model to predict the peak responses of unknown MeO-PCBs from homologues. Nonparametric tests and log-transformation were mainly utilized because the OH-PCB and PCB levels did not normally distribute. A significance level of 0.05 is used throughout this report.

To examine the similarity of OH-PCB distributions in sediments from the PCB-contaminated sites and in Aroclors, we first constructed OH-PCB congener profiles of each sample by arranging the concentrations of all 275 OH-PCB peaks found in this study by homologues and chromatographic peak elution orders. Then, we calculated cosine similarity (cos θ)^41,42,60,61^ of all possible pairs of samples. The cos θ range is between 0 and 1. The closer to 1, the more similar between the OH-PCB congener profiles. Finally, we examined the similarity among samples using the median (*x̃*) and arithmetic mean ± standard deviation (*x̅* ± *s*) of cos θ.

This is the first time that cos θ is used with the full suite of OH-PCB congener profiles,^60,61^ so we validated the reproducibility of OH-PCB congener profiles and the sensitivity of cos θ by evaluating the profiles of Aroclors (Figures S17–S20). We found that when the OH-PCB congener profiles of triplicate analysis were compared, cos θ approached perfect similarity: A1016 (0.96 and 0.98); A1248 (both 0.99); and A1256 (0.91 and 0.96). This indicates the reproducibility of our OH-PCB congener profiles. When the OH-PCB profiles of different Aroclors were compared, cos θ values were much lower (*x̃* = 0.08; *x̅* ± *s* = 0.12 ± 0.07). This indicates the sensitivity to differentiate congener profiles.

In A1242, the unknown OH-PCB levels were all below the LOQ, fewer than five congeners of known mono- and tri-chlorinated OH-PCBs were detected, and the total OH-PCB concentrations were too low to construct reliable profiles (Figure S18). We then excluded A1242 from further analysis.

## Results and Discussion

### OH-PCBs in PCB-Contaminated Sediments

**Figure 1:**
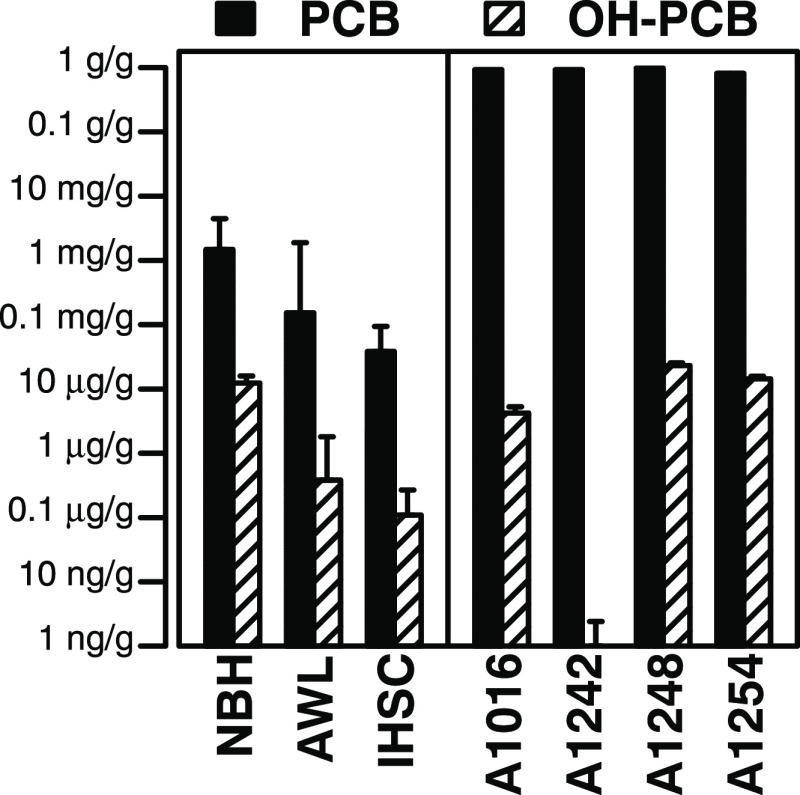
GMs of log-transformed total OH-PCB and PCB concentrations (μg/g dry weight) of NBH, AWL, and IHSC sediments and Aroclors. The error bars indicate geometric standard deviation.

The OH-PCB and PCB levels in sediments are highest in NBH followed by AWL and the IHSC (Figures 1 and S5–S16). In five NBH sediments, we found that the total OH-PCB concentrations range from 9.9 to 18 μg/g dry weight (DW) with a GM concentration of 12 μg/g DW, and the total PCB concentrations range from 240 to 3800 μg/g DW with a GM concentration of 1500 μg/g DW. NBH is an urban tidal estuary located in Massachusetts and is one of the largest PCB superfund sites in the United States.^62,63^ Aroclors were discharged into the estuary for more than 30 years before an attempt to reduce the PCB contamination by dredging started in 1994.^49,62−64^ The PCB levels we measured in sediments collected in 2017 are similar to those reported by the US EPA in 2005 and in 2012 but are higher than those in postdredging sediments in 2019.^49,63,64^

In five AWL sediments, we found that the total OH-PCB concentrations range from 0.039 to 2.6 μg/g DW with a GM concentration of 0.38 μg/g DW, and the total PCB concentrations range from 4.7 to 3800 μg/g DW with a GM concentration of 150 μg/g DW. AWL is a 25,000 m^2^ emergency wastewater overflow lagoon located in Altavista, Virginia.^65,66^ AWL was contaminated with PCBs from the industries in Altavista sometime before the manufacture of PCBs was halted in 1977.^65,66^ The PCB levels in AWL sediments in our study are comparable to those reported by Mattes et al. (2018).^43^ We found that the total OH-PCB concentrations in AWL sediments are significantly lower (Wilcoxon–Mann-Whitney test, *p*-value = 0.0079) and are about one third of those in NBH sediments. The PCB levels in AWL sediments are about one tenth of those in NBH sediments. However, the difference is not statistically significant (Wilcoxon-Mann–Whitney test, *p*-value = 0.22) because of the high variation and small sample sizes.

In two IHSC sediments, we found that the total OH-PCB concentrations are 0.058 and 0.21 μg/g DW, and the total PCB concentrations are 20 and 72 μg/g DW, respectively. The IHSC is in East Chicago, a heavily industrialized urban community on the southern shore of Lake Michigan.^67,68^ The IHSC is one of the largest tributary sources of PCBs into Lake Michigan.^41,42,67,68^ We have reported OH-PCB and PCB levels in IHSC sediments, in the air, and in human serum from East Chicago and have shown the importance of the IHSC as a source of airborne OH-PCBs and PCBs to the community.^10,13,41,42,69,70^ The total OH-PCB and PCB concentrations in IHSC sediments are a few percent of those in NBH sediments and about a 25% of those in AWL sediments. Comparing only the small subset of the known OH-PCB congeners and PCBs, the levels in this study are similar to our previous report.^10,41,42^ The sample size of IHSC sediments is too small to perform statistical analysis. We excluded another available IHSC sediment from the report because of a method error. See Table S11 for discussion.

### Relationships between OH-PCB and PCB Levels Are Different among the PCB-Contaminated Sites

**Figure 2:**
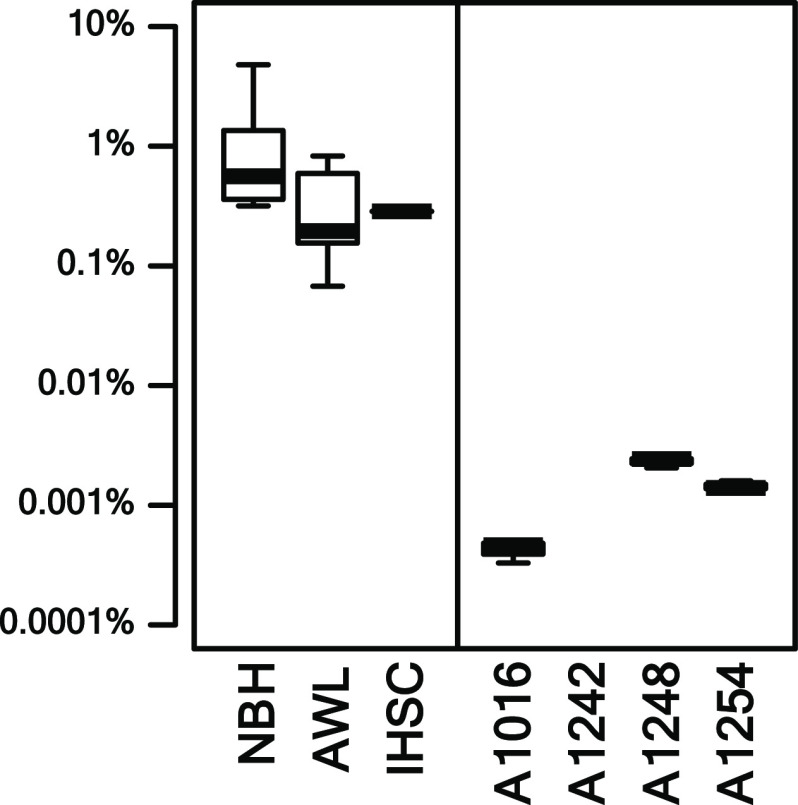
Boxplot of log-transformed relative concentrations of OH-PCBs to PCBs (log([OH-PCBs]/[PCBs])) of NBH, AWL, and IHSC sediments and those of Aroclors. The whiskers indicate 1.5 times the interquartile range.

The relative concentrations of OH-PCBs to PCBs ([OH-PCBs]/[PCBs]) in the sediments in this study are highly variable and range from 0.068 to 4.8% with a GM [OH-PCBs]/[PCBs] of 0.43%. In NBH sediments, the [OH-PCBs]/[PCBs] range from 0.32 to 4.8% with a GM [OH-PCBs]/[PCBs] of 0.84% (Figure 2). We found neither the linear (Pearson test, *p*-value = 0.87) nor the range correlations (Spearman test, *p*-value = 0.78) between OH-PCB and PCB levels in NBH sediments (Figure S4).

In AWL sediments, the [OH-PCBs]/[PCBs] range from 0.068 to 0.83% with a GM [OH-PCBs]/[PCBs] of 0.25%. We found a rank correlation between OH-PCB and PCB levels in AWL sediments (Spearman test, *p*-value = 0.017). Investigating further, we found a correlation between log-OH-PCB and log-PCB concentrations in AWL sediments (*p*-value = 0.002). The log–log correlation (β_1_ = 0.61) suggests that if the PCB concentration in an AWL sediment is 2 times higher than that in another sediment, its OH-PCB concentration is expected to be higher by 2^0.61^ = 1.5 times.

Overall, we cannot confirm our hypothesis that OH-PCB levels and distributions in sediments are proportional to the PCB contamination levels because the correlations between OH-PCB and PCB levels are not linear and are different between NBH and AWL. This difference may be due to the geographical characteristics and human activities at the two PCB-contaminated sites. At NBH, river tidal flow and numerous human activities, including dredging remediation, disturb the sediment.^62,63^ At AWL, there is no water flow. Only wind and occasional sampling activities provide any sediment disturbance.^65^ Moreover, OH-PCB and PCB levels in sediments also depend on microbial activities. OH-PCBs may be transformed to methoxylated polychlorinated biphenyls.^12^ PCBs can be aerobically oxidized with dioxygenase enzymes in the biphenyl upper pathway to catechol metabolites which can spontaneously be cleaved and transform to chlorobenzoate or can rearrange to orthoquinone metabolites.^71−73^ PCBs can also be anaerobically dechlorinated by becoming terminal electron acceptors in the respiration chains.^43,72,74^ The microbial communities in the brackish of NBH and in the freshwater of AWL are different and may alter OH-PCB and PCB levels differently. We could not evaluate the correlation between OH-PCB and PCB levels in IHSC because we have only two samples. However, both samples had the [OH-PCBs]/[PCBs] at 0.28% which is comparable to those in NBH and AWL sediments. Additional studies are needed to fully understand the relationship between OH-PCB and PCB levels.

### Sources of OH-PCBs in Sediment

**Figure 3:**
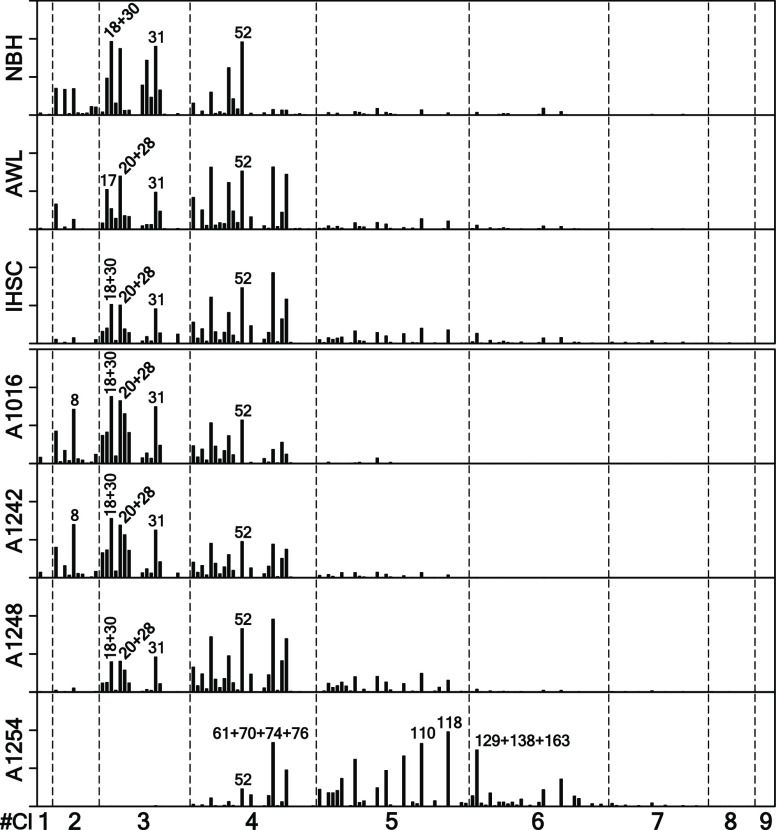
PCB congener profiles of NBH, AWL, and IHSC sediments and those of Aroclors. *Y*-axis is the concentration fraction of total OH-PCBs with each tick indicating 5%. On *X*-axis, PCB congeners are arranged by congener names.

We hypothesized that OH-PCBs in the original Aroclors are a direct source of OH-PCBs in the sediments. We previously reported the finding of OH-PCBs as a residual in original Aroclors using 65 OH-PCB commercial standards.^10^ Here, we report the total concentrations of the full suite of OH-PCBs in four selected Aroclors (Figure 1) together with their congener-specific concentrations (Figures S17–S20). We found total OH-PCB concentrations in A1016, A1248, and A1254 to be 4.2, 23, and 14 μg/g-Aroclor, respectively, while that in A1242 is much lower at 0.57 ng/g-Aroclor. Using cos θ, we found that the PCB congener distributions in our Aroclors (Figure 3) were consistent with those in the corresponding Aroclors reported by Frame et al. (1996) with more than 0.97 similarity.^75^ The total PCB concentration in the four Aroclors ranged from 81 to 98% w/w with an arithmetic mean of 91% w/w.

Our findings do not support our original hypothesis that OH-PCBs in Aroclor mixtures explain their presence in sediments. Differential sorption or accumulation also cannot explain the differences we found. Because of the hydroxyl moiety, OH-PCBs are more water-soluble than PCBs, especially those higher-chlorinated OH-PCBs with a high value of acid dissociation constant (Ka).^76,77^ Although the salinity of water can enhance the sedimental sorption of OH-PCBs,^78−80^ the amount of OH-PCBs in sediments should be less than those present in Aroclors if OH-PCBs originated solely from Aroclors. However, we found the contrary (Figure 2). The [OH-PCBs]/[PCBs] in the sediment from the three PCB-contaminated sites were significant and much greater than those found in the four Aroclors (Wilcoxon–Mann-Whitney test, *p*-value < 0.0001). The [OH-PCBs]/[PCBs] in sediments is at least 30 times higher than those in Aroclors with a GM difference of 4500 times. This evidence shows that the contribution of the original commercial mixtures to OH-PCB contamination in sediments is negligible.

**Figure 4:**
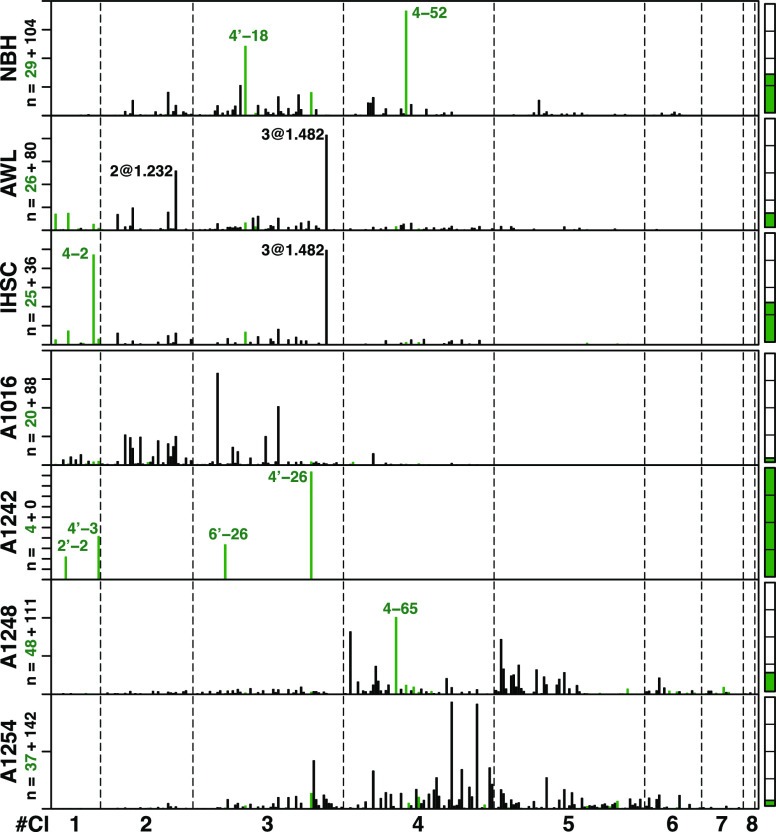
OH-PCB congener profiles of NBH, AWL, and IHSC sediments, and those of Aroclors. *Y*-axis is the concentration fraction of total OH-PCBs with each tick indicating 10%. On *X*-axis, OH-PCB congeners are arranged by chlorination and the peak elution order (Supelco SPB-Octyl capillary column). The green bars indicate OH-PCBs identified with authentic standards, and black bars indicate OH-PCBs known only by the homologue (#Cl) and RRT. Under each title on the left side are the numbers of known (green) and unknown (black) OH-PCBs. Bars on the right side show the proportions of known (green) to the total OH-PCB concentrations with each tick indicating 25%.

The OH-PCB congener distributions of sediments from the same PCB-contaminated site are similar, but they differ among the sites (Figure 4 and Table S12). Using cos θ, we found that the OH-PCB congener profiles of sediments from the same PCB-contaminated sites are similar with more than 0.74 of mean similarity. However, when the OH-PCB congener profiles are compared among the PCB-contaminated sites, they are different from the others with less than 0.23 of mean similarity. OH-PCB congener distributions in sediments are site-specific.

NBH is well known to be mainly contaminated with A1016 and A1242,^62,63^ and our NBH sediments show a PCB signal about 0.77 similar to the two Aroclors (Figure 3). However, we found that the OH-PCB congener distributions of NBH sediments are different from those of A1016 (Figure 4) with only about 0.20 similarity (Table S12). We also found that the majority of OH-PCBs in NBH sediments have 3 to 4 chlorines, but the OH-PCBs in A1016 have 2 to 3 chlorines (Table S13). Moreover, we found many OH-PCBs with 5 to 6 chlorines in NBH sediments, while these homologues are almost undetected in A1016. This evidence also supports our hypothesis that the environmental production is the origin of OH-PCBs in sediments.

The OH-PCB congener and homologue distributions of AWL and IHSC sediments are different from those of A1248 with less than 0.05 similarity, although they were previously reported to be contaminated with A1248 and have PCB signals similar to A1248 with about 0.90 similarity.^41−43^ While the majority of OH-PCB in AWL and IHSC sediments have 1 to 3 chlorines, those in A1248 have 3 to 5 chlorines (Figure 4 and Table S13). However, we found that the OH-PCB congener distributions of AWL and IHSC sediments are partly similar, 0.40. We consider two possibilities to explain this finding. First, a portion of the OH-PCBs in sediment may have originated from the OH-PCBs originally present in Aroclors. Second, a portion of the OH-PCBs in these sediments may be due to the environmental transformation of the common PCB congeners contaminating the sediments. In both scenarios, environmental conditions and the age of sediments due to the original contamination would affect the accumulation of OH-PCBs in the sediment. The microbial communities are likely to be different between the two sites, thus resulting in the differences in the specificities to PCB precursors and OH-PCB products and in conversion rates.^43,73^ Also, the difference in the pH, salinity, and flow of the water as well as the compositions of sediments would result in different accumulation of OH-PCBs in the two sediments. Accordingly, the OH-PCB signals of AWL and IHSC sediments can be partly similar but different from those in A1248. More studies are yet to understand the biotic and abiotic production, the fate and transport, and the accumulation of OH-PCBs in the environment.

### Predominant OH-PCB Congeners in Sediments

Although OH-PCBs can be quantified through our semitarget strategy, authentic standards are essential for biological studies. This requires structure elucidation and synthesis of individual OH-PCB congeners. While the synthesis of all 837 possible mono-OH-PCB congeners is impractical, identifying predominant OH-PCB congeners present in the environment will enable toxicological study and risk assessment. Of 275 individual or coeluting OH-PCBs congeners that we found in this study, 106 congeners were detected only in Aroclors, 35 congeners were present only in sediments, and 134 congeners were found in both sediments and Aroclors. These 134 congeners explain about 80–100% of the total OH-PCB concentrations in the sediments. We detected about 80 OH-PCB congeners in each sediment sample, and their concentrations are not linearly distributed. About 60 of them have concentrations less than 1/*n* of the total concentrations, where n is the number of congeners in the sample. The sums of the remaining 20 congeners explain about 80% of the total concentrations in the samples. The two predominant peaks with the highest concentrations explain more than 30% of the total concentrations in the sediments, and, thus, are most likely an environmental and human health concern.

We conducted further studies to identify the predominant OH-PCB congeners. We found that only about 30% of the total OH-PCBs we detected are known and have commercial standards available (Table S13). In all NBH sediments, we found an unknown tetra-chlorinated OH-PCB with a relative retention time when compared with the d5-PCB30 internal standard (RRT) of 1.524. Considering the similarity of CID fragmentation patterns to para-hydroxylated tetra-chlorinated OH-PCB standards^52^ and the high levels of PCB52 in NBH sediments (Figure 3), we speculated that this compound is 4-OH-PCB52 and confirmed with an authentic standard (Figures S21–S26).^46^ We also identified another predominant peak as 4′-OH-PCB18 using a commercial standard.

In AWL and IHSC sediments, we found two unknown OH-PCBs: one containing two chlorines with an RRT of 1.232 (2@1.232) and the other one containing three chlorines with an RRT of 1.482 (3@1.482). While 3@1.482 is found in both AWL and IHSC sediments, 2@1.232 is found only in AWL sediments. Considering (i) the high levels of PCB4, PCB8, PCB17, PCB18 + PCB30, PCB20 + 28, and PCB31 in AWL and/or IHSC sediments (Figures S11–S14), (ii) the PCB congener distribution of A1248,^75^ (iii) the possible oxidative metabolites of these PCB congeners,^1−4^ and (iv) the similarity of CID fragmentation patterns to para-OH-PCB standards,^52^ we speculate that 2@1.232 is 4-OH-PCB4 or 4-OH-PCB8, and 3@1.482 is 4-OH-PCB18 or 4-OH-PCB31. We have confirmed that 2@1.232 is not 4-OH-PCB8 with an authentic standard.^45,47^ The other OH-PCB standards are yet to be synthesized. We also identified another predominant peak as 4-OH-PCB2 in IHSC sediments using a commercial standard.

We found that the concentrations of 4-OH-PCB52 and 4′-OH-PCB18 in NBH sediments are the first and the second highest among the 275 OH-PCB individual or coeluting congeners found in this study, and they account for about 18 and 12% of the total OH-PCB concentrations in NBH sediments, respectively. Neither 4-OH-PCB52 nor 4′-OH-PCB18 exceeds 1.2% in any Aroclors and 3.3% in any other sediments, although PCB52 and PCB18 are predominant in most samples (Figure 3). While the concentrations of 3@1.482 are the highest in both AWL and IHSC sediments, 2@1.232 and 4-OH-PCB2 are respectively the second. Their combinations compose more than 33 and 48% of the total concentrations in AWL and IHSC sediments, respectively. Neither of these congeners exceeds 0.9% in any Aroclors except 5.0% of 4-OH-PCB2 in A1016, whose PCB signal differs from those of AWL and IHSC sediments. 3@1.482 and 2@1.232 are less than 1.8% in NBH sediments. We conclude that these predominant OH-PCB congeners in sediments were/are produced in the environment, site-specifically.

**Figure 5:**
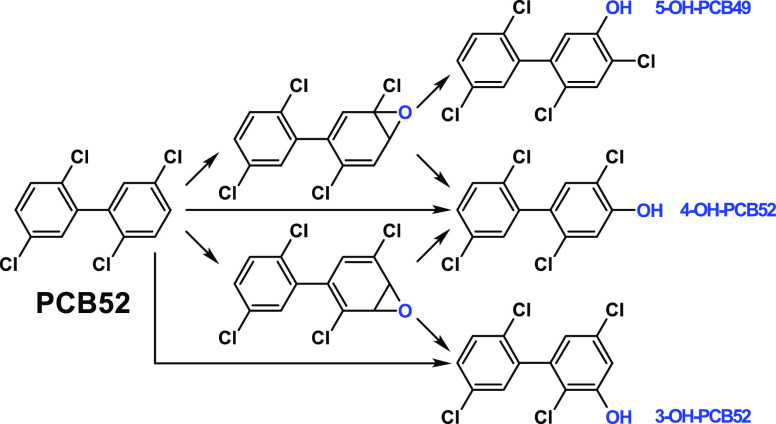
General hydroxylation through CYP450 of PCB52. The scheme is modified from the study by Grimm et al. (2015).^3^

PCB52, PCB18, PCB3, and PCB2 can be metabolized by CYP450 enzymes to several OH-PCBs (Figures 5 and S27–S29).^1−3,19,20,81^ Para-OH-PCBs, especially those with vicinal nonchlorine-substituted positions, are the more preferable products than meta- or ortho-OH-PCBs.^82^ Para-OH-PCBs are also the major OH-PCB congeners detected in human serum.^69,70,83−85^ Likewise, the abiotic oxidation of PCB52, PCB18, PCB3, and PCB2 is more likely to produce para OH-PCBs.^1−3,8,19,20,81^ In addition, PCB52 can sequentially be dechlorinated to PCB18 and be oxidized to OH-PCB18 by microorganisms.^43,72,74^ We found the high levels of 4-OH-PCB52 and 4′-OH-PCB18 only in NBH sediments, although PCB52 and PCB18 are prominent in all PCB-contaminated sites. This indicates site-specific environmental production of 4-OH-PCB52 and 4′-OH-PCB18. We also found that the 4-OH-PCB2 level in IHSC sediments is about 3.5 times larger than those of PCB3 and PCB2 combined and is more than 13 times greater than the combination of the more abiotically preferable products 4′-OH-PCB3 and 4′-OH-PCB2. This indicates that most of PCB3 and PCB2 may enzymatically and specifically be metabolized to 4-OH-PCB2. Although the actual sources of these predominant OH-PCB congeners still need further studies, our findings suggest the environmental production of potentially toxic OH-PCBs in sediments.

4-OH-PCB52 and 4′-OH-PCB18 are more toxic than their parent PCB congeners. 4-OH-PCB52 is the most toxic congener against the viability of neural cell lines N27 and SH-SY5Y and hepatic cell line HepG2 among the commonly observed airborne PCBs (PCB3, PCB8, PCB11, and PCB52) and their OH-PCB and PCB sulfate derivatives.^86^ 4-OH-PCB52 also has higher potency than PCB52 toward the ryanodine receptor, an important calcium channel for neurodevelopment and synaptic plasticity.^87^ Likewise, 4′-OH-PCB18 and 4-OH-PCB2 have agonistic activities via estrogen receptors and antagonistic activities via the androgen receptor and glucocorticoid receptor.^88^ The estrogenic effect of 4′-OH-PCB18 is stronger than that of PCB18.^47^ 4-OH-PCB2 can affect neurodevelopment through neuronal elongation in a dose-dependent manner.^89,90^

Although there are a number of studies reporting OH-PCBs in environmental matrices and in humans,^8−13,69,70,83−85^ there are only two exposure and toxicokinetic studies linking OH-PCBs in environmental matrices and in humans.^91,92^ Generally OH-PCBs are less bioavailable than PCBs. Considering the molecular weights and Ka, lower-chlorinated OH-PCBs are more likely to be in neutral forms, volatilize into the air, and be absorbed through inhalation, while higher-chlorinated OH-PCBs are more likely to be in ionized forms, dissolve in water, and be absorbed through ingestion.^76,77^ Although the OH-PCB levels in sediments are a small fraction of the PCB levels, the mass may still pose significant additional risk in many PCB contamination sites across the country. OH-PCBs can also be converted by plants, microorganisms, and mammals to MeO-PCBs that are more volatile.^12,92,93^ OH-PCBs are more vulnerable to phase II metabolism, be excreted relatively faster, and less likely to accumulate in the bodies than PCBs. Still, they can be retained in the bodies for several days.^91,92^ OH-PCBs bind strongly to serum proteins although reversibly and are found in several organs.^1−4^ Nevertheless, the numbers of OH-PCB exposures and toxicokinetic studies are still limited, and more studies are needed to fully understand their risk of toxicity in humans.

## Supporting Information Available

Sediment sampling locations, GC/MS method, QA/QC results, OH-PCB and PCB congener profiles, cos θ analysis, metabolic schemes of some PCBs, and the CID fragmentation pattern of 4-OH-PCB52 (PDF)

## References

[ref1] Letcher R. J.; Klasson-Wehler E.; Bergman A. Methyl Sulfone and Hydroxylated Metabolites of Polychlorinated Biphenyls. In Volume 3 Anthropogenic Compounds Part K; Hutzinger O., Paasivirta J., Eds.; Springer Berlin Heidelberg: Berlin, Heidelberg, 2000; pp 315–359.

[ref2] Tehrani R.; Van Aken B. Hydroxylated polychlorinated biphenyls in the environment: sources, fate, and toxicities. Environ. Sci. Pollut. Res. 2014, 21, 6334–6345. 10.1007/s11356-013-1742-6. PMC3812322 23636595

[ref3] Grimm F. A.; Hu D.; Kania-Korwel I.; Lehmler H. J.; Ludewig G.; Hornbuckle K. C.; Duffel M. W.; Bergman A.; Robertson L. W. Metabolism and metabolites of polychlorinated biphenyls (PCBs). Crit. Rev. Toxicol. 2015, 45, 245–272. 10.3109/10408444.2014.999365. 25629923 PMC4383295

[ref4] Dhakal K.; Gadupudi G. S.; Lehmler H. J.; Ludewig G.; Duffel M. W.; Robertson L. W. Sources and toxicities of phenolic polychlorinated biphenyls (OH-PCBs). Environ. Sci. Pollut. Res. Int. 2018, 25, 16277–16290. 10.1007/s11356-017-9694-x. 28744683 PMC5785587

[ref5] Agency for Toxic Substances and Disease Registry (ATSDR). Toxicological Profile for Polychlorinated Biphenyls (PCBs); U.S. Department of Health & Human Services: Atlanta, GA, USA, 2000. URL: https://www.atsdr.cdc.gov/toxprofiles/tp17.pdf. 36888731

[ref6] Agency for Toxic Substances and Disease Registry (ATSDR). Addendum to the Toxicological Profile for Polychlorinated Biphenyls (PCBs); U.S. Department of Health & Human Services: Atlanta, GA, USA, 2011. URL: https://www.atsdr.cdc.gov/toxprofiles/pcbs_addendum.pdf. 36888731

[ref7] International Agency for Research on Cancer (IARC). Polychlorinated Biphenyls and Polybrominated Biphenyls; World Health Organization (WHO): Lyon, France, 2016; Vol. 107. ISBN: 978-92-832-1218-8. URL: https://publications.iarc.fr/Book-And-Report-Series/Iarc-Monographs-On-The-Identification-Of-Carcinogenic-Hazards-To-Humans/Polychlorinated-Biphenyls-And-Polybrominated-Biphenyls-1978.

[ref8] Ueno D.; Darling C.; Alaee M.; Campbell L.; Pacepavicius G.; Teixeira C.; Muir D. Detection of Hydroxylated Polychlorinated Biphenyls (OH-PCBs) in the Abiotic Environment: Surface Water and Precipitation from Ontario, Canada. Environ. Sci. Technol. 2007, 41, 1841–1848. 10.1021/es061539l. 17410773

[ref9] Sakiyama T.; Yamamoto A.; Kakutani N.; Fukuyama J.; Okumura T. Hydroxylated polychlorinated biphenyls (OH-PCBs) in the aquatic environment: levels and congener profiles in sediments from Osaka, Japan. Organohalogen Compd. 2007, 69, 1380–1383.

[ref10] Marek R. F.; Martinez A.; Hornbuckle K. C. Discovery of Hydroxylated Polychlorinated Biphenyls (OH-PCBs) in Sediment from a Lake Michigan Waterway and Original Commercial Aroclors. Environ. Sci. Technol. 2013, 47, 8204 10.1021/es402323c. 23862721 PMC3781593

[ref11] Awad A. M.; Martinez A.; Marek R. F.; Hornbuckle K. C. Occurrence and Distribution of Two Hydroxylated Polychlorinated Biphenyl Congeners in Chicago Air. Environ. Sci. Technol. Lett. 2016, 3, 47–51. 10.1021/acs.estlett.5b00337. 30246046 PMC6148743

[ref12] Sun J.; Zhu L.; Pan L.; Wei Z.; Song Y.; Zhang Y.; Qu L.; Zhan Y. Detection of methoxylated and hydroxylated polychlorinated biphenyls in sewage sludge in China with evidence for their microbial transformation. Sci. Rep. 2016, 6, 29782 10.1038/srep29782. 27417462 PMC4945941

[ref13] Marek R. F.; Thorne P. S.; Herkert N. J.; Awad A. M.; Hornbuckle K. C. Airborne PCBs and OH-PCBs Inside and Outside Urban and Rural U.S. Schools. Environ. Sci. Technol. 2017, 51, 7853–7860. 10.1021/acs.est.7b01910. 28656752 PMC5777175

[ref14] Buser H. R.; Zook D. R.; Rappe C. Determination of methyl sulfone-substituted polychlorobiphenyls by mass spectrometric techniques with application to environmental samples. Anal. Chem. 1992, 64, 1176–1183. 10.1021/ac00034a018.

[ref15] U.S. Environmental Protection Agency (EPA). Guidelines for Human Exposure Assessment; Washington, DC, USA, 2019. URL: https://www.epa.gov/sites/production/files/2020-01/documents/guidelines_for_human_exposure_assessment_final2019.pdf.

[ref16] International Programme on Chemical Safety (IPCS). Human Health Risk Assessment Toolkit: Chemical Hazards; World Health Organization (WHO): Geneva, Switzerland, 2010. URL: https://apps.who.int/iris/handle/10665/44458.

[ref17] Schymanski E. L.; Jeon J.; Gulde R.; Fenner K.; Ruff M.; Singer H. P.; Hollender J. Identifying Small Molecules via High Resolution Mass Spectrometry: Communicating Confidence. Environ. Sci. Technol. 2014, 48, 2097–2098. 10.1021/es5002105. 24476540

[ref18] Sedlak D. L.; Andren A. W. Aqueous-phase oxidation of polychlorinated biphenyls by hydroxyl radicals. Environ. Sci. Technol. 1991, 25, 1419–1427. 10.1021/es00020a009.

[ref19] Furukawa K.; Fujihara H. Microbial degradation of polychlorinated biphenyls: biochemical and molecular features. J. Biosci. Bioeng. 2008, 105, 433–449. 10.1263/jbb.105.433. 18558332

[ref20] Aken B. V.; Correa P. A.; Schnoor J. L. Phytoremediation of polychlorinated biphenyls: new trends and promises. Environ. Sci. Technol. 2010, 44, 2767–2776. 10.1021/es902514d. 20384372 PMC3025541

[ref21] Anderson P. N.; Hites R. A. OH Radical Reactions: The Major Removal Pathway for Polychlorinated Biphenyls from the Atmosphere. Environ. Sci. Technol. 1996, 30, 1756–1763. 10.1021/es950765k.

[ref22] Brubaker W. W.; Hites R. A. Gas-Phase Oxidation Products of Biphenyl and Polychlorinated Biphenyls. Environ. Sci. Technol. 1998, 32, 3913–3918. 10.1021/es9805021.

[ref23] Sinkkonen S.; Paasivirta J. Degradation half-life times of PCDDs, PCDFs and PCBs for environmental fate modeling. Chemosphere 2000, 40, 943–949. 10.1016/s0045-6535(99)00337-9. 10739030

[ref24] Totten L. A.; Eisenreich S. J.; Brunciak P. A. Evidence for destruction of PCBs by the OH radical in urban atmospheres. Chemosphere 2002, 47, 735–746. 10.1016/s0045-6535(01)00326-5. 12079069

[ref25] Mandalakis M.; Berresheim H.; Stephanou E. G. Direct Evidence for Destruction of Polychlorobiphenyls by OH Radicals in the Subtropical Troposphere. Environ. Sci. Technol. 2003, 37, 542–547. 10.1021/es020163i. 12630470

[ref26] Liao Z.; Zeng M.; Wang L. Atmospheric oxidation mechansim of polychlorinated biphenyls (PCBs) initiated by OH radicals. Chemosphere 2020, 240, 124756 10.1016/j.chemosphere.2019.124756. 31563106

[ref27] Hornbuckle K. C.; Eisenreich S. J. Dynamics of gaseous semivolatile organic compounds in a terrestrial ecosystem—effects of diurnal and seasonal climate variations. Atmos. Environ. 1996, 30, 3935–3945. 10.1016/1352-2310(96)00135-5.

[ref28] Connor K.; Ramamoorthy K.; Moore M.; Mustain M.; Chen I.; Safe S.; Zacharewski T.; Gillesby B.; Joyeux A.; Balaguer P. Hydroxylated polychlorinated biphenyls (PCBs) as estrogens and antiestrogens: structure-activity relationships. Toxicol. Appl. Pharmacol. 1997, 145, 111–123. 10.1006/taap.1997.8169. 9221830

[ref29] Machala M.; Bláha L.; Lehmler H. J.; Plísková M.; Májková Z.; Kapplová P.; Sovadinová I.; Vondrácek J.; Malmberg T.; Robertson L. W. Toxicity of hydroxylated and quinoid PCB metabolites: inhibition of gap junctional intercellular communication and activation of aryl hydrocarbon and estrogen receptors in hepatic and mammary cells. Chem. Res. Toxicol. 2004, 17, 340–347. 10.1021/tx030034v. 15025504

[ref30] DeCastro B. R.; Korrick S. A.; Spengler J. D.; Soto A. M. Estrogenic activity of polychlorinated biphenyls present in human tissue and the environment. Environ. Sci. Technol. 2006, 40, 2819–2825. 10.1021/es051667u. 16683629

[ref31] Kato Y.; Ikushiro S.; Haraguchi K.; Yamazaki T.; Ito Y.; Suzuki H.; Kimura R.; Yamada S.; Inoue T.; Degawa M. A possible mechanism for decrease in serum thyroxine level by polychlorinated biphenyls in Wistar and Gunn rats. Toxicol. Sci. 2004, 81, 309–315. 10.1093/toxsci/kfh225. 15254343

[ref32] Otake T.; Yoshinaga J.; Enomoto T.; Matsuda M.; Wakimoto T.; Ikegami M.; Suzuki E.; Naruse H.; Yamanaka T.; Shibuya N.; Yasumizu T.; Kato N. Thyroid hormone status of newborns in relation to in utero exposure to PCBs and hydroxylated PCB metabolites. Environ. Res. 2007, 105, 240–246. 10.1016/j.envres.2007.03.010. 17490634

[ref33] Dallaire R.; Muckle G.; Dewailly E.; Jacobson S. W.; Jacobson J. L.; Sandanger T. M.; Sandau C. D.; Ayotte P. Thyroid hormone levels of pregnant inuit women and their infants exposed to environmental contaminants. Environ. Health Perspect. 2009, 117, 1014–1020. 10.1289/ehp.0800219. 19590699 PMC2702396

[ref34] Liu Y.; Smart J. T.; Song Y.; Lehmler H. J.; Robertson L. W.; Duffel M. W. Structure-activity relationships for hydroxylated polychlorinated biphenyls as substrates and inhibitors of rat sulfotransferases and modification of these relationships by changes in thiol status. Drug Metab. Dispos. 2009, 37, 1065–1072. 10.1124/dmd.108.026021. 19196841 PMC2677757

[ref35] Ekuase E. J.; Liu Y.; Lehmler H. J.; Robertson L. W.; Duffel M. W. Structure-activity relationships for hydroxylated polychlorinated biphenyls as inhibitors of the sulfation of dehydroepiandrosterone catalyzed by human hydroxysteroid sulfotransferase SULT2A1. Chem. Res. Toxicol. 2011, 24, 1720–1728. 10.1021/tx200260h. 21913674 PMC3196794

[ref36] Dhakal K.; Uwimana E.; Adamcakova-Dodd A.; Thorne P. S.; Lehmler H. J.; Robertson L. W. Disposition of phenolic and sulfated metabolites after inhalation exposure to 4-chlorobiphenyl (PCB3) in female rats. Chem. Res. Toxicol. 2014, 27, 1411–1420. 10.1021/tx500150h. 24988477 PMC4137987

[ref37] Parker V. S.; Squirewell E. J.; Lehmler H. J.; Robertson L. W.; Duffel M. W. Hydroxylated and sulfated metabolites of commonly occurring airborne polychlorinated biphenyls inhibit human steroid sulfotransferases SULT1E1 and SULT2A1. Environ. Toxicol. Pharmacol. 2018, 58, 196–201. 10.1016/j.etap.2018.01.010. 29408762 PMC6078096

[ref38] Ludewig G.; Lehmann L.; Esch H.; Robertson L. W. Metabolic Activation of PCBs to Carcinogens in Vivo – A Review. Environ. Toxicol. Pharmacol. 2008, 25, 241–246. 10.1016/j.etap.2007.10.029. 18452002 PMC2364599

[ref39] Robertson L. W.; Ludewig G. Polychlorinated Biphenyl (PCB) carcinogenicity with special emphasis on airborne PCBs. Gefahrst. Reinhalt. Luft = Air Quality Control 2011, 71, 25–32. 21686028 PMC3113507

[ref40] Tanabe S. PCB problems in the future: Foresight from current knowledge. Environ. Pollut. 1988, 50, 5–28. 10.1016/0269-7491(88)90183-2. 15092651

[ref41] Martinez A.; Norström K.; Wang K.; Hornbuckle K. C. Polychlorinated biphenyls in the surficial sediment of Indiana Harbor and Ship Canal Lake Michigan. Environ. Int. 2010, 36, 849–854. 10.1016/j.envint.2009.01.015. 19268364 PMC2888873

[ref42] Martinez A.; Hornbuckle K. C. Record of PCB congeners, sorbents and potential toxicity in core samples in Indiana Harbor and Ship Canal. Chemosphere 2011, 85, 542–547. 10.1016/j.chemosphere.2011.08.018. 21899876 PMC3222236

[ref43] Mattes T. E.; Ewald J. M.; Liang Y.; Martinez A.; Awad A.; Richards P.; Hornbuckle K. C.; Schnoor J. L. PCB dechlorination hotspots and reductive dehalogenase genes in sediments from a contaminated wastewater lagoon. Environ. Sci. Pollut. Res. Int. 2018, 25, 16376–16388. 10.1007/s11356-017-9872-x. 28803405 PMC6206866

[ref44] United States Environmental Protection Agency (US EPA). Table of PCB Species by Congener Number. https://www.epa.gov/sites/production/files/2015-09/documents/congenertable.pdf (accessed April 12, 2021).

[ref45] Li X.; Parkin S.; Duffel M. W.; Robertson L. W.; Lehmler H.-J. An efficient approach to sulfate metabolites of polychlorinated biphenyls. Environ. Int. 2010, 36, 843–848. 10.1016/j.envint.2009.02.005. 19345419 PMC2939219

[ref46] Rodriguez E. A.; Li X.; Lehmler H. J.; Robertson L. W.; Duffel M. W. Sulfation of Lower Chlorinated Polychlorinated Biphenyls Increases Their Affinity for the Major Drug-Binding Sites of Human Serum Albumin. Environ. Sci. Technol. 2016, 50, 5320–5327. 10.1021/acs.est.6b00484. 27116425 PMC4883002

[ref47] Pěnčíková K.; Svržková L.; Strapáčová S.; Neča J.; Bartoňková I.; Dvořák Z.; Hýžd’alová M.; Pivnička J.; Pálková L.; Lehmler H. J.; Li X.; Vondráček J.; Machala M. In vitro profiling of toxic effects of prominent environmental lower-chlorinated PCB congeners linked with endocrine disruption and tumor promotion. Environ. Pollut. 2018, 237, 473–486. 10.1016/j.envpol.2018.02.067. 29518658 PMC5908724

[ref48] Battelle Memorial Institute. Draft Final: Sediment Monitoring Summary Report 2014 Remedial Dredging Season. United States Environmental Protection Agency (US EPA). https://www.epa.gov/sites/production/files/2015-09/documents/580304.pdf (accessed April 12, 2021).

[ref49] Patrick Curran. New Bedford Harbor Superfund Site: Final Dredge Areas I/N and O Hybrid Dredge Data Report. United States Environmental Protection Agency (US EPA). https://semspub.epa.gov/src/document/01/100012483.pdf (accessed April 12, 2021).

[ref50] United States Environmental Protection Agency (US EPA). Method 3545A: Pressurized Fluid Extraction (PFE). https://www.epa.gov/sites/default/files/2015-12/documents/3545a.pdf (accessed November 24, 2021).

[ref51] United States Environmental Protection Agency (US EPA). Method 8082A: Polychlorinated Biphenyls (PCBs) by Gas Chromatography. https://www.epa.gov/sites/production/files/2015-12/documents/8082a.pdf (accessed November 24, 2021).

[ref52] Saktrakulkla P.; Dhakal R. C.; Lehmler H. J.; Hornbuckle K. C. A semi-target analytical method for quantification of OH-PCBs in environmental samples. Environ. Sci. Pollut. Res. Int. 2019, 27, 8859 10.1007/s11356-019-05775-x. 31359319 PMC6986979

[ref53] Kania-Korwel I.; Zhao H.; Norstrom K.; Li X.; Hornbuckle K. C.; Lehmler H.-J. Simultaneous extraction and clean-up of polychlorinated biphenyls and their metabolites from small tissue samples using pressurized liquid extraction. J. Chromatogr. A 2008, 1214, 37–46. 10.1016/j.chroma.2008.10.089. 19019378 PMC2648864

[ref54] Saktrakulkla P.; Lan T.; Hua J.; Marek R. F.; Thorne P. S.; Hornbuckle K. C. Polychlorinated Biphenyls in Food. Environ. Sci. Technol. 2020, 54, 11443–11452. 10.1021/acs.est.0c03632. 32816464 PMC7759298

[ref55] Saktrakulkla P.; Martinez A.; Lehmler H.-J.; Hornbuckle K. C. Dataset for OH-PCBs are emerging legacy pollutants in contaminated sediments; University of Iowa, 2021. 10.1021/acs.est.1c04780 PMC8851693 35107261

[ref56] R Core Team. R: A language and environment for statistical computing. 2021, (Version 4.0.5). https://www.R-project.org/ (accessed March 31, 2021).

[ref57] Davison A. C.; Hinkley D. V. Bootstrap Methods and their Application; Cambridge University Press: Cambridge, 1997.

[ref58] Canty, A.; Ripley, B. D. boot: Bootstrap R (S-Plus) Functions. 2021, (Version 1.3–27). https://CRAN.R-project.org/package=boot (accessed February 12, 2021-02-12).

[ref59] Eklund, A. beeswarm: The Bee Swarm Plot, an Alternative to Stripchart. 2021, (Version 0.3.1). https://CRAN.R-project.org/package=beeswarm (accessed March 07, 2021).

[ref60] Jöreskog K. G.; Klovan J. E.; Reyment R. A. Geological factor analysis. Elsevier: Netherlands, 1976; p 178. ISBN: 9780444413673.

[ref61] Davis J. C. Statistics and data analysis in geology, 2nd ed.; Wiley: New York, 1986; p 646. ISBN: 9780471080794.

[ref62] United States Environmental Protection Agency (US EPA). New Bedford Harbor Cleanup Plans, Technical Documents and Environmental Data. https://www.epa.gov/new-bedford-harbor/new-bedford-harbor-cleanup-plans-technical-documents-and-environmental-data (accessed April 19, 2021).

[ref63] Nelson W. G.; Bergen B. J. The New Bedford Harbor Superfund site long-term monitoring program (1993–2009). Environ. Monit. Assess. 2012, 184, 7531–7550. 10.1007/s10661-012-2517-0. 22367364

[ref64] Bergen B. J.; Nelson W. G.; Mackay J.; Dickerson D.; Jayaraman S. Environmental Monitoring Of Remedial Dredging At The New Bedford Harbor, Ma, Superfund Site. Environ. Monit. Assess. 2005, 111, 257–275. 10.1007/s10661-005-8223-4. 16311831

[ref65] Koerting, K. Small trees are soaking up PCBs in Altavista’s old wastewater pond. The news and advance. https://newsadvance.com/news/local/small-trees-are-soaking-up-pcbs-in-altavistas-old-wastewater-pond/article_f8e9a240-aa55-11e3-a8c6-0017a43b2370.html (accessed April 21, 2021).

[ref66] Walter A. Altavista assesses PCB removal plans; The news and advance. https://newsadvance.com/news/local/altavista-assesses-pcb-removal-plans/article_d91a20c4-16e8-11e5-a3e8-031f0fa793bb.html (accessed April 19, 2021).

[ref67] United States Environmental Protection Agency (US EPA). Results of the Lake Michigan Mass Balance Study: Biphenyls and trans-Nonachlor Data Report. https://www.epa.gov/sites/production/files/2015-08/documents/lmmbpcb.pdf (accessed April 21, 2021).

[ref68] United States Environmental Protection Agency (US EPA). Results of the Lake Michigan Mass Balance Project: Polychlorinated Biphenyls Modeling Report. https://www.epa.gov/sites/production/files/2015-08/documents/lmmbp-pcb-report.pdf (accessed April 21, 2021).

[ref69] Marek R. F.; Thorne P. S.; Wang K.; DeWall J.; Hornbuckle K. C. PCBs and OH-PCBs in Serum from Children and Mothers in Urban and Rural U.S. Communities. Environ. Sci. Technol. 2013, 47, 3353–3361. 10.1021/es304455k. 23452180 PMC3645264

[ref70] Marek R. F.; Thorne P. S.; DeWall J.; Hornbuckle K. C. Variability in PCB and OH-PCB serum levels in children and their mothers in urban and rural U.S. communities. Environ. Sci. Technol. 2014, 48, 13459–13467. 10.1021/es502490w. 25300024 PMC4238695

[ref71] Furukawa K.; Suenaga H.; Goto M. Biphenyl dioxygenases: functional versatilities and directed evolution. J. Bacteriol. 2004, 186, 5189–5196. 10.1128/JB.186.16.5189-5196.2004. 15292119 PMC490896

[ref72] Pieper D. H.; Seeger M. Bacterial Metabolism of Polychlorinated Biphenyls. Microb. Physiol. 2008, 15, 121–138. 10.1159/000121325. 18685266

[ref73] Bako C. M.; Mattes T. E.; Marek R. F.; Hornbuckle K. C.; Schnoor J. L. Biodegradation of PCB congeners by Paraburkholderia xenovorans LB400 in presence and absence of sediment during lab bioreactor experiments. Environ. Pollut. 2021, 271, 116364 10.1016/j.envpol.2020.116364. 33412450 PMC8183161

[ref74] Wiegel J.; Wu Q. Microbial reductive dehalogenation of polychlorinated biphenyls. FEMS Microbiol. Ecol. 2000, 32, 1–15. 10.1111/j.1574-6941.2000.tb00693.x. 10779614

[ref75] Frame G. M.; Cochran J. W.; Bøwadt S. S. Complete PCB congener distributions for 17 aroclor mixtures determined by 3 HRGC systems optimized for comprehensive, quantitative, congener-specific analysis. J. High Resolut. Chromatogr. 1996, 19, 657 10.1002/jhrc.1240191202.

[ref76] Rayne S.; Forest K. pK (a) values of the monohydroxylated polychlorinated biphenyls (OH-PCBs), polybrominated biphenyls (OH-PBBs), polychlorinated diphenyl ethers (OH-PCDEs), and polybrominated diphenyl ethers (OH-PBDEs). J. Environ. Sci. Health, Part A: Toxic/Hazard. Subst. Environ. Eng. 2010, 45, 1322–1346. 10.1080/10934529.2010.500885. 20658412

[ref77] Yu H.; Wondrousch D.; Yuan Q.; Lin H.; Chen J.; Hong H.; Schürmann G. Modeling and predicting pKa values of mono-hydroxylated polychlorinated biphenyls (HO-PCBs) and polybrominated diphenyl ethers (HO-PBDEs) by local molecular descriptors. Chemosphere 2015, 138, 829–836. 10.1016/j.chemosphere.2015.08.012. 26295542

[ref78] Means J. C. Influence of salinity upon sediment-water partitioning of aromatic hydrocarbons. Mar. Chem. 1995, 51, 3–16. 10.1016/0304-4203(95)00043-Q.

[ref79] Endo S.; Pfennigsdorff A.; Goss K.-U. Salting-Out Effect in Aqueous NaCl Solutions: Trends with Size and Polarity of Solute Molecules. Environ. Sci. Technol. 2012, 46, 1496–1503. 10.1021/es203183z. 22191628

[ref80] Oh S.; Shin W. S.; Kim H. T. Effects of pH, dissolved organic matter, and salinity on ibuprofen sorption on sediment. Environ. Sci. Pollut. Res. 2016, 23, 22882–22889. 10.1007/s11356-016-7503-6. PMC5101273 27572692

[ref81] Zhang C.-Y.; Flor S.; Ruiz P.; Ludewig G.; Lehmler H.-J. Characterization of the Metabolic Pathways of 4-Chlorobiphenyl (PCB3) in Hep G2 Cells Using the Metabolite Profiles of Its Hydroxylated Metabolites. Environ. Sci. Technol. 2021, 55, 9052–9062. 10.1021/acs.est.1c01076. 34125531 PMC8264946

[ref82] Mills R. A.; Millis C. D.; Dannan G. A.; Guengerich F. P.; Aust S. D. Studies on the structure-activity relationships for the metabolism of polybrominated biphenyls by rat liver microsomes. Toxicol. Appl. Pharmacol. 1985, 78, 96–104. 10.1016/0041-008x(85)90309-6. 2994255

[ref83] Park J. S.; Petreas M.; Cohn B. A.; Cirillo P. M.; Factor-Litvak P. Hydroxylated PCB metabolites (OH-PCBs) in archived serum from 1950-60s California mothers: a pilot study. Environ. Int. 2009, 35, 937–942. 10.1016/j.envint.2009.04.002. 19439357 PMC2699597

[ref84] Eguchi A.; Nomiyama K.; Ochiai M.; Mizukawa H.; Nagano Y.; Nakagawa K.; Tanaka K.; Miyagawa H.; Tanabe S. Simultaneous detection of multiple hydroxylated polychlorinated biphenyls from a complex tissue matrix using gas chromatography/isotope dilution mass spectrometry. Talanta 2014, 118, 253–261. 10.1016/j.talanta.2013.10.031. 24274296

[ref85] Ma S.; Ren G.; Zeng X.; Yu Z.; Sheng G.; Fu J. Polychlorinated biphenyls and their hydroxylated metabolites in the serum of e-waste dismantling workers from eastern China. Environ. Geochem. Health 2018, 40, 1931–1940. 10.1007/s10653-017-9958-x. 28477162

[ref86] Rodriguez E. A.; Vanle B. C.; Doorn J. A.; Lehmler H. J.; Robertson L. W.; Duffel M. W. Hydroxylated and sulfated metabolites of commonly observed airborne polychlorinated biphenyls display selective uptake and toxicity in N27, SH-SY5Y, and Hep G2 cells. Environ. Toxicol. Pharmacol. 2018, 62, 69–78. 10.1016/j.etap.2018.06.010. 29986280 PMC6092199

[ref87] Sethi S.; Morgan R. K.; Feng W.; Lin Y.; Li X.; Luna C.; Koch M.; Bansal R.; Duffel M. W.; Puschner B.; Zoeller R. T.; Lehmler H. J.; Pessah I. N.; Lein P. J. Comparative Analyses of the 12 Most Abundant PCB Congeners Detected in Human Maternal Serum for Activity at the Thyroid Hormone Receptor and Ryanodine Receptor. Environ. Sci. Technol. 2019, 53, 3948–3958. 10.1021/acs.est.9b00535. 30821444 PMC6457253

[ref88] Takeuchi S.; Shiraishi F.; Kitamura S.; Kuroki H.; Jin K.; Kojima H. Characterization of steroid hormone receptor activities in 100 hydroxylated polychlorinated biphenyls, including congeners identified in humans. Toxicology 2011, 289, 112–121. 10.1016/j.tox.2011.08.001. 21843587

[ref89] Mizukami-Murata S.; Fujita K.; Nakano T. Effect of lower chlorinated hydroxylated-polychlorobiphenyls on development of PC12 cells. Environ. Sci. Pollut. Res. 2018, 25, 16434–16445. 10.1007/s11356-017-9604-2. 28695493

[ref90] Mizukami-Murata S.; Sakakibara F.; Fujita K.; Fukuda M.; Kuramata M.; Takagi K. Detoxification of hydroxylated polychlorobiphenyls by Sphingomonas sp. strain N-9 isolated from forest soil. Chemosphere 2016, 165, 173–182. 10.1016/j.chemosphere.2016.08.127. 27649311

[ref91] Malmberg T.; Hoogstraate J.; Bergman Å.; Wehler E. K. Pharmacokinetics of two major hydroxylated polychlorinated biphenyl metabolites with specific retention in rat blood. Xenobiotica 2004, 34, 581–589. 10.1080/00498250410001713078. 15277017

[ref92] Wang M.-Y.; Zhang L.-F.; Wu D.; Cai Y.-Q.; Huang D.-M.; Tian L.-L.; Fang C.-L.; Shi Y.-F. Simulation experiment on OH-PCB being ingested through daily diet: Accumulation, transformation and distribution of hydroxylated-2, 2′, 4, 5, 5′-pentachlorobiphenyl (OH-PCB101) in mice. Sci. Total Environ. 2022, 802, 149891 10.1016/j.scitotenv.2021.149891. 34474296

[ref93] Li Y.; Bako C. M.; Saktrakulkla P.; Lehmler H.-J.; Hornbuckle K. C.; Schnoor J. L. Interconversion between methoxylated, hydroxylated and sulfated metabolites of PCB 3 in whole poplar plants. Sci. Total Environ. 2021, 785, 147341 10.1016/j.scitotenv.2021.147341. 33933776 PMC8610232

