# Correction to Hydroxylated Polychlorinated Biphenyls Are Emerging Legacy Pollutants in Contaminated Sediments

**DOI:** 10.1021/acs.est.2c01379

**Published:** 2022-04-25

**Authors:** Keri Hornbuckle, Panithi Saktrakulkla, Xueshu Li, Andres Martinez, Hans-Joachim Lehmler

Our recently published *ES&T* paper (https://doi.org/10.1021/acs.est.1c04780) has an extensive data set associated with it. Unfortunately, due to a production error, the citation to reference 55 omitted the DOI of the database. The corrected reference is as follows:

Saktrakulkla, P.; Li, X.; Martinez, A.; Lehmler, H.-J.; Hornbuckle, K. C. *Data set for hydroxylated polychlorinated biphenyls are emerging legacy pollutants in contaminated sediments*; University of Iowa, 2021. https://doi.org/10.25820/data.006146

In addition, the original article on the ACS publications website has been updated to show the correct reference 55.

