# Supplementary material for: Hydroxylated Polychlorinated Biphenyls Are Emerging Legacy Pollutants in Contaminated Sediments
Source: Environ Sci Technol. 2022 Feb 2;56(4):2269–78. doi: 10.1021/acs.est.1c04780 (PMC8851693; doi:10.1021/acs.est.1c04780)
Supplement: Supplementary file 1 — es1c04780_si_001.pdf [file es1c04780_si_001.pdf]

SUPPORTING INFORMATION

FOR

Hydroxylated polychlorinated biphenyls are emerging legacy pollutants in contaminated sediments

Panithi Saktrakulkla,[A,B] Xueshu Li,[C] Andres Martinez,[B]

Hans-Joachim Lehmler [A,C], and Keri C. Hornbuckle [A,B,*]

[A]Interdisciplinary Graduate Program in Human Toxicology,

[B]Department of Civil and Environmental Engineering, IIHR-Hydroscience and Engineering, and

[C]Department of Occupational and Environmental Health, College of Public Health,

The University of Iowa, Iowa City, IA, 52242, USA

Corresponding author

*Phone: (319) 384-0789; e-mail: keri-hornbuckle@uiowa.edu

Table of Contents

Table S1. PCB Standards. 5

Table S2. MeO-PCB standards used to detect and quantify OH-PCBs in samples. 6

Table S3. Sediment sample locations. 8

Table S4. GC injection temperature program. 9

Table S5. GC elution temperature program. 9

Table S6. The isotopic molecular masses of MeO-PCBs and (their distributions) used to identify OH-PCBs. 10

Table S7. Surrogate standard recoveries of method blanks. 11

Table S8. Surrogate standards recoveries of samples. 12

Table S9. LOQs of PCBs in sediments and Aroclors. 13

Table S10. LOQs of known OH-PCBs in sediments and Aroclors. 16

Table S11. The recoveries of OH-PCB surrogate standards in IHSC-66-72" sediment. 17

Table S12. The cosine similarity (cos θ) of OH-PCB congener profiles between pairs of samples. 18

Table S13. The number of OH-PCB congener peaks found in sediments from NBH, AWL, and IHSC, and in Aroclors, by or chlorination or homolog. 19

Figure S1. Aroclors 1016, 1242, 1248, and 1254 used in this study. 20

Figure S2. The procedure to generate a model to predict the relative response factors (RRFs) of unknown MeO-PCBs from the number of chlorines in the molecule or homolog groups (#Cl). 21

Figure S3. An example of exponential regression to predict the relative response factors (RRFs) of unknown MeO-PCBs from the number of chlorines in the molecule or homolog groups (#Cl). 22

Figure S4. Correlations between OH-PCB (Y-axis) and PCB (X-axis) levels in sediments in normal scale (left panels) and in log-log- scale (right panels). 23

Figure S5. The congener profiles of PCB (above) and OH-PCBs (below) in O-287-A sediment from New Bedford Harbor (NBH) in µg/g dry weight. 24

Figure S6. The congener profiles of PCB (above) and OH-PCBs (below) in O-288-A sediment from New Bedford Harbor (NBH) in µg/g dry weight. 25

Figure S7. The congener profiles of PCB (above) and OH-PCBs (below) in O-288-B sediment from New Bedford Harbor (NBH) in µg/g dry weight. 26

Figure S8. The congener profiles of PCB (above) and OH-PCBs (below) in O-331-A sediment from New Bedford Harbor (NBH) in µg/g dry weight. 27

Figure S9. The congener profiles of PCB (above) and OH-PCBs (below) in O-331-C sediment from New Bedford Harbor (NBH) in µg/g dry weight. 28

Figure S10. The congener profiles of PCB (above) and OH-PCBs (below) in C3 sediment from Altavista Waster Lagoon (AWL) in μg/g dry weight. 29

Figure S11. The congener profiles of PCB (above) and OH-PCBs (below) in D1 sediment from Altavista Waster Lagoon (AWL) in μg/g dry weight. 30

Figure S12. The congener profiles of PCB (above) and OH-PCBs (below) in D2 sediment from Altavista Waster Lagoon (AWL) in μg/g dry weight. 31

Figure S13. The congener profiles of PCB (above) and OH-PCBs (below) in E2 sediment from Altavista Waster Lagoon (AWL) in μg/g dry weight. 32

Figure S14. The congener profiles of PCB (above) and OH-PCBs (below) in F4 sediment from Altavista Waster Lagoon (AWL) in μg/g dry weight. 33

Figure S15. The congener profiles of PCB (above) and OH-PCBs (below) in 12-18" sediment from Indiana Harbor and Ship Canal (IHSC) in μg/g dry weight. 34

Figure S16. The congener profiles of PCB (above) and OH-PCBs (below) in 54-60" sediment from Indiana Harbor and Ship Canal (IHSC) in μg/g dry weight. 35

Figure S17. The congener profiles of PCB (above) and OH-PCBs (below) in Aroclor 1016 in μg/g dry weight. 36

Figure S18. The congener profiles of PCB (above) and OH-PCBs (below) in Aroclor 1242 in μg/g dry weight. 37

Figure S19. The congener profiles of PCB (above) and OH-PCBs (below) in Aroclor 1248 in μg/g dry weight. 38

Figure S20. The congener profiles of PCB (above) and OH-PCBs (below) in Aroclor 1254 in μg/g dry weight. 39

Figure S21. Chromatographic evidence of three major OH-PCB congeners found in sediments. The OH-PCB congeners derivatized to MeO-PCBs were captured with the QqQ mass spectrometer in positive EI in MRM mode. 40

Figure S22. The product ion mass spectrum captured with CID energy of 10 eV from the 321.9-m/z parent ion of synthetic 4-MeO-PCB52 standard (above) and that of 4@1.524 in O-288-A sediments from NBH (below). 41

Figure S23. The product ion mass spectrum captured with CID energy of 20 eV from the 321.9-m/z parent ion of synthetic 4-MeO-PCB52 standard (above) and that of 4@1.524 in O-288-A sediments from NBH (below). 42

Figure S24. The product ion mass spectrum captured with CID energy of 30 eV from the 321.9-m/z parent ion of synthetic 4-MeO-PCB52 standard (above) and that of 4@1.524 in O-288-A sediments from NBH (below). 43

Figure S25. The product ion mass spectrum captured with CID energy of 40 eV from the 321.9-m/z parent ion of synthetic 4-MeO-PCB52 standard (above) and that of 4@1.524 in O-288-A sediments from NBH (below). 44

Figure S26. The product ion mass spectrum captured with CID energy of 40 eV from the 321.9-m/z parent ion of synthetic 4-MeO-PCB52 standard (above) and that of 4@1.524 in O-288-A sediments from NBH (below). 45

Figure S27. General hydroxylation through CYP450 of PCB18 (bold lines) and its potential dechlorination parent PCBs (dash lines).. 46

Figure S28. General hydroxylation through CYP450 of PCB3 (bold lines) and its potential dechlorination parent PCBs (dash lines).. 47

Figure S29. General hydroxylation through CYP450 of PCB2 (bold lines) and its potential dechlorination parent PCBs (dash lines).. 48

Table S1. PCB Standards.

#Cl	IUPAC Name	Congener Name	Source*
1	Monochlorobiphenyls	PCB1 – PCB3	AS
2	Dichlorobiphenyls	PCB4 – PCB15	AS
3	Trichlorobiphenyls	PCB16 – PCB39	AS
4	Tetrachlorobiphenyls	PCB40 – PCB81	AS
5	Pentachlorobiphenyls	PCB82 – PCB127	AS
6	Hexachlorobiphenyls	PCB128 – PCB169	AS
7	Heptachlorobiphenyls	PCB170 – PCB193	AS
8	Octachlorobiphenyls	PCB194 – PCB203, and PCB205	AS
9	Nonachlorobiphenyls	PCB206 – PCB208	AS
10	Decachlorobiphenyls	PCB209	AS
1	4-Monochloro($^{13}C_{12}$)biphenyls	$^{13}C_{12}$-PCB3	WL
2	4,4'-Dichloro($^{13}C_{12}$)biphenyls	$^{13}C_{12}$-PCB15	WL
3	2,4',5-Trichloro($^{13}C_{12}$)biphenyls	$^{13}C_{12}$-PCB31	WL
4	2,2',5,5'-Tetrachloro($^{13}C_{12}$)biphenyls	$^{13}C_{12}$-PCB52	WL
5	2,3',4,4',5-Pentachloro($^{13}C_{12}$)biphenyls	$^{13}C_{12}$-PCB118	WL
6	2,2',4,4',5,5'-Hexachloro($^{13}C_{12}$)biphenyls	$^{13}C_{12}$-PCB153	WL
7	2,2',3,4,4',5,5'-Heptachloro($^{13}C_{12}$)biphenyls	$^{13}C_{12}$-PCB180	WL
8	2,2',3,3',4,4',5,5'-Octachloro($^{13}C_{12}$)biphenyls	$^{13}C_{12}$-PCB194	WL
9	2,2',3,3',4,4',5,5',6-Nonachloro($^{13}C_{12}$)biphenyls	$^{13}C_{12}$-PCB206	WL
10	2,2',3,3',4,4',5,5',6,6'-Decachloro($^{13}C_{12}$)biphenyls	$^{13}C_{12}$-PCB209	WL
3	2,4,6-Trichloro(2',3',4',5',6'-$^{2}H_{5}$)biphenyl	d_5-PCB30	CIL
8	2,2',3,4,4',5,6,6'-Octachlorobiphenyl	PCB204	AS

*Source: AS is AccuStandard (New Haven, CT, USA); WL is Wellington Laboratories (Guelph, ON, Canada); and CIL is Cambridge Isotope Laboratories (Andover, MA, USA).
PCB congener names are in accordance with the United States Environmental Protection Agency (US EPA): Table of PCB Species by Congener Number.[1]

Table S2. MeO-PCB standards used to detect and quantify OH-PCBs in samples.

#Cl	IUPAC Name	Congener Name	Source*
1	4-Methoxyl-2-Chlorobiphenyl	4-MeO-PCB1	AS
1	2-Methoxyl-3-Chlorobiphenyl	2-MeO-PCB2	AS
1	4-Methoxyl-3-Chlorobiphenyl	4-MeO-PCB2	AS
1	5-Methoxyl-3-Chlorobiphenyl	5-MeO-PCB2	AS
1	6-Methoxyl-3-Chlorobiphenyl	6-MeO-PCB2	AS
1	2'-Methoxyl-3-Chlorobiphenyl	2'-MeO-PCB2	AS
1	3'-Methoxyl-3-Chlorobiphenyl	3'-MeO-PCB2	AS
1	4'-Methoxyl-3-Chlorobiphenyl	4'-MeO-PCB2	AS
1	4'-Methoxyl-4-Chlorobiphenyl	4'-MeO-PCB3	AS
2	2'-Methoxyl-2,3-Dichlorobiphenyl	2'-MeO-PCB5	AS
2	3'-Methoxyl-2,5-Dichlorobiphenyl	3'-MeO-PCB9	AS
2	4'-Methoxyl-2,5-Dichlorobiphenyl	4'-MeO-PCB9	AS
2	2'-Methoxyl-3,4-Dichlorobiphenyl	2'-MeO-PCB12	AS
2	4-Methoxyl-3,5-Dichlorobiphenyl	4-MeO-PCB14	AS
3	4'-Methoxyl-2,2',5-Trichlorobiphenyl	4'-MeO-PCB18	AS
3	4'-Methoxyl-2,3',5-Trichlorobiphenyl	4'-MeO-PCB26	AS
3	6'-Methoxyl-2,3',5-Trichlorobiphenyl	6'-MeO-PCB26	AS
3	2'-Methoxyl-2,4,6-Trichlorobiphenyl	2'-MeO-PCB30	AS
3	3'-Methoxyl-2,4,6-Trichlorobiphenyl	3'-MeO-PCB30	AS
3	4'-Methoxyl-2,4,6-Trichlorobiphenyl	4'-MeO-PCB30	AS
4	3-Methoxyl-2,2',6,6'-Tetrachlorobiphenyl	3-MeO-PCB54	AS
4	2'-Methoxyl-2,3,4,5-Tetrachlorobiphenyl	2'-MeO-PCB61	AS
4	3'-Methoxyl-2,3,4,5-Tetrachlorobiphenyl	3'-MeO-PCB61	AS
4	4'-Methoxyl-2,3,4,5-Tetrachlorobiphenyl	4'-MeO-PCB61	WL
4	4-Methoxyl-2,3,5,6-Tetrachlorobiphenyl	4-MeO-PCB65	AS
4	2'-Methoxyl-2,3,5,6-Tetrachlorobiphenyl	2'-MeO-PCB65	AS
4	3'-Methoxyl-2,3,5,6-Tetrachlorobiphenyl	3'-MeO-PCB65	AS
4	4'-Methoxyl-2,3,5,6-Tetrachlorobiphenyl	4'-MeO-PCB65	AS
4	4'-Methoxyl-2,3',4,6-Tetrachlorobiphenyl	4'-MeO-PCB69	AS
4	6'-Methoxyl-2,3',4,6-Tetrachlorobiphenyl	6'-MeO-PCB69	AS
4	4'-Methoxyl-2,3',5,5'-Tetrachlorobiphenyl	4'-MeO-PCB72	AS
4	4'-Methoxyl-3,3',4,5'-Tetrachlorobiphenyl	4'-MeO-PCB79	WL
5	6'-Methoxyl-2,2',3,3',5-Pentachlorobiphenyl	6'-MeO-PCB83	AS
5	4'-Methoxyl-2,2',3,4,5-Pentachlorobiphenyl	4'-MeO-PCB86	AS
5	4'-Methoxyl-2,2',3,5,6-Pentachlorobiphenyl	4'-MeO-PCB93	AS
5	4-Methoxyl-2,2',3,4',5'-Pentachlorobiphenyl	4-MeO-PCB97	WL
5	4'-Methoxyl-2,2',4,5,5'-Pentachlorobiphenyl	4'-MeO-PCB101	WL
5	6'-Methoxyl-2,2',4,5,5'-Pentachlorobiphenyl	6'-MeO-PCB101	AS
5	2'-Methoxyl-2,3,3',4,5-Pentachlorobiphenyl	2'-MeO-PCB106	AS
5	4-Methoxyl-2,3,3',4',5-Pentachlorobiphenyl	4-MeO-PCB107	WL
5	4'-Methoxyl-2,3,3',4,5'-Pentachlorobiphenyl	4'-MeO-PCB108	WL
5	2'-Methoxyl-2,3,4,4',5-Pentachlorobiphenyl	2'-MeO-PCB114	WL
5	3-Methoxyl-2,3',4,4',5-Pentachlorobiphenyl	3-MeO-PCB118	WL
5	4'-Methoxyl-2,3',4,5,5'-Pentachlorobiphenyl	4'-MeO-PCB120	WL
5	4'-Methoxyl-3,3',4,5,5'-Pentachlorobiphenyl	4'-MeO-PCB127	WL

Continued on next page.

Table S2 (Continued). MeO-PCB standard.

#Cl	IUPAC Name	Congener Name	Source*
6	4'-Methoxyl-2,2',3,3',4,5'-Hexachlorobiphenyl	4'-MeO-PCB130	WL
6	4-Methoxyl-2,2',3,3',5,6-Hexachlorobiphenyl	4-MeO-PCB134	WL
6	5-Methoxyl-2,2',3,4,4',5'-Hexachlorobiphenyl	5-MeO-PCB138	AS
6	3'-Methoxyl-2,2',3,4,4',5'-Hexachlorobiphenyl	3'-MeO-PCB138	WL
6	4-Methoxyl-2,2',3,4',5,5'-Hexachlorobiphenyl	4-MeO-PCB146	WL
6	4'-Methoxyl-2,3,3',4,5,5'-Hexachlorobiphenyl	4'-MeO-PCB159	WL
6	4-Methoxyl-2,3,3',4',5,5'-Hexachlorobiphenyl	4-MeO-PCB162	WL
6	4-Methoxyl-2,3,3',4',5,6-Hexachlorobiphenyl	4-MeO-PCB163	WL
7	4'-Methoxyl-2,2',3,3',4,5,5'-Heptachlorobiphenyl	4'-MeO-PCB172	WL
7	4'-Methoxyl-2,2',3,3',4,5',6'-Heptachlorobiphenyl	4'-MeO-PCB177	WL
7	4-Methoxyl-2,2',3,3',5,5',6-Heptachlorobiphenyl	4-MeO-PCB178	WL
7	3'-Methoxyl-2,2',3,4,4',5,5'-Heptachlorobiphenyl	3'-MeO-PCB180	WL
7	3'-Methoxyl-2,2',3,4,4',5,6'-Heptachlorobiphenyl	3'-MeO-PCB182	WL
7	5-Methoxyl-2,2',3,4,4',5',6-Heptachlorobiphenyl	5-MeO-PCB183	AS
7	3'-Methoxyl-2,2',3,4,4',5',6-Heptachlorobiphenyl	3'-MeO-PCB183	WL
7	3'-Methoxyl-2,2',3,4,4',6,6'-Heptachlorobiphenyl	3'-MeO-CB184	WL
7	4-Methoxyl-2,2',3,4',5,5',6-Heptachlorobiphenyl	4-MeO-PCB187	WL
7	4-Methoxyl-2,3,3',4',5,5',6-Heptachlorobiphenyl	4-MeO-PCB193	WL
8	4'-Methoxyl-2,2',3,3',4,5,5',6-Octachlorobiphenyl	4'-MeO-PCB198	WL
8	4'-Methoxyl-2,2',3,3',4,5,5',6'-Octachlorobiphenyl	4'-MeO-PCB199	WL
8	4'-Methoxyl-2,2',3,3',4,5,6,6'-Octachlorobiphenyl	4'-MeO-PCB200	WL
8	4'-Methoxyl-2,2',3,3',4,5',6,6'-Octachlorobiphenyl	4'-MeO-PCB201	WL
8	4-Methoxyl-2,2',3,3',5,5',6,6'-Octachlorobiphenyl	4-MeO-PCB202	WL
8	3'-Methoxyl-2,2',3,4,4',5,5',6-Octachlorobiphenyl	3'-MeO-PCB203	WL
9	4'-Methoxyl-2,2',3,3',4,5,5',6,6'-Nonachlorobiphenyl	4'-MeO-PCB208	WL
2	4'-Methoxyl-3,4-Dichloro($^{13}C_{12}$)biphenyl	$^{13}C_{12}$-4'-MeO-PCB12	WL
3	4'-Methoxyl-2,4,5-Trichloro($^{13}C_{12}$)biphenyl	$^{13}C_{12}$-4'-MeO-PCB29	WL
4	4'-Methoxyl-2,3,4,5-Tetrachloro($^{13}C_{12}$)biphenyl	$^{13}C_{12}$-4'-MeO-PCB61	WL
5	4'-Methoxyl-2,3',4,5,5'-Pentachloro($^{13}C_{12}$)biphenyl	$^{13}C_{12}$-4'-MeO-PCB120	WL
6	4'-Methoxyl-2,3,3',4,5,5'-Hexachloro ($^{13}C_{12}$)biphenyl	$^{13}C_{12}$-4'-MeO-PCB159	WL
7	4'-Methoxyl-2,2',3,3',4,5,5'-Heptachloro($^{13}C_{12}$)biphenyl	$^{13}C_{12}$-4'-MeO-PCB172	WL
7	4-Methoxyl-2,2',3,4',5,5',6-Heptachloro ($^{13}C_{12}$)biphenyl	$^{13}C_{12}$-4-MeO-PCB187	WL
3	2,4,6-Trichloro(2',3',4',5',6'-$^{2}H_5$)biphenyl	d_5-PCB30	CIL
8	2,2',3,4,4',5,6,6'-Octachlorobiphenyl	PCB204	AS

*Source: AS is AccuStandard (New Haven, CT, USA); WL is Wellington Laboratories (Guelph, ON, Canada); and CIL is Cambridge Isotope Laboratories (Andover, MA, USA).
PCB congener names are in accordance with the US EPA: Table of PCB Species by Congener Number.[1]

Table S3. Sediment sample locations.

Site	Name	Geographic Coordinate	Depth	Date/Method	Reference
The Upper Harbor of New Bedford Harbor (pre-dredging samples from the remediation area O),[2] Massachusetts, USA	O-287-A	41.6727, -70.9167*	0 – 0.5 ft or 0 – 15 cm	2017 December 19/ a piston-core sampling device with 3-inch diameter Lexan™ core barrels*	Battelle Memorial Institute (2015)[3] and Patrick Curran (2019)[2]
	O-288-A	41.6727, -70.9167*	0 – 0.5 ft or 0 – 15 cm		
	O-288-B	41.6727, -70.9167*	0.5 – 1 ft or 15 – 30 cm		
	O-331-A	41.6725, -70.9170*	0 – 0.5 ft or 0 – 15 cm		
	O-331-C	41.6725, -70.9170*	0.8 – 1.3 ft or 24 – 40 cm		
Altavista Wastewater Lagoon, Virginia, USA	AWL-C3	37.113772, -79.272674	Surficial sediment	2015 September/ hand auger	Mattes (2018)[4]
	AWL-D1	37.114233, -79.273324	Surficial sediment		
	AWL-D2	37.114107, -79.272954	Surficial sediment		
	AWL-E2	37.114005, -79.272633	Surficial sediment		
	AWL-F4	37.114210, -79.272076	Surficial sediment		
Indiana Harbor and Ship Canal, Indiana, USA	IHSC-12-18”	41.645708, -87.472128	1 – 1.5 feet or 30 – 46 cm	2009 May 08/ a submersible vibrocoring system with a PVC tube.	Martinez (2011)[5]
	IHSC-54-60”	41.645708, -87.472128	4.5 – 5 feet or 137 – 152 cm		

*See more detailed information in Patrick Curran (2019), https://semspub.epa.gov/src/document/01/100012483.pdf.[2] The collection method was the same as that in Battelle Memorial Institute (2015).[3] The collection date was notified separately by Tony Silva PE from the United States Army Corps of Engineers (USACE), New England District and Dave Lederer from US EPA Region 1.

The detailed instrument method.

Gas chromatography (GC) coupled with mass spectrometry (MS) was employed for identification and quantification of PCBs and OH-PCBs. PCBs were analyzed with Agilent 7890A GC equipped with Supelco SPB-Octyl capillary column (30 m, 0.25 mm i.d., 0.25 μm film thickness) coupled with Agilent 7000B Triple Quadrupole (QqQ) MS system.[6] OH-PCBs were derivatized with diazomethane to MeO-PCBs and analyzed with Agilent 7890B GC equipped with Supelco SPB-Octyl capillary column coupled with Agilent 7000D QqQ MS system. The concentration of each PCB and OH-PCB congener in the calibration standard is ~50 ng/mL.

Table S4. GC injection temperature program.

Five μL of the standard and samples were injected into the GC-EI-MS system in multimode injection (also known as large volume injection) at 4.4 psi with septum purge flow of 1 mL/min and the following temperature program:

Rate (°C/min)	Target (°C)	Hold Time (min)	Run Time (min)
—	45	0.06	0.06
600	325	5	70.3

Table S5. GC elution temperature program.

PCBs and OH-PCBs as MeO-PCBs were eluted from the column with helium (0.8 mL min^{-1}) and the following temperature program:

Rate (°C/min)	Target (°C)	Hold Time (min)	Run Time (min)
—	45	2	2
100	75	5	7.3
15	150	1	13.3
2.5	280	5	70.3

Table S6. The isotopic molecular masses of MeO-PCBs and (their distributions) used to identify OH-PCBs.

MeO-PCBs	#Cl	#^{37}Cl									
		0	1	2	3	4	5	6	7	8	9
		M	M+2	M+4	M+6	M+8	M+10	M+12	M+14	M+16	M+18
Mono	1	218.0 (76%)	220.0 (24%)								
Di	2	252.0 (57%)	254.0 (37%)	256.0 (6%)							
Tri	3	286.0 (44%)	288.0 (42%)	290.0 (13%)	292.0 (1%)						
Tetra	4	319.9 (33%)	321.9 (42%)	323.9 (20%)	325.9 (4%)	327.9 (<1%)					
Penta	5	353.9 (25%)	355.9 (40%)	357.9 (26%)	359.9 (8%)	361.9 (1%)	363.9 (<1%)				
Hexa	6	387.9 (19%)	389.9 (36%)	391.8 (29%)	393.8 (12%)	395.8 (3%)	397.8 (<1%)	399.8 (<1%)			
Hepta	7	421.8 (14%)	423.8 (32%)	425.8 (31%)	427.8 (16%)	429.8 (5%)	431.8 (1%)	433.8 (<1%)	435.8 (<1%)		
Octa	8	455.8 (11%)	457.8 (28%)	459.8 (31%)	461.8 (20%)	463.8 (8%)	465.8 (2%)	467.8 (<1%)	469.8 (<1%)	471.8 (<1%)	
Nona	9	489.7 (8%)	491.7 (24%)	493.7 (30%)	495.7 (23%)	497.7 (11%)	499.7 (3%)	501.7 (1%)	503.7 (<1%)	505.7 (<1%)	507.7 (<1%)

- The isotopic molecular masses of MeO-PCBs were calculated based on their molecular formula:

$$^{12}C_{13}\ ^{35}Cl_{(\#Cl-\#^{37}Cl)}\ ^{37}Cl_{\#^{37}Cl}\ ^{1}H_{(12-\#Cl)}\ ^{16}O_{1}$$

where #Cl is the number of chlorines in the molecule or homolog groups, and #^{37}Cl is the number of ^{37}Cl in the molecule; and the isotopic mass of ^{12}C is 12.000000, ^{35}Cl is 34.968853, ^{37}Cl is 36.965903, ^{1}H is 1.007825, and ^{16}O is 15.994915.[7] The contribution of ^{13}C at X + 1 the to the isotopic pattern at X + 2 was considered negligible in this simple estimation where X is an isotopic molecular mass.

- The isotopic distribution of MeO-PCBs were calculated based mainly on ^{35}Cl and ^{37}Cl with the equation:

$$(75.77\% + 24.23\%)^{\#Cl} = \sum_{\#^{37}Cl=0}^{\#Cl} \binom{\#Cl}{\#^{37}Cl} 75.77\,\%^{(\#Cl-\#^{37}Cl)}\, 24.24^{\#^{37}Cl}$$

where 75.77% and 24.23% are respectively the isotopic abundances of ^{35}Cl and ^{37}Cl,[7] #Cl is the number of chlorines in the molecule; and #^{37}Cl is the number of ^{37}Cl in the molecule.

Table S7. Surrogate standard recoveries of method blanks.

$^{13}C_{12}$-SS*	#Cl	Method Blanks of Sediment Samples			
		1st	2nd	3rd	
$^{13}C_{12}$-PCB1	1	57%	56%	61%	
$^{13}C_{12}$-PCB15	2	51%	59%	61%	
$^{13}C_{12}$-PCB31	3	62%	73%	65%	
$^{13}C_{12}$-PCB52	4	71%	73%	67%	
$^{13}C_{12}$-PCB118	5	67%	87%	76%	
$^{13}C_{12}$-PCB153	6	85%	88%	82%	
$^{13}C_{12}$-PCB180	7	82%	92%	82%	
$^{13}C_{12}$-PCB194	8	82%	97%	93%	
$^{13}C_{12}$-PCB206	9	83%	96%	100%	
$^{13}C_{12}$-PCB209	10	89%	102%	109%	
$^{13}C_{12}$-4'-OH-PCB12	2	61%	41%	23%	
$^{13}C_{12}$-4'-OH-PCB29	3	63%	62%	49%	
$^{13}C_{12}$-4'-OH-PCB61	4	57%	63%	63%	
$^{13}C_{12}$-4'-OH-PCB120	5	103%	103%	79%	
$^{13}C_{12}$-4'-OH-PCB159	6	94%	70%	82%	
$^{13}C_{12}$-4-OH-PCB187	7	114%	77%	96%	
$^{13}C_{12}$-4'-OH-PCB172	7	90%	85%	81%	
**$^{13}C_{12}$-SS***	**#Cl**	**Method Blanks of Aroclor Samples**			
		**1st**	**2nd**	**3rd**	
$^{13}C_{12}$-PCB1	1	100%	100%	100%	
$^{13}C_{12}$-PCB15	2	104%	98%	98%	
$^{13}C_{12}$-PCB31	3	103%	100%	97%	
$^{13}C_{12}$-PCB52	4	101%	99%	100%	
$^{13}C_{12}$-PCB118	5	103%	99%	98%	
$^{13}C_{12}$-PCB153	6	100%	100%	100%	
$^{13}C_{12}$-PCB180	7	101%	99%	100%	
$^{13}C_{12}$-PCB194	8	100%	99%	101%	
$^{13}C_{12}$-PCB206	9	100%	99%	101%	
$^{13}C_{12}$-PCB209	10	100%	99%	100%	
$^{13}C_{12}$-4'-OH-PCB12	2	78%	65%	91%	88%
$^{13}C_{12}$-4'-OH-PCB29	3	76%	71%	93%	74%
$^{13}C_{12}$-4'-OH-PCB61	4	68%	98%	91%	64%
$^{13}C_{12}$-4'-OH-PCB120	5	94%	92%	107%	123%
$^{13}C_{12}$-4'-OH-PCB159	6	91%	77%	101%	121%
$^{13}C_{12}$-4-OH-PCB187	7	90%	101%	99%	116%
$^{13}C_{12}$-4'-OH-PCB172	7	97%	78%	99%	111%

*PCB congener names are in accordance with the US EPA: Table of PCB Species by Congener Number.[1]

Table S8. Surrogate standards recoveries of samples.

$^{13}C_{12}$-SS*	#Cl	The Upper Harbor of New Bedford Harbor					Altavista Wastewater Lagoon					Indiana Harbor and Ship Canal	
		O-287-A	O-288-A	O-288-B	O-331-A	O-331-C	AWL-C3	AWL-D1	AWL-D2	AWL-E2	AWL-F4	IHSC-12-18”	IHSC-54-60”
$^{13}C_{12}$-PCB1	1	46%	76%	74%	82%	94%	58%	63%	51%	73%	92%	60%	79%
$^{13}C_{12}$-PCB15	2	71%	149%	121%	123%	126%	68%	72%	98%	151%	180%	97%	131%
$^{13}C_{12}$-PCB31	3	79%	135%	95%	95%	90%	84%	83%	87%	140%	121%	105%	110%
$^{13}C_{12}$-PCB52	4	66%	104%	96%	96%	93%	83%	88%	64%	106%	93%	95%	93%
$^{13}C_{12}$-PCB118	5	96%	162%	133%	142%	142%	91%	92%	114%	198%	188%	125%	121%
$^{13}C_{12}$-PCB153	6	70%	108%	99%	101%	105%	99%	95%	61%	104%	91%	97%	109%
$^{13}C_{12}$-PCB180	7	84%	131%	109%	110%	116%	101%	97%	75%	128%	115%	102%	105%
$^{13}C_{12}$-PCB194	8	120%	160%	119%	120%	125%	103%	101%	94%	158%	146%	104%	96%
$^{13}C_{12}$-PCB206	9	105%	155%	107%	108%	113%	100%	98%	80%	156%	118%	98%	84%
$^{13}C_{12}$-PCB209	10	104%	140%	99%	97%	103%	100%	96%	72%	146%	94%	97%	80%
$^{13}C_{12}$-4'-OH-PCB12	2	208%	186%	154%	241%	169%	95%	105%	204%	174%	168%	104%	122%
$^{13}C_{12}$-4'-OH-PCB29	3	158%	167%	127%	186%	134%	111%	96%	181%	192%	181%	100%	101%
$^{13}C_{12}$-4'-OH-PCB61	4	181%	165%	156%	195%	137%	116%	101%	227%	182%	196%	113%	119%
$^{13}C_{12}$-4'-OH-PCB120	5	105%	96%	115%	109%	90%	131%	130%	141%	150%	142%	89%	110%
$^{13}C_{12}$-4'-OH-PCB159	6	103%	95%	91%	90%	81%	102%	129%	169%	158%	157%	74%	90%
$^{13}C_{12}$-4-OH-PCB187	7	96%	82%	82%	85%	80%	85%	104%	130%	142%	119%	34%	71%
$^{13}C_{12}$-4'-OH-PCB172	7	102%	97%	99%	85%	82%	86%	103%	139%	129%	142%	43%	62%

$^{13}C_{12}$-SS*	#Cl	Aroclor 1016			Aroclor 1242			Aroclor 1248			Aroclor 1254		
		1st	2nd	3rd	1st	2nd	3rd	1st	2nd	3rd	1st	2nd	3rd
$^{13}C_{12}$-PCB1	1	99%	98%	96%	94%	92%	92%	97%	92%	93%	93%	96%	96%
$^{13}C_{12}$-PCB15	2	118%	118%	110%	110%	103%	107%	112%	108%	109%	86%	91%	92%
$^{13}C_{12}$-PCB31	3	130%	130%	125%	127%	123%	126%	125%	122%	123%	98%	100%	101%
$^{13}C_{12}$-PCB52	4	104%	104%	100%	104%	101%	103%	117%	116%	118%	103%	102%	104%
$^{13}C_{12}$-PCB118	5	94%	97%	90%	102%	94%	101%	121%	117%	116%	124%	131%	130%
$^{13}C_{12}$-PCB153	6	100%	101%	100%	101%	101%	102%	101%	104%	105%	113%	112%	112%
$^{13}C_{12}$-PCB180	7	98%	100%	96%	99%	97%	99%	92%	93%	92%	106%	107%	107%
$^{13}C_{12}$-PCB194	8	97%	99%	95%	97%	93%	98%	95%	93%	90%	100%	102%	101%
$^{13}C_{12}$-PCB206	9	97%	97%	94%	95%	93%	95%	92%	92%	88%	95%	97%	96%
$^{13}C_{12}$-PCB209	10	97%	96%	93%	94%	92%	94%	96%	96%	90%	92%	92%	92%
$^{13}C_{12}$-4'-OH-PCB12	2	78%	65%	91%	76%	77%	65%	120%	105%	119%	85%	33%	80%
$^{13}C_{12}$-4'-OH-PCB29	3	76%	71%	93%	76%	70%	68%	91%	89%	100%	81%	40%	81%
$^{13}C_{12}$-4'-OH-PCB61	4	68%	98%	91%	69%	107%	64%	76%	80%	87%	72%	35%	84%
$^{13}C_{12}$-4'-OH-PCB120	5	94%	92%	107%	95%	105%	81%	128%	123%	124%	102%	65%	92%
$^{13}C_{12}$-4'-OH-PCB159	6	91%	77%	101%	98%	101%	74%	124%	118%	137%	105%	85%	83%
$^{13}C_{12}$-4-OH-PCB187	7	90%	101%	99%	95%	102%	79%	121%	113%	104%	94%	85%	83%
$^{13}C_{12}$-4'-OH-PCB172	7	97%	78%	99%	100%	97%	71%	114%	122%	105%	99%	99%	77%

We used the surrogate standard recoveries to correct the PCB and OH-PCB masses in samples and method blanks. The recoveries can be below 100% due to the loss during extraction or can be over 100% due to the effect of sample matrix and the solvent evaporation in multimode injection.
*PCB congener names are in accordance with the US EPA: Table of PCB Species by Congener Number.[1]

Table S9. LOQs of PCBs in sediments and Aroclors.

Congener	#Cl	Sediment (ng/g)	Aroclor (g/sample)
PCB1	1	0.41	0.14
PCB2	1	0.10	0.20
PCB3	1	0.25	0.13
PCB4	2	10	0.08
PCB5	2	0.91	0.13
PCB6	2	10	0.07
PCB7	2	0.60	0.06
PCB8	2	11	0.08
PCB9	2	0.82	0.07
PCB10	2	0.76	0.08
PCB11	2	0.98	0.09
PCB12 + PCB13	2	3.2	0.12
PCB14	2	0.19	0.08
PCB15	2	4.6	0.07
PCB16	3	0.52	0.09
PCB17	3	8.1	0.11
PCB18 + PCB30	3	18	0.19
PCB19	3	2.9	0.06
PCB20 + PCB28	3	13	0.21
PCB21 + PCB33	3	5.5	0.23
PCB22	3	1.3	0.16
PCB23	3	0.07	0.06
PCB24	3	0.43	0.05
PCB25	3	8.3	0.06
PCB26 + PCB29	3	11	0.12
PCB27	3	4.3	0.05
PCB31	3	18	0.14
PCB32	3	6.1	0.07
PCB34	3	0.14	0.05
PCB35	3	0.11	0.11
PCB36	3	0.31	0.14
PCB37	3	0.12	0.10
PCB38	3	0.08	0.07
PCB39	3	0.16	0.15
PCB40 + PCB71	4	2.3	0.17
PCB41	4	0.25	0.11
PCB42	4	0.79	0.22
PCB43	4	0.32	0.14
PCB44 + PCB47 + PCB65	4	4.6	0.30
PCB45	4	0.44	0.15
PCB46	4	0.91	0.13
PCB48	4	0.68	0.12
PCB49 + PCB69	4	8.7	0.23
PCB50 + PCB53	4	3.4	0.27
PCB51	4	1.1	0.08
PCB52	4	15	0.12
PCB54	4	0.11	0.12
PCB55	4	0.23	0.10
PCB56	4	0.18	0.11
PCB57	4	0.14	0.10
PCB58	4	0.10	0.06
PCB59 + PCB62 + PCB75	4	0.63	0.24
PCB60	4	0.17	0.12
PCB61 + PCB70 + PCB74 + PCB76	4	1.1	0.32
PCB63	4	0.12	0.11
PCB64	4	0.80	0.13
PCB66	4	0.48	0.15
PCB67	4	0.08	0.09
PCB68	4	0.19	0.08
PCB72	4	0.25	0.12
PCB73	4	0.63	0.09
PCB77	4	0.06	0.09

Continued on next page.

Table S9 (Continued). LOQs of PCBs in sediments and Aroclors.

Congener	#Cl	Sediment (ng/g)	Aroclor (g/sample)
PCB78	4	0.07	0.07
PCB79	4	0.10	0.09
PCB80	4	0.08	0.11
PCB81	4	0.10	0.08
PCB82	5	0.25	0.11
PCB83	5	0.27	0.09
PCB84	5	1.4	0.09
PCB85 + PCB116	5	0.11	0.14
PCB86 + PCB97 + PCB109 + PCB119	5	0.88	0.28
PCB87 + PCB125	5	0.47	0.19
PCB88	5	0.16	0.08
PCB89	5	0.08	0.08
PCB90 + PCB101 + PCB113	5	1.8	0.22
PCB91	5	0.82	0.07
PCB92	5	0.60	0.09
PCB93 + PCB100	5	0.29	0.13
PCB94	5	0.06	0.08
PCB95	5	3.4	0.09
PCB96	5	0.13	0.09
PCB98	5	0.14	0.05
PCB99	5	0.66	0.09
PCB102	5	0.37	0.13
PCB103	5	0.21	0.09
PCB104	5	0.05	0.08
PCB105	5	0.29	0.17
PCB106	5	0.05	0.07
PCB107	5	0.06	0.07
PCB108 + PCB124	5	0.10	0.11
PCB110	5	1.6	0.06
PCB111	5	0.12	0.07
PCB112	5	0.20	0.08
PCB114	5	0.11	0.10
PCB115	5	0.50	0.13
PCB117	5	0.11	0.09
PCB118	5	0.70	0.15
PCB120	5	0.04	0.08
PCB121	5	0.06	0.06
PCB122	5	0.14	0.09
PCB123	5	0.03	0.09
PCB126	5	0.02	0.06
PCB127	5	0.08	0.08
PCB128 + PCB166	6	0.08	0.16
PCB129 + PCB138 + PCB163	6	0.53	0.19
PCB130	6	0.06	0.07
PCB131	6	0.09	0.08
PCB132	6	0.18	0.09
PCB133	6	0.07	0.06
PCB134	6	0.07	0.10
PCB135 + PCB151	6	0.54	0.14
PCB136	6	0.35	0.06
PCB137	6	0.05	0.07
PCB139 + PCB140	6	0.08	0.15
PCB141	6	0.09	0.08
PCB142	6	0.06	0.07
PCB143	6	0.08	0.07
PCB144	6	0.06	0.09
PCB145	6	0.05	0.07
PCB146	6	0.16	0.06
PCB147 + PCB149	6	1.6	0.14
PCB148	6	0.07	0.08
PCB150	6	0.06	0.06
PCB152	6	0.04	0.07

Continued on next page.

Table S9 (Continued). LOQs of PCBs in sediments and Aroclors.

Congener	#Cl	Sediment (ng/g)	Aroclor (g/sample)
PCB153 + PCB168	6	0.66	0.13
PCB154	6	0.09	0.08
PCB155	6	0.06	0.06
PCB156 + PCB157	6	0.05	0.02
PCB158	6	0.07	0.07
PCB159	6	0.03	0.06
PCB160	6	0.06	0.08
PCB161	6	0.08	0.07
PCB162	6	0.02	0.09
PCB164	6	0.05	0.06
PCB165	6	0.04	0.06
PCB167	6	0.05	0.06
PCB169	6	0.03	0.02
PCB170	7	0.04	0.06
PCB171 + PCB173	7	0.08	0.18
PCB172	7	0.12	0.09
PCB174	7	0.07	0.06
PCB175	7	0.03	0.07
PCB176	7	0.04	0.07
PCB177	7	0.05	0.09
PCB178	7	2.3	0.08
PCB179	7	0.12	0.06
PCB180 + PCB193	7	0.13	0.15
PCB181	7	0.64	0.58
PCB182	7	1.8	1.2
PCB183	7	0.07	0.06
PCB184	7	2.4	1.4
PCB185	7	0.03	0.07
PCB186	7	0.27	0.20
PCB187	7	0.26	0.06
PCB188	7	1.4	0.81
PCB189	7	0.06	0.04
PCB190	7	0.05	0.06
PCB191	7	0.05	0.07
PCB192	7	0.05	0.08
PCB194	8	0.06	0.07
PCB195	8	0.08	0.10
PCB196	8	0.03	0.07
PCB197	8	0.17	0.12
PCB198 + PCB199	8	0.09	0.14
PCB200	8	0.16	0.10
PCB201	8	0.04	0.23
PCB202	8	0.04	0.38
PCB203	8	0.06	0.07
PCB205	8	0.04	0.05
PCB206	9	0.09	0.05
PCB207	9	0.03	0.06
PCB208	9	0.03	0.05
PCB209	10	0.42	0.51

PCB congener names are in accordance with the US EPA: Table of PCB Species by Congener Number.[1]

Table S10. LOQs of known OH-PCBs in sediments and Aroclors.

Congener	#Cl	Sediment (ng/g)	Aroclor (g/sample)
4-OH-PCB1	1	0.05	0.35
2-OH-PCB2	1	0.03	0.30
2'-OH-PCB2	1	0.02	0.29
3'-OH-PCB2	1	0.03	0.30
4-OH-PCB2	1	0.74	1.4
4'-OH-PCB2	1	0.41	0.16
5-OH-PCB2	1	0.03	0.31
6-OH-PCB2	1	0.02	0.26
4'-OH-PCB3	1	0.07	0.24
2'-OH-PCB5	2	0.06	0.23
3'-OH-PCB9	2	0.04	0.21
2'-OH-PCB12	2	0.08	0.57
4-OH-PCB14 + 4'-OH-PCB9	2	0.11	0.00
4'-OH-PCB18	3	0.14	0.66
4'-OH-PCB26	3	0.02	0.66
6'-OH-PCB26	3	0.01	0.21
2'-OH-PCB30	3	0.02	0.18
3'-OH-PCB30	3	0.02	0.34
4'-OH-PCB30	3	0.02	0.36
3-OH-PCB54	4	0.02	0.71
2'-OH-PCB61	4	0.06	0.74
3'-OH-PCB61	4	0.02	2.1
4'-OH-PCB61	4	0.01	1.1
2'-OH-PCB65 + 6'-OH-PCB69	4	0.03	0.44
3'-OH-PCB65	4	0.04	0.83
4-OH-PCB65	4	0.02	0.55
4'-OH-PCB65	4	0.06	1.1
4'-OH-PCB69	4	0.04	0.75
4'-OH-PCB72	4	0.02	3.0
4'-OH-PCB79	4	0.02	5.0
6'-OH-PCB83	5	0.01	0.12
4'-OH-PCB86	5	0.02	0.92
4'-OH-PCB93	5	0.04	0.42
4'-OH-PCB97	5	0.01	0.44
4'-OH-PCB101	5	0.01	0.38
6'-OH-PCB101	5	0.03	0.31
2'-OH-PCB106 + 2'-OH-PCB114	5	0.03	0.19
4-OH-PCB107	5	0.02	1.6
4'-OH-PCB108	5	0.02	1.4
3-OH-PCB118	5	0.02	0.49
4'-OH-PCB120	5	0.01	1.4
4'-OH-PCB127	5	0.04	2.5
4'-OH-PCB130	6	0.02	0.67
4-OH-PCB134	6	0.04	0.81
3'-OH-PCB138	6	0.02	0.66
5-OH-PCB138	6	0.06	1.1
4-OH-PCB146	6	0.02	0.59
4'-OH-PCB159	6	0.03	1.8
4-OH-PCB162	6	0.05	1.8
4-OH-PCB163	6	0.03	0.94
4'-OH-PCB172	7	0.03	0.80
4-OH-PCB177	7	0.03	0.77
4-OH-PCB178	7	0.02	0.65
3'-OH-PCB180	7	0.04	0.32
3'-OH-PCB182	7	0.02	1.5
3'-OH-PCB183	7	0.03	0.48
5-OH-PCB183 + 4-OH-PCB187	7	0.05	0.85
3'-OH-PCB184	7	0.03	1.5
4-OH-PCB193	7	0.04	0.64
4'-OH-PCB198 + 4'-OH-PCB200 + 3'-OH-PCB203	8	0.09	0.76
4'-OH-PCB199	8	0.04	0.74
4'-OH-PCB201	8	0.03	0.80
4-OH-PCB202	8	0.03	0.37
4'-OH-PCB208	9	0.04	0.52

PCB congener names are in accordance with the US EPA: Table of PCB Species by Congener Number.[1]

Table S11. The recoveries of OH-PCB surrogate standards in IHSC-66-72” sediment.

$^{13}C_{12}$-SS*	#Cl	1st	2nd
$^{13}C_{12}$-4'-OH-PCB12	2	0%	0%
$^{13}C_{12}$-4'-OH-PCB29	3	0%	0%
$^{13}C_{12}$-4'-OH-PCB61	4	0%	0%
$^{13}C_{12}$-4'-OH-PCB120	5	96%	107%
$^{13}C_{12}$-4'-OH-PCB159	6	80%	82%
$^{13}C_{12}$-4-OH-PCB187	7	43%	63%
$^{13}C_{12}$-4'-OH-PCB172	7	58%	70%

There were 3 IHSC sediments available: IHSC-12-18”, IHSC-54-60”, and IHSC-66-72”. We excluded IHSC-66-72” from the report due to the zero recovery in some OH-PCB surrogate standards as shown in the table.
During the extraction method development, we found that sulfuric acid decreased the recoveries of some OH-PCB surrogate standards. The OH-PCB surrogate standards whose ortho-positions of OH were hydrogen always showed low or zero recovery, while the others whose ortho-positions of OH were chlorine showed high recovery.
First, we suspected the difference of pKa between OH-PCBs. However, when hydrochloric acid was used, all OH-PCB surrogate standards showed high recovery. Indeed, we selected hydrochloric acid for our extraction method.
We later found that the sulfonation of phenol by concentrated sulfuric acid is a common reaction.[8] We hypothesized that sulfuric acid reacted with the OH-PCBs whose ortho-positions of OH were hydrogen, thereby flattening OH-PCB surrogate standards.
We speculated that the zero recoveries of OH-PCB surrogate standards in the IHSC-66-72” sediment was due to the high sulfate content in the sample. Sulfuric acid or sulfate was heavily used and released into IHSC along with a long history of steel industrials in East Chicago, IN.[9-11]

Table S12. The cosine similarity (cos θ) of OH-PCB congener profiles between pairs of samples.

Sample		The Upper Harbor of New Bedford Harbor					Altavista Wastewater Lagoon					Indiana Harbor and Ship Canal	
		O-287-A	O-288-A	O-288-B	O-331-A	O-331-C	AWL-C3	AWL-D1	AWL-D2	AWL-E2	AWL-F4	IHSC-12-18”	IHSC-54-60”
The Upper Harbor of New Bedford Harbor	O-287-A	NA	0.94	0.80	0.85	0.76	0.12	0.30	0.06	0.12	0.14	0.17	0.10
	O-288-A		NA	0.85	0.89	0.81	0.16	0.32	0.04	0.12	0.14	0.15	0.09
	O-288-B			NA	0.98	0.96	0.17	0.30	0.04	0.14	0.17	0.13	0.08
	O-331-A				NA	0.95	0.19	0.33	0.05	0.15	0.18	0.15	0.09
	O-331-C					NA	0.29	0.45	0.05	0.22	0.25	0.19	0.11
Altavista Wastewater Lagoon	AWL-C3						NA	0.84	0.18	0.64	0.60	0.24	0.21
	AWL-D1							NA	0.17	0.66	0.64	0.31	0.23
	AWL-D2								NA	0.68	0.57	0.46	0.77
	AWL-E2									NA	0.90	0.49	0.60
	AWL-F4										NA	0.54	0.56
Indiana Harbor and Ship Canal	IHSC-12-18”											NA	0.88
	IHSC-54-60”												NA

Sample		A1016			A1248			A1254		
		1st	2nd	3rd	1st	2nd	3rd	1st	2nd	3rd
A1016	1st	NA	0.96	0.98	0.06	0.06	0.05	0.06	0.07	0.09
	2nd		NA	0.98	0.06	0.07	0.06	0.07	0.09	0.10
	3rd			NA	0.07	0.07	0.06	0.07	0.08	0.10
A1248	1st				NA	0.99	0.99	0.20	0.16	0.26
	2nd					NA	0.99	0.21	0.17	0.28
	3rd						NA	0.18	0.14	0.24
A1254	1st							NA	0.92	0.96
	2nd								NA	0.91
	3rd									NA

Sample		A1016			A1248			A1254		
		1st	2nd	3rd	1st	2nd	3rd	1st	2nd	3rd
The Upper Harbor of New Bedford Harbor	O-287-A	0.12	0.12	0.13	0.09	0.09	0.09	0.10	0.10	0.13
	O-288-A	0.16	0.16	0.17	0.09	0.08	0.08	0.06	0.06	0.08
	O-288-B	0.19	0.20	0.21	0.10	0.09	0.09	0.08	0.08	0.10
	O-331-A	0.22	0.22	0.23	0.11	0.10	0.09	0.10	0.10	0.12
	O-331-C	0.31	0.29	0.30	0.10	0.10	0.08	0.10	0.10	0.12
Altavista Wastewater Lagoon	AWL-C3	0.30	0.29	0.27	0.02	0.02	0.02	0.02	0.02	0.02
	AWL-D1	0.54	0.52	0.50	0.03	0.04	0.03	0.04	0.06	0.06
	AWL-D2	0.04	0.06	0.05	0.03	0.03	0.03	0.07	0.07	0.08
	AWL-E2	0.30	0.30	0.26	0.04	0.05	0.04	0.08	0.09	0.09
	AWL-F4	0.34	0.34	0.30	0.10	0.11	0.10	0.10	0.11	0.12
Indiana Harbor and Ship Canal	IHSC-12-18”	0.15	0.16	0.14	0.04	0.04	0.04	0.13	0.13	0.14
	IHSC-54-60”	0.12	0.14	0.12	0.02	0.03	0.02	0.07	0.07	0.08

NA is the same sample.
In A1242, the unknown OH-PCB levels were all below LOQ, only known mono- and tri-chlorinated OH-PCBs were detected, and their concentrations were too low to construct a reliable profile; therefore, A1242 was excluded from cos θ analysis.

Table S13. The number of OH-PCB congener peaks found in sediments from NBH, AWL, and IHSC, and in Aroclors, by or chlorination or homolog.

#Cl		1	2	3	4	5	6	7	8	9	Total
**NBH**	Known	8	2	3	4	9	2	1	0	0	29
	Unknown	7	25	30	21	14	6	1	0	0	104
**AWL**	Known	8	2	3	5	7	1	0	0	0	26
	Unknown	3	16	29	23	8	1	0	0	0	80
**IHSC**	Known	9	1	1	4	5	3	2	0	0	25
	Unknown	1	10	14	11	0	0	0	0	0	36
**A1016**	Known	7	1	3	3	2	0	0	0	0	16
	Unknown	7	25	30	14	0	0	0	0	0	76
**A1242**	Known	2	0	2	0	0	0	0	0	0	4
	Unknown	0	0	0	0	0	0	0	0	0	0
**A1248**	Known	8	2	4	7	8	6	9	3	1	48
	Unknown	4	17	30	26	23	7	3	1	0	111
**A1254**	Known	7	1	3	5	9	5	7	0	0	37
	Unknown	3	17	35	34	38	10	5	0	0	142

*The numbers of known peaks are individual OH-PCBs identified with authentic standard in this study. The number of unknown peaks indicate individual or coeluting OH-PCBs of each homolog group that are not yet been identified with authentic standards.

Figure S1. Aroclors 1016, 1242, 1248, and 1254 used in this study. Photographs taken by the authors.

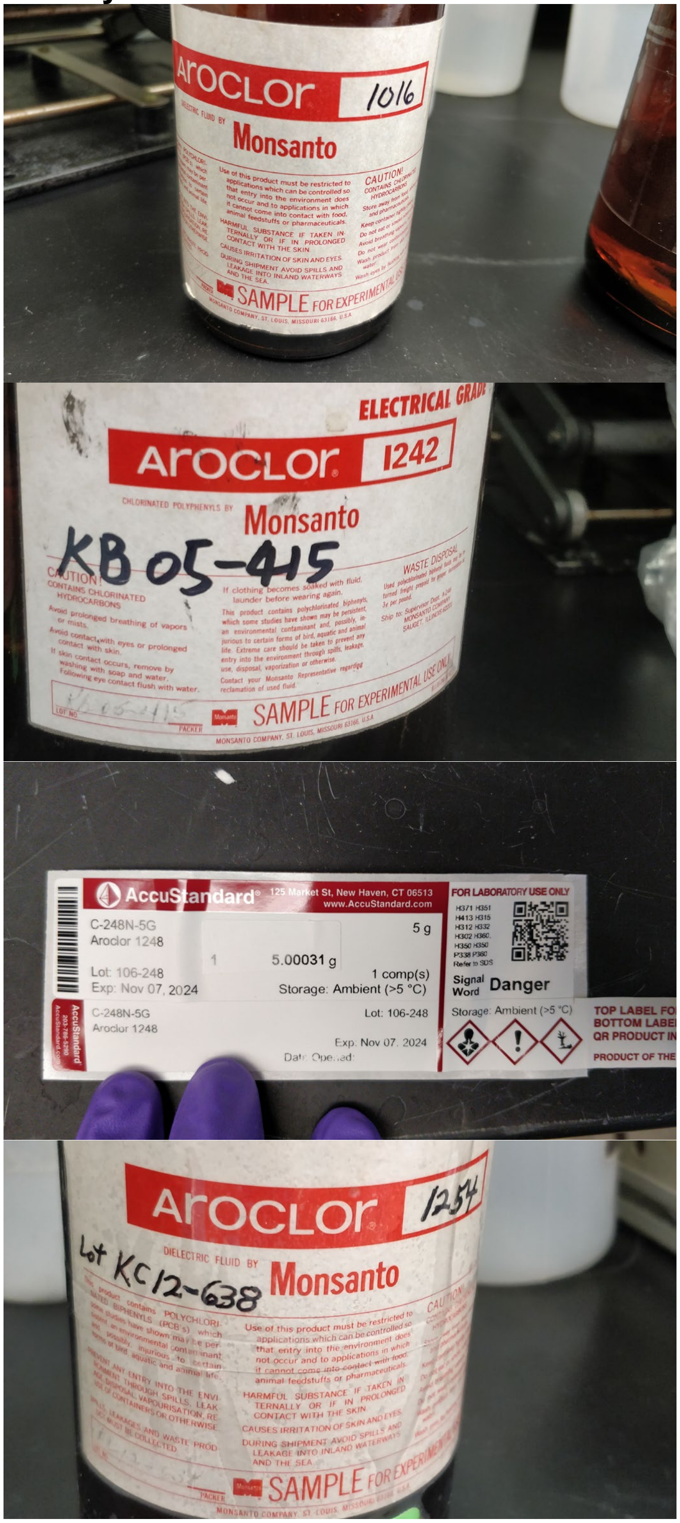

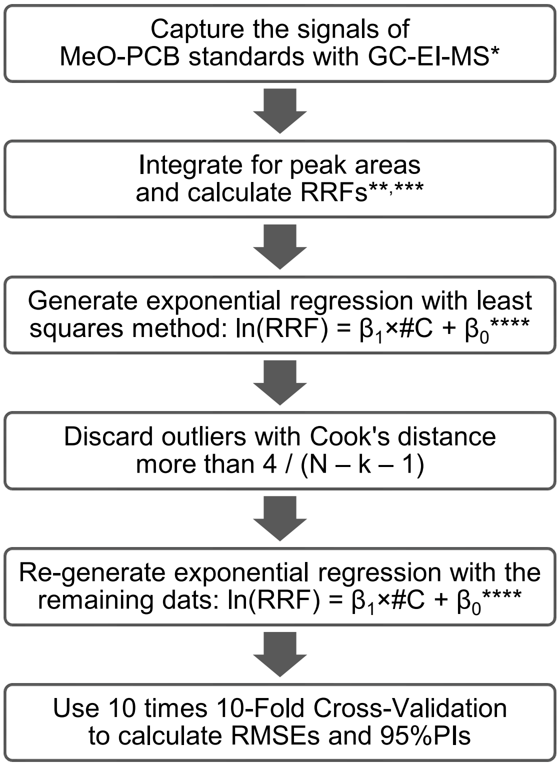

Figure S2. The procedure to generate a model to predict the relative response factors (RRFs) of unknown MeO-PCBs from the number of chlorines in the molecule or homolog groups (#Cl).

*The peak areas or peak responses were captured in positive electron ionization (EI) at 30 eV in selected ion monitoring (SIM) mode. Different instrument may need different energy level.

**Coeluting congeners are not included in the model.

***$\mathrm{RRF} = \frac{\text{Peak Area}_{\mathrm{d_5-PCB30\ IS}}/\text{Concentration}_{\mathrm{d_5-PCB30\ IS}}}{\text{Peak Area}_{\mathrm{MeO-PCB}}/\text{Concentration}_{\mathrm{MeO-PCB}}}$

****The predictive model was amended from quadratic regression in our previous report to exponential regression.[12] While RRF ⊂ (0,∞), the quadratic function ($RRF_{Quadratic} = \beta_1 \times \#Cl^2 + \beta_0$) provides $RRF_{Quadratic}$ ⊂ (-∞,∞) which is not corresponding to RRF. However, the exponential function ($\ln(RRF_{Exponetial}) = \beta_1 \times Cl\#^2 + \beta_0$) provides $\ln(RRF_{Exponetial})$ ⊂ (-∞,∞) or $RRF_{Exponetial}$ ⊂ (0,∞) which is corresponding to RRF.

Note: Root mean square errors (RMSEs); 95% prediction intervals (95%PIs); and gas chromatography coupling with electron impact mass spectrometry (GC-EI-MS).

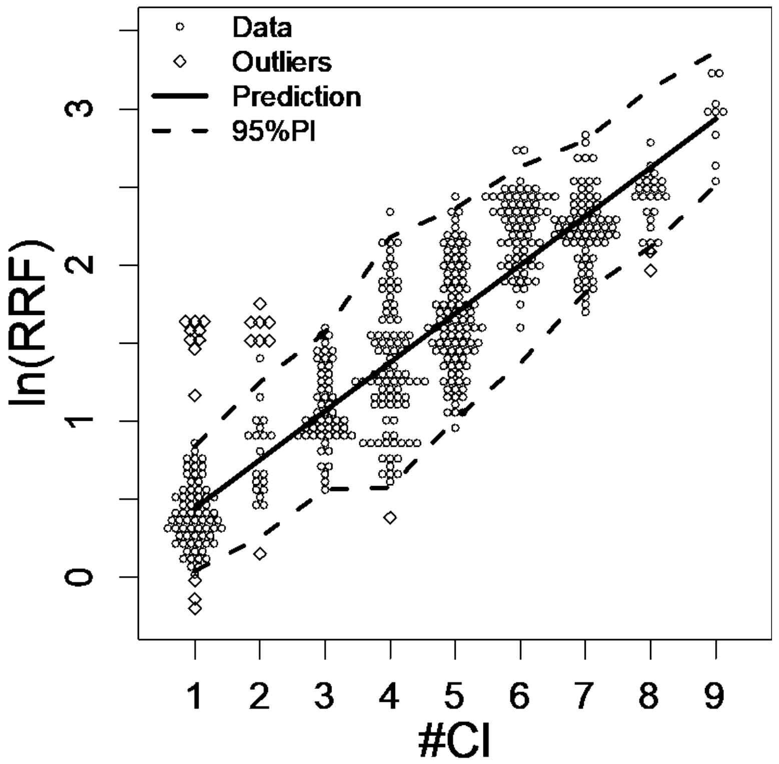

Figure S3. An example of exponential regression to predict the relative response factors (RRFs) of unknown MeO-PCBs from the number of chlorines in the molecule or homolog groups (#Cl).

To use this model, the peak areas of unknown OH-PCBs derivatized to MeO-PCBs in samples are converted to peak area ratios by dividing with the peak area of the d_5-PCB30 IS. The peak area ratios are then multiplied with the predicted RRF of the corresponding #Cl from the exponential regression and the added amount of d_5-PCB30 IS in samples which results in the predicted masses of MeOH-PCB in the samples.

95%PI = 95% Prediction Interval from 10 times 10-fold cross-validation

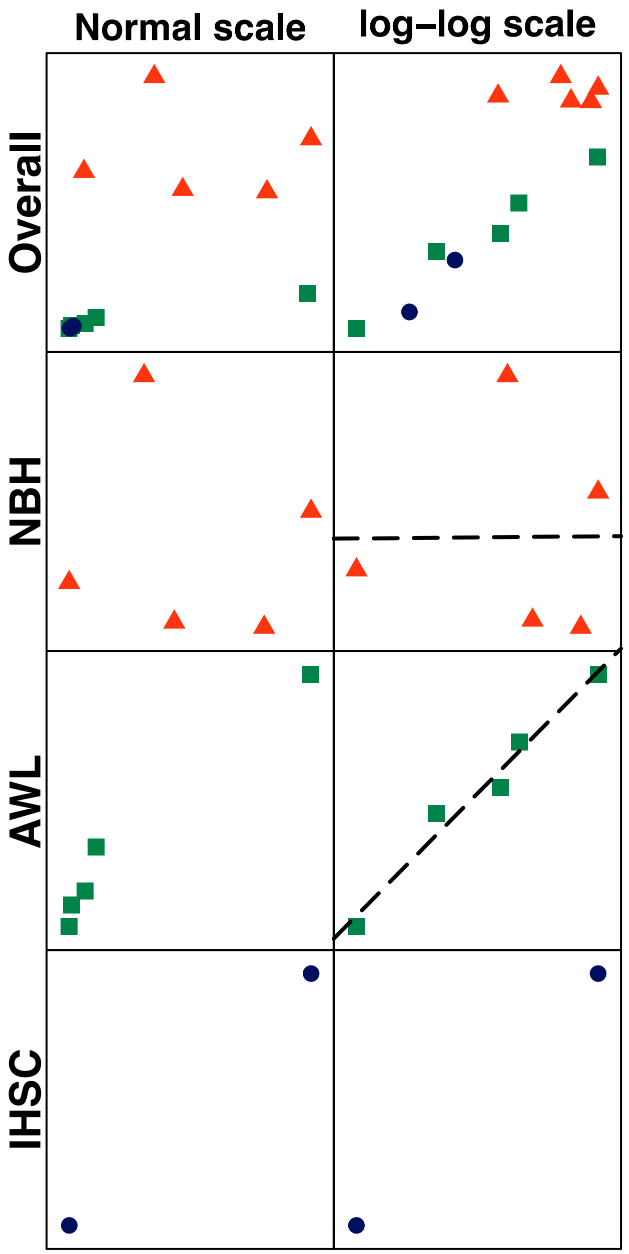

Figure S4. Correlations between OH-PCB (Y-axis) and PCB (X-axis) levels in sediments in normal scale (left panels) and in log-log- scale (right panels). All plots have different x-axis and y-axis scales and ranges.

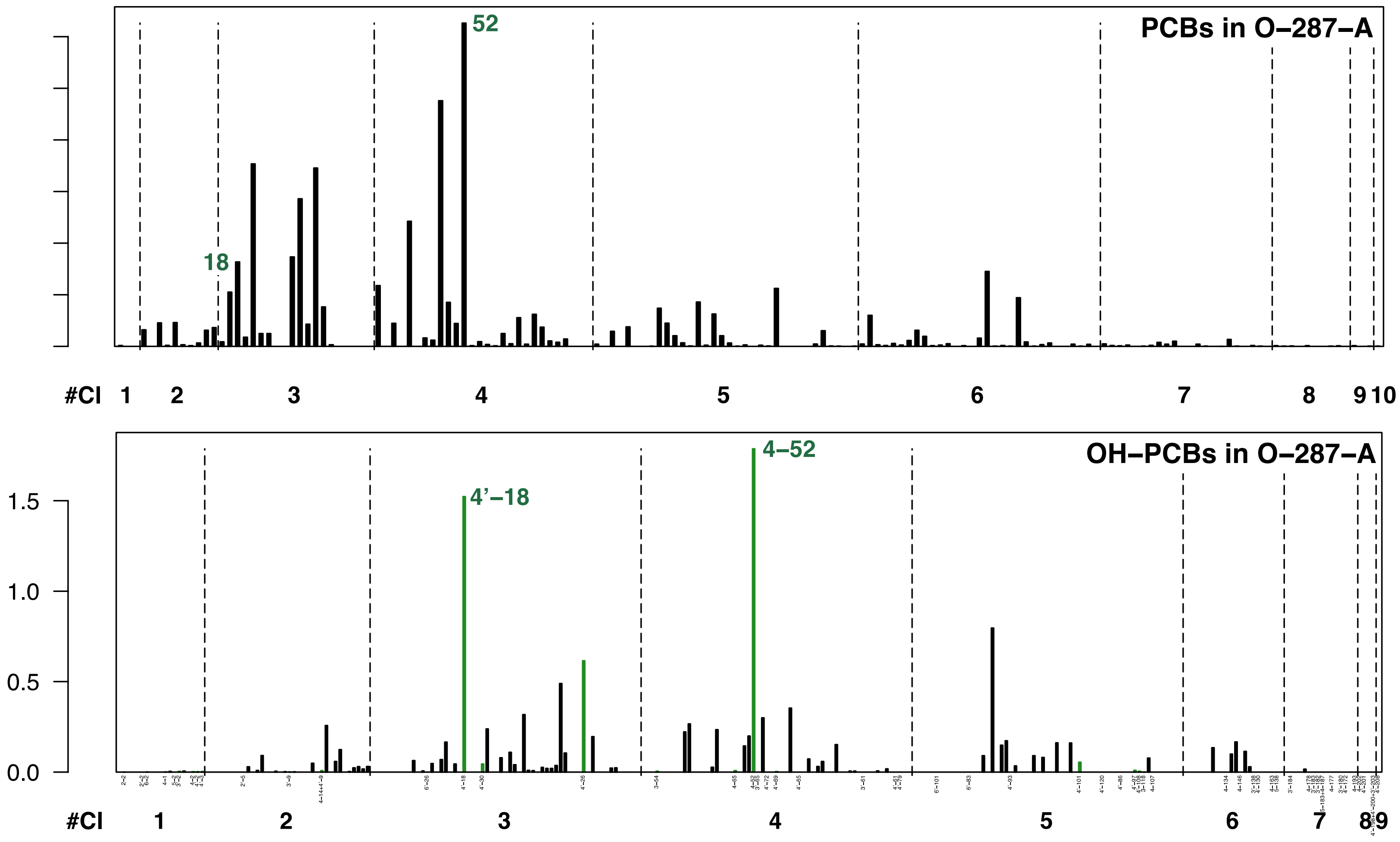

Figure S5. The congener profiles of PCB (above) and OH-PCBs (below) in O-287-A sediment from New Bedford Harbor (NBH) in µg/g dry weight.
OH-PCB congeners are arranged by homologs (#Cl) and peak elution order from Supelco SPB-Octyl capillary column with green bars indicating knowns and black bars indicating unknown.
Individual congener concentrations are available at Saktrakulkla et al. (2021).[13]

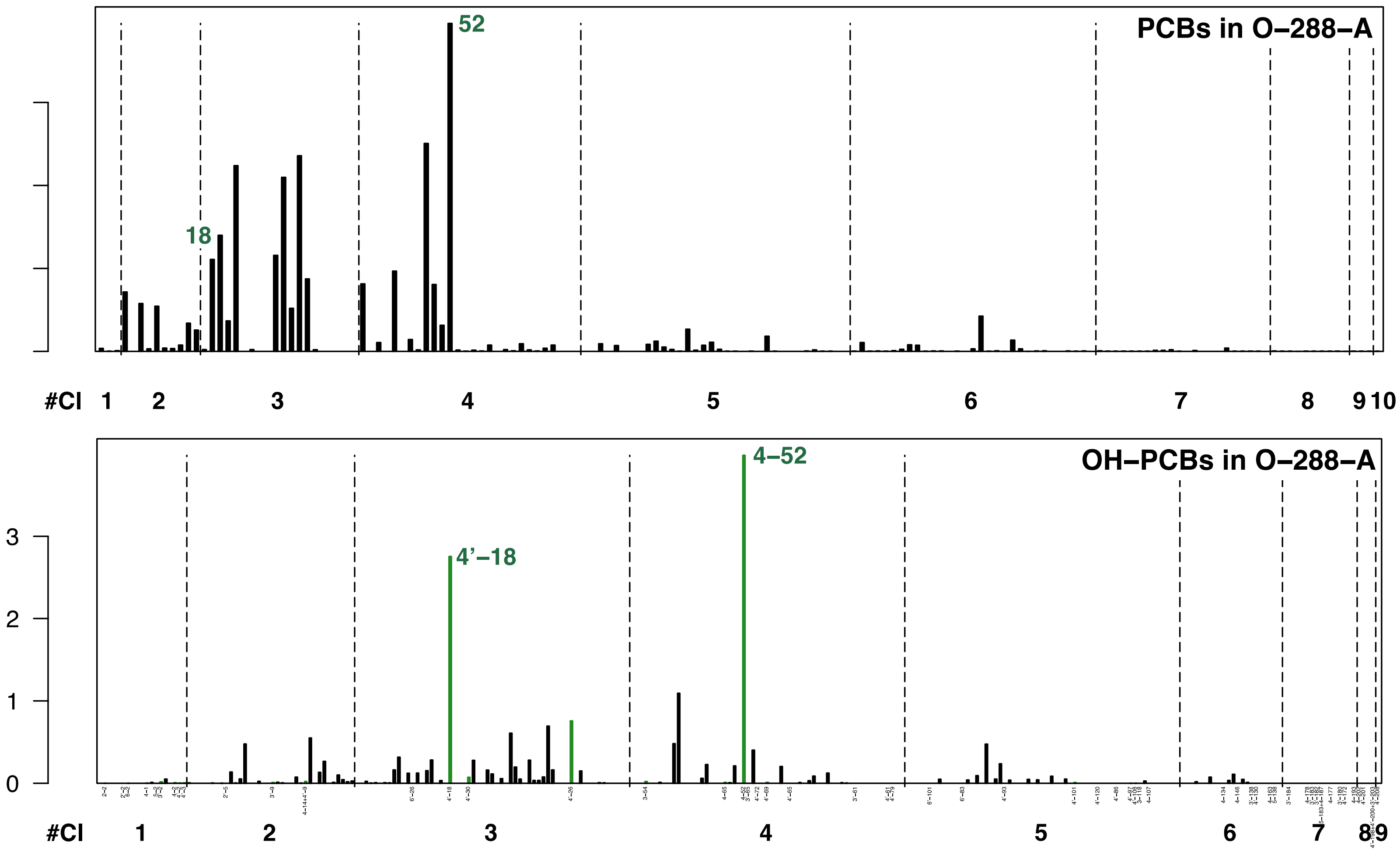

Figure S6. The congener profiles of PCB (above) and OH-PCBs (below) in O-288-A sediment from New Bedford Harbor (NBH) in µg/g dry weight.
OH-PCB congeners are arranged by homologs (#Cl) and peak elution order from Supelco SPB-Octyl capillary column with green bars indicating knowns and black bars indicating unknown.
Individual congener concentrations are available at Saktrakulkla et al. (2021).[13]

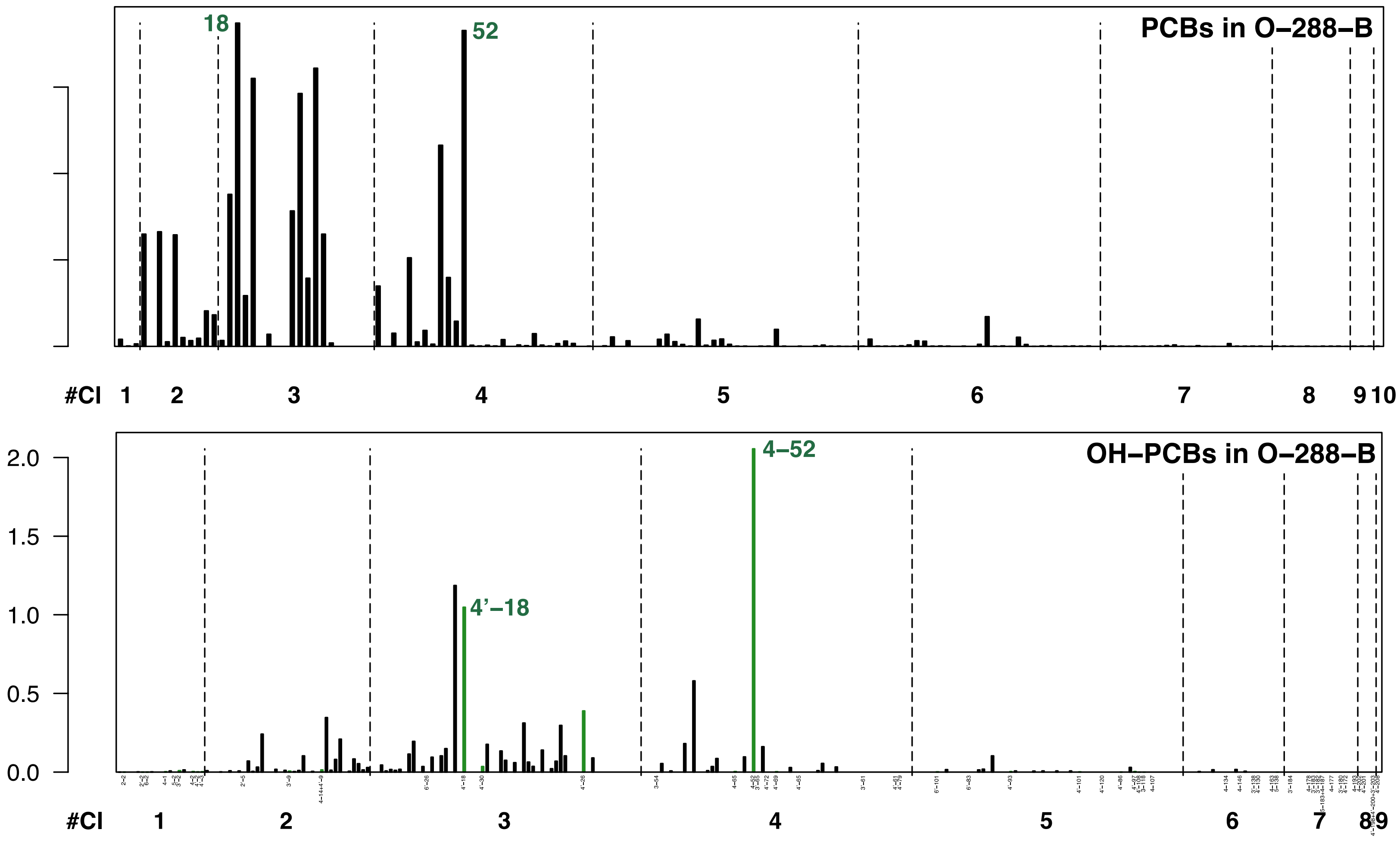

Figure S7. The congener profiles of PCB (above) and OH-PCBs (below) in O-288-B sediment from New Bedford Harbor (NBH) in µg/g dry weight.
OH-PCB congeners are arranged by homologs (#Cl) and peak elution order from Supelco SPB-Octyl capillary column with green bars indicating knowns and black bars indicating unknown.
Individual congener concentrations are available at Saktrakulkla et al. (2021).[13]

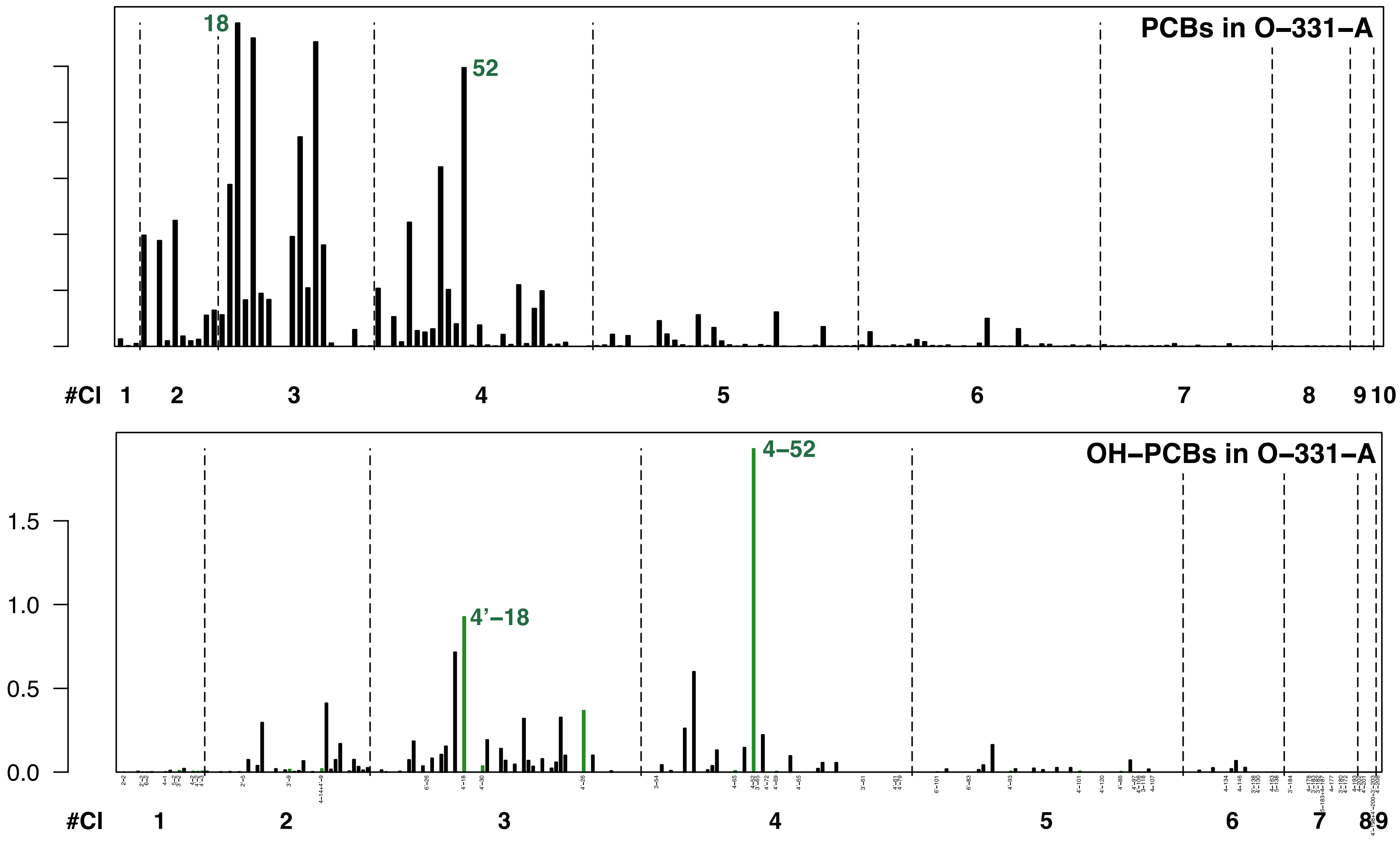

Figure S8. The congener profiles of PCB (above) and OH-PCBs (below) in O-331-A sediment from New Bedford Harbor (NBH) in µg/g dry weight.
OH-PCB congeners are arranged by homologs (#Cl) and peak elution order from Supelco SPB-Octyl capillary column with green bars indicating knowns and black bars indicating unknown.
Individual congener concentrations are available at Saktrakulkla et al. (2021).[13]

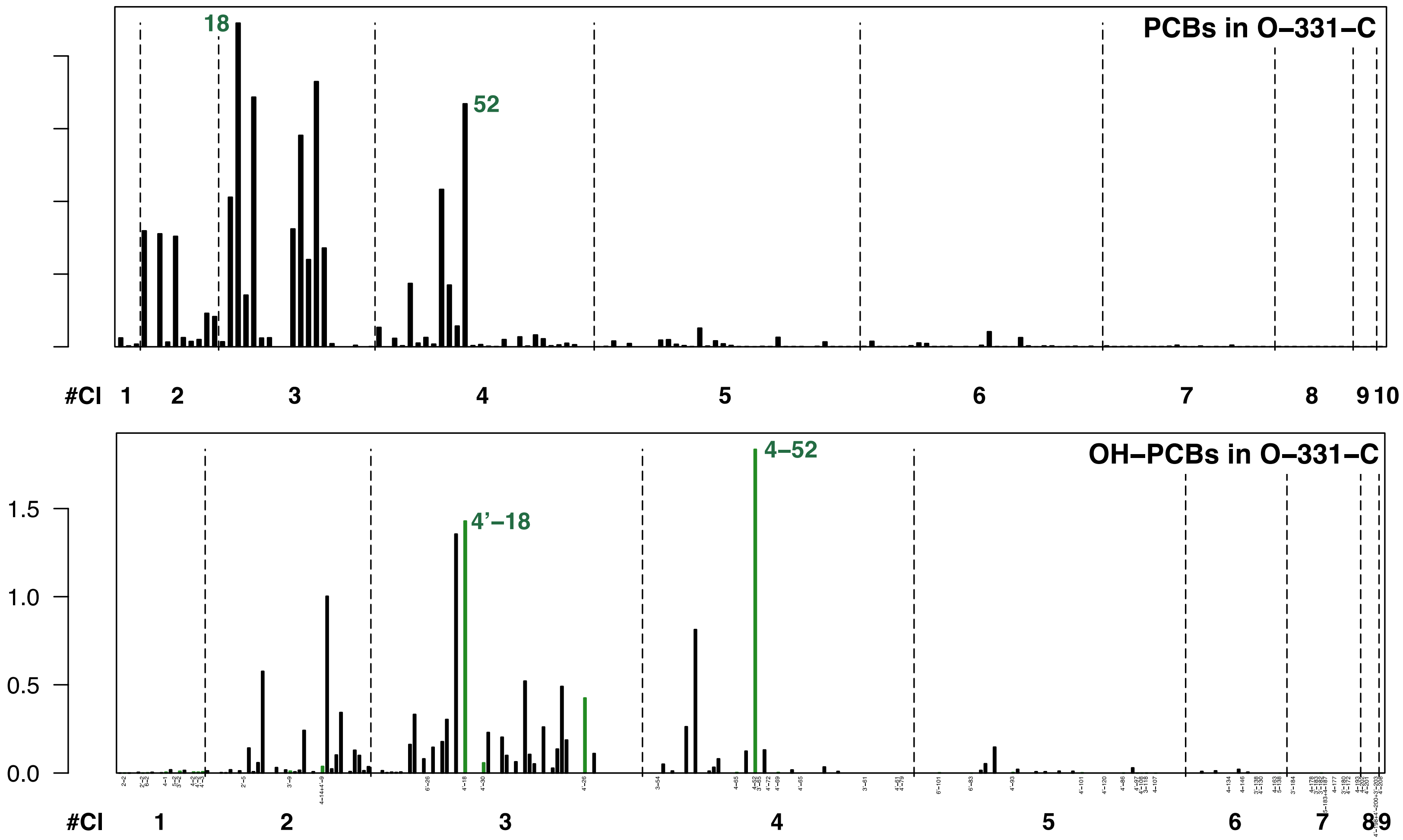

Figure S9. The congener profiles of PCB (above) and OH-PCBs (below) in O-331-C sediment from New Bedford Harbor (NBH) in µg/g dry weight.
OH-PCB congeners are arranged by homologs (#Cl) and peak elution order from Supelco SPB-Octyl capillary column with green bars indicating knowns and black bars indicating unknown.
Individual congener concentrations are available at Saktrakulkla et al. (2021).[13]

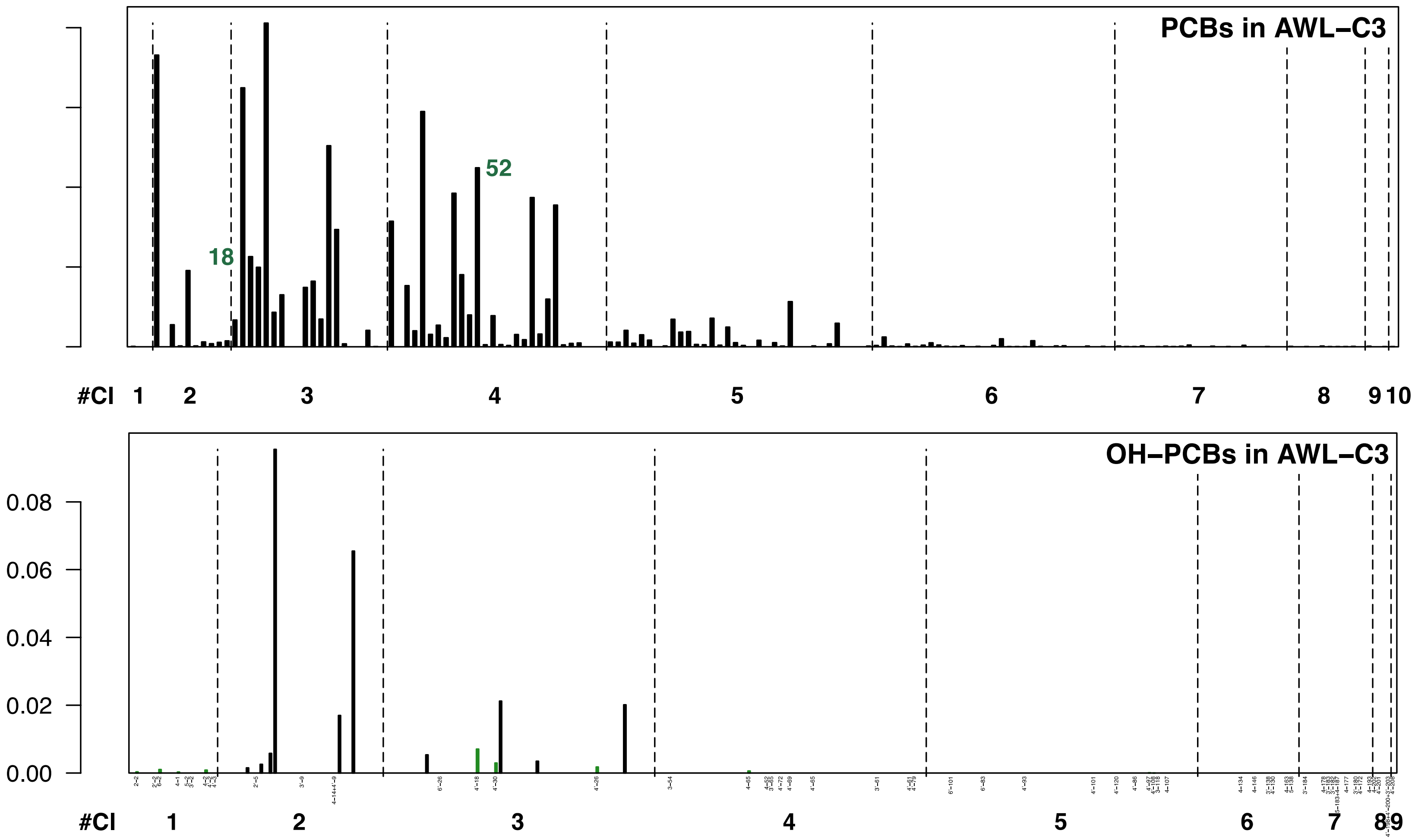

Figure S10. The congener profiles of PCB (above) and OH-PCBs (below) in C3 sediment from Altavista Waster Lagoon (AWL) in μg/g dry weight.
OH-PCB congeners are arranged by homologs (#Cl) and peak elution order from Supelco SPB-Octyl capillary column with green bars indicating knowns and black bars indicating unknown.
Individual congener concentrations are available at Saktrakulkla et al. (2021).[13]

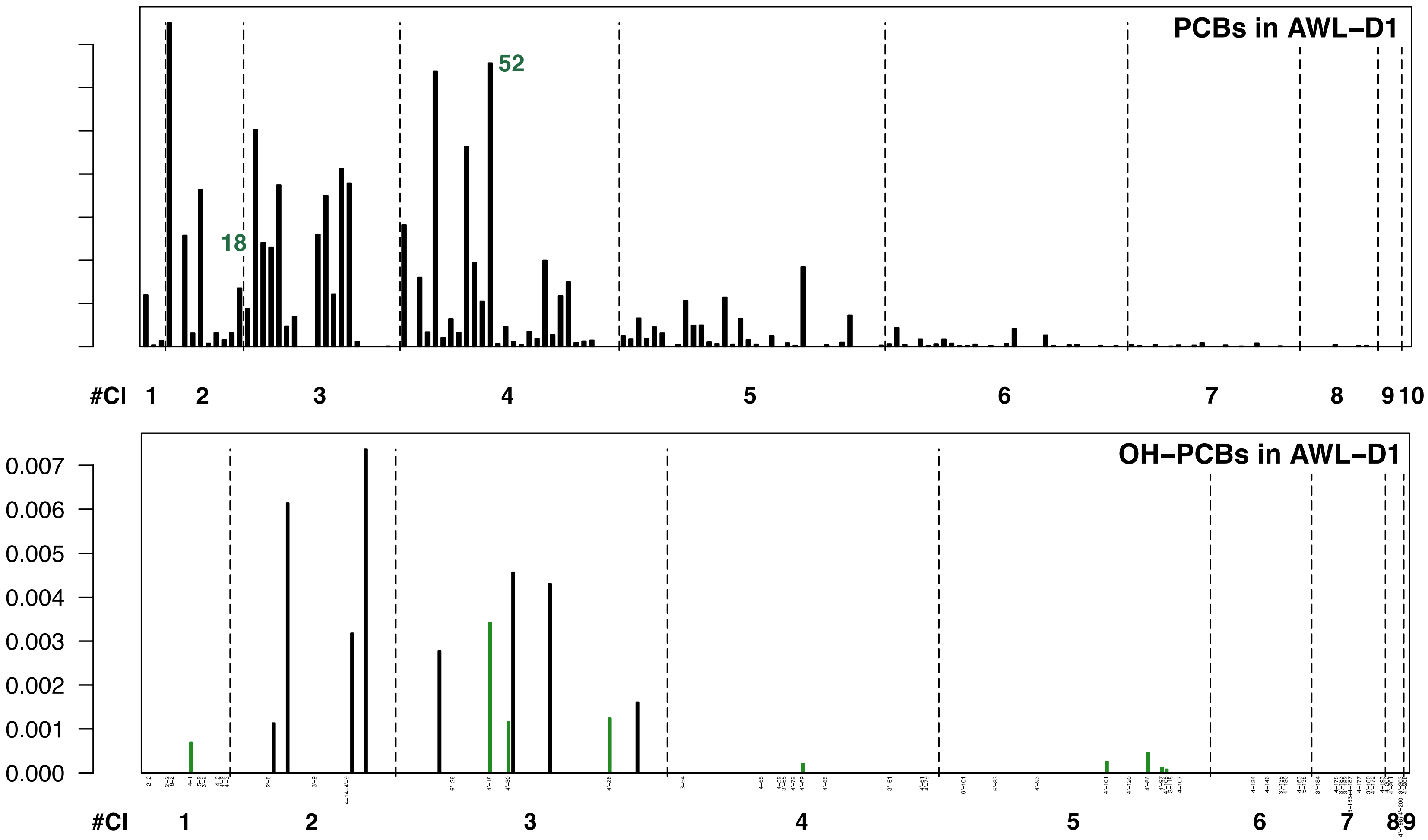

Figure S11. The congener profiles of PCB (above) and OH-PCBs (below) in D1 sediment from Altavista Waster Lagoon (AWL) in µg/g dry weight.
OH-PCB congeners are arranged by homologs (#Cl) and peak elution order from Supelco SPB-Octyl capillary column with green bars indicating knowns and black bars indicating unknown.
Individual congener concentrations are available at Saktrakulkla et al. (2021).[13]

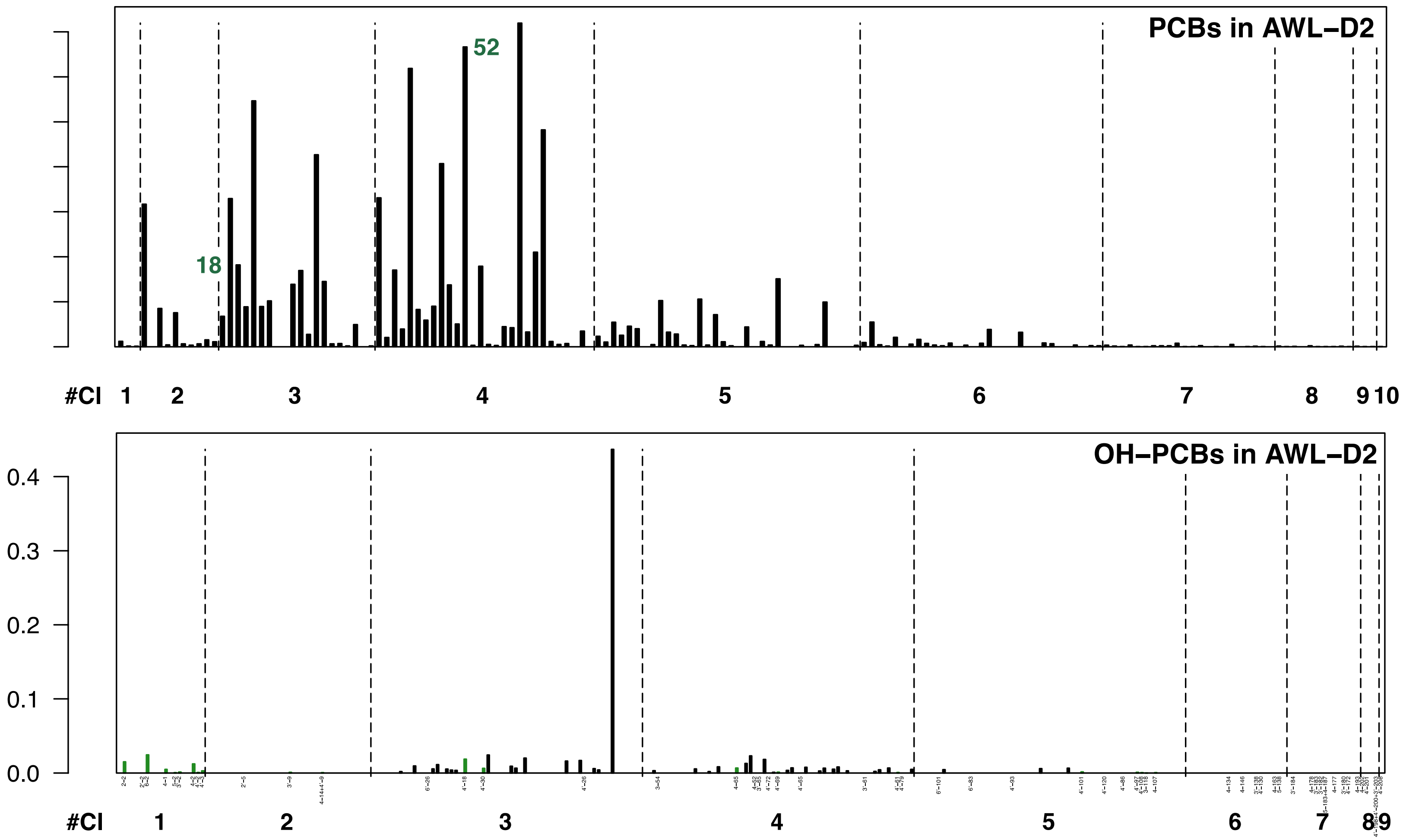

Figure S12. The congener profiles of PCB (above) and OH-PCBs (below) in D2 sediment from Altavista Waster Lagoon (AWL) in µg/g dry weight.
OH-PCB congeners are arranged by homologs (#Cl) and peak elution order from Supelco SPB-Octyl capillary column with green bars indicating knowns and black bars indicating unknown.
Individual congener concentrations are available at Saktrakulkla et al. (2021).[13]

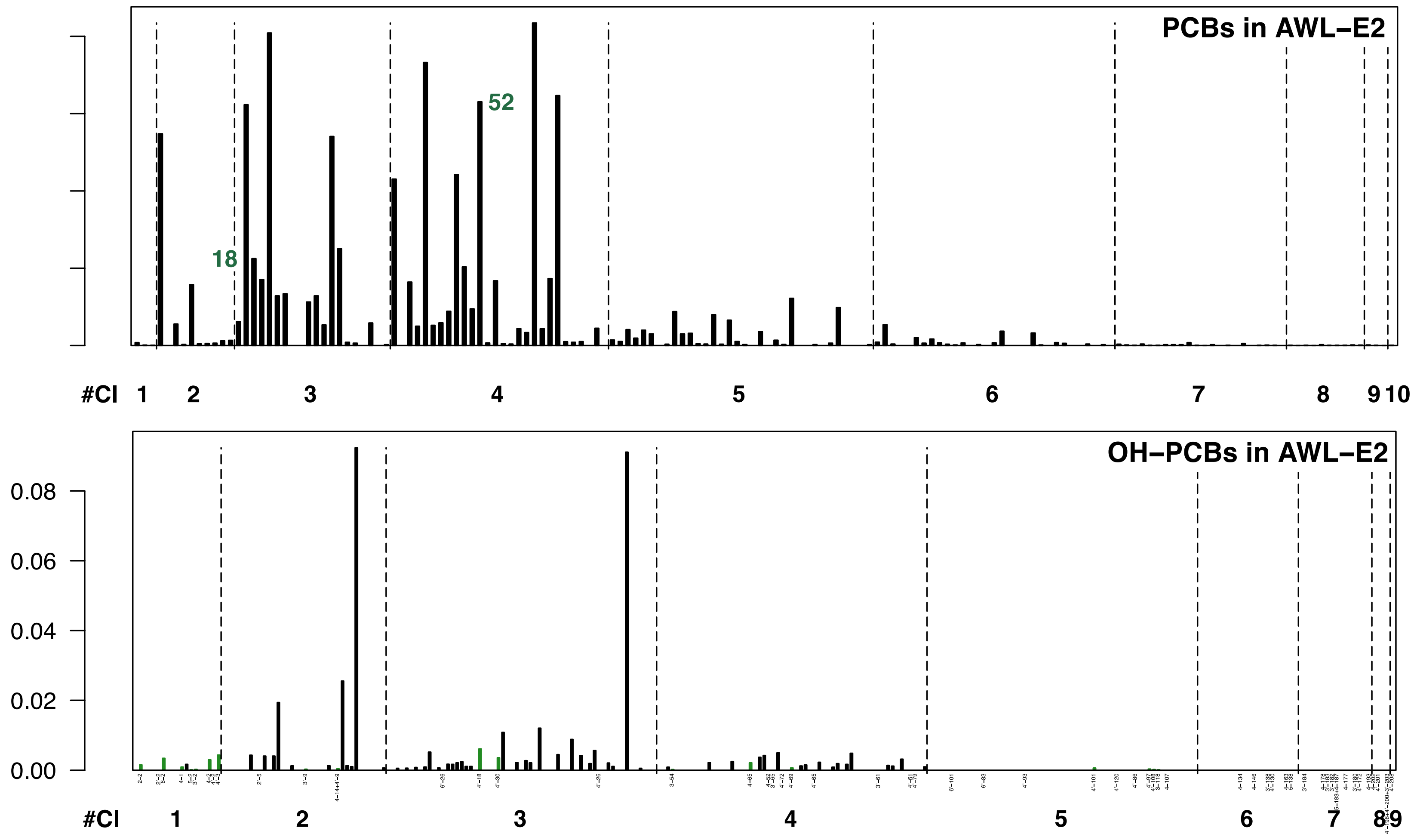

Figure S13. The congener profiles of PCB (above) and OH-PCBs (below) in E2 sediment from Altavista Waster Lagoon (AWL) in μg/g dry weight.
OH-PCB congeners are arranged by homologs (#Cl) and peak elution order from Supelco SPB-Octyl capillary column with green bars indicating knowns and black bars indicating unknown.
Individual congener concentrations are available at Saktrakulkla et al. (2021).[13]

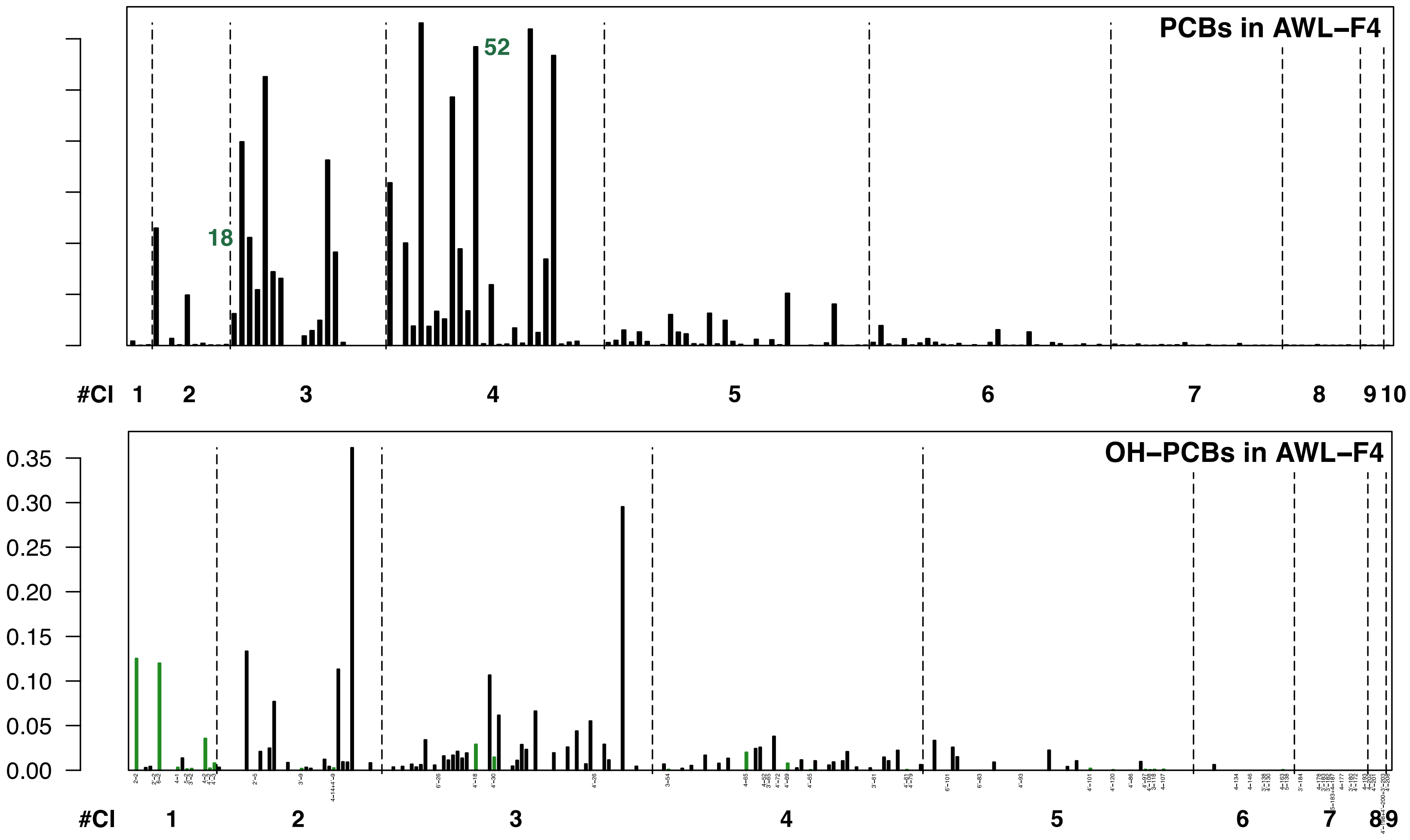

Figure S14. The congener profiles of PCB (above) and OH-PCBs (below) in F4 sediment from Altavista Waster Lagoon (AWL) in µg/g dry weight.
OH-PCB congeners are arranged by homologs (#Cl) and peak elution order from Supelco SPB-Octyl capillary column with green bars indicating knowns and black bars indicating unknown.
Individual congener concentrations are available at Saktrakulkla et al. (2021).[13]

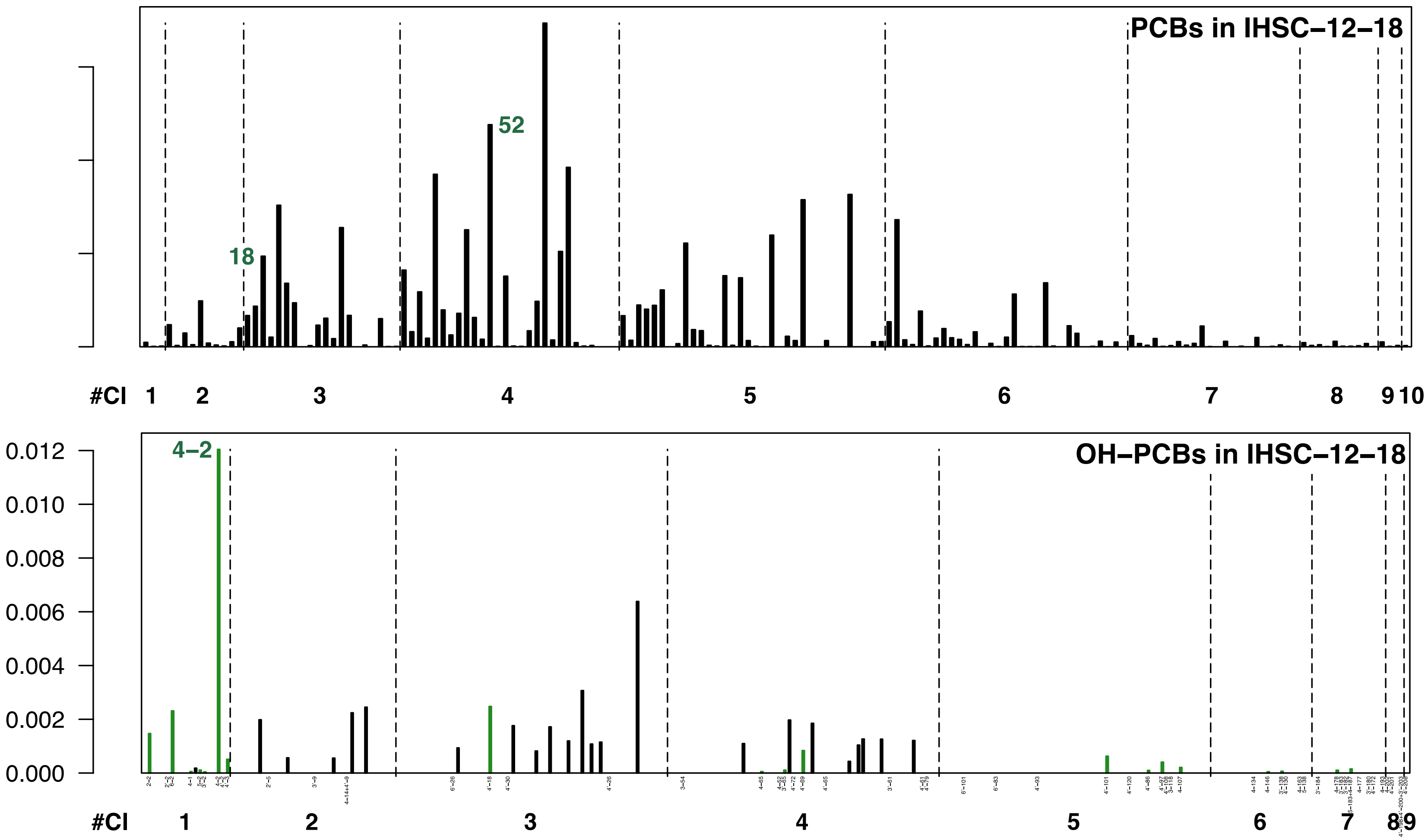

Figure S15. The congener profiles of PCB (above) and OH-PCBs (below) in 12-18" sediment from Indiana Harbor and Ship Canal (IHSC) in µg/g dry weight.
OH-PCB congeners are arranged by homologs (#Cl) and peak elution order from Supelco SPB-Octyl capillary column with green bars indicating knowns and black bars indicating unknown.
Individual congener concentrations are available at Saktrakulkla et al. (2021).[13]

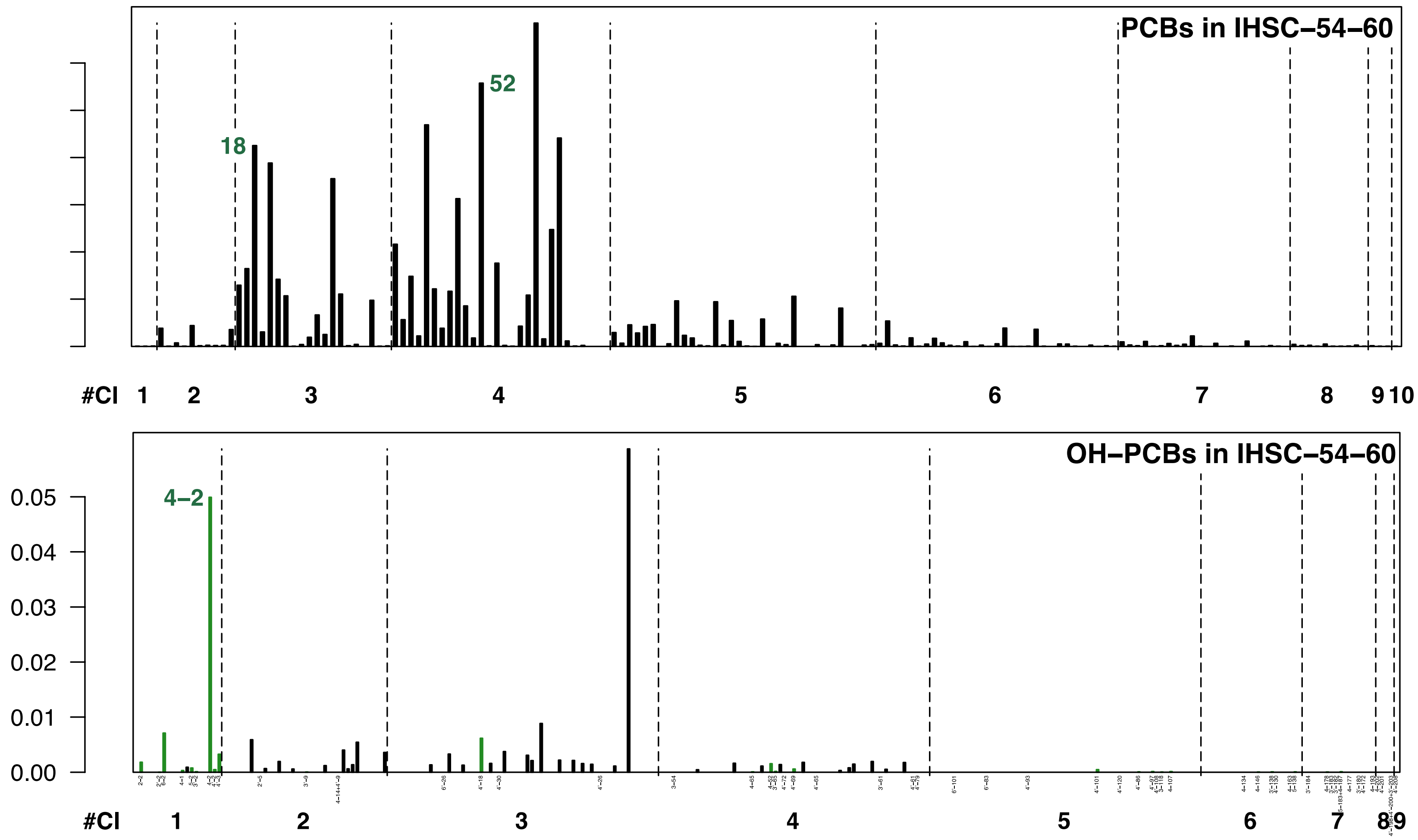

Figure S16. The congener profiles of PCB (above) and OH-PCBs (below) in 54-60" sediment from Indiana Harbor and Ship Canal (IHSC) in µg/g dry weight.
OH-PCB congeners are arranged by homologs (#Cl) and peak elution order from Supelco SPB-Octyl capillary column with green bars indicating knowns and black bars indicating unknown.
Individual congener concentrations are available at Saktrakulkla et al. (2021).[13]

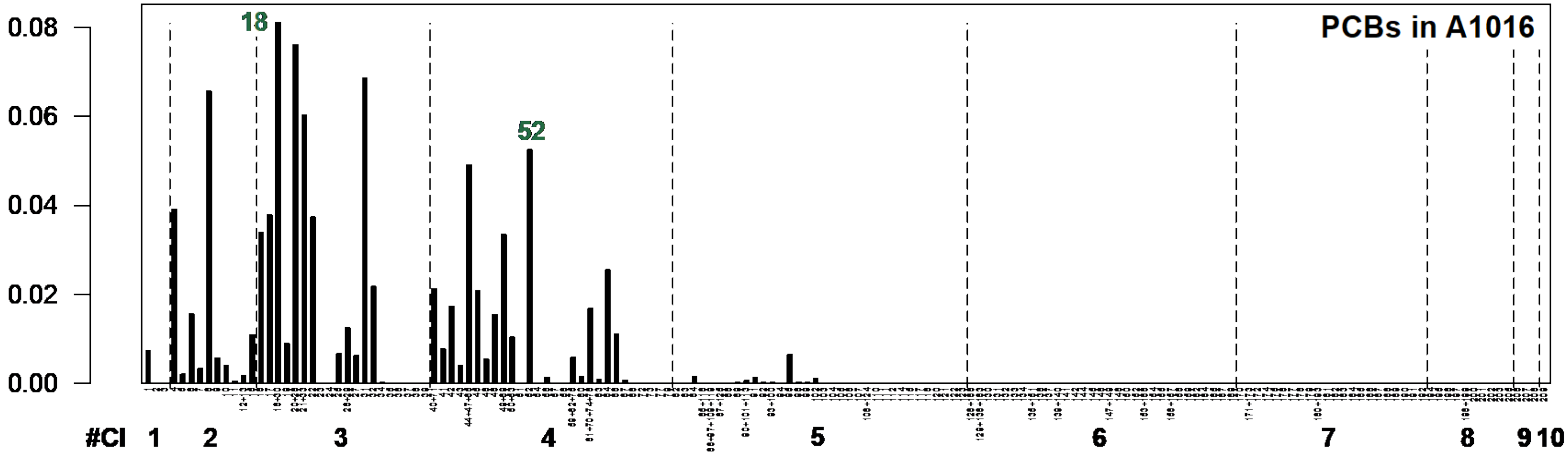

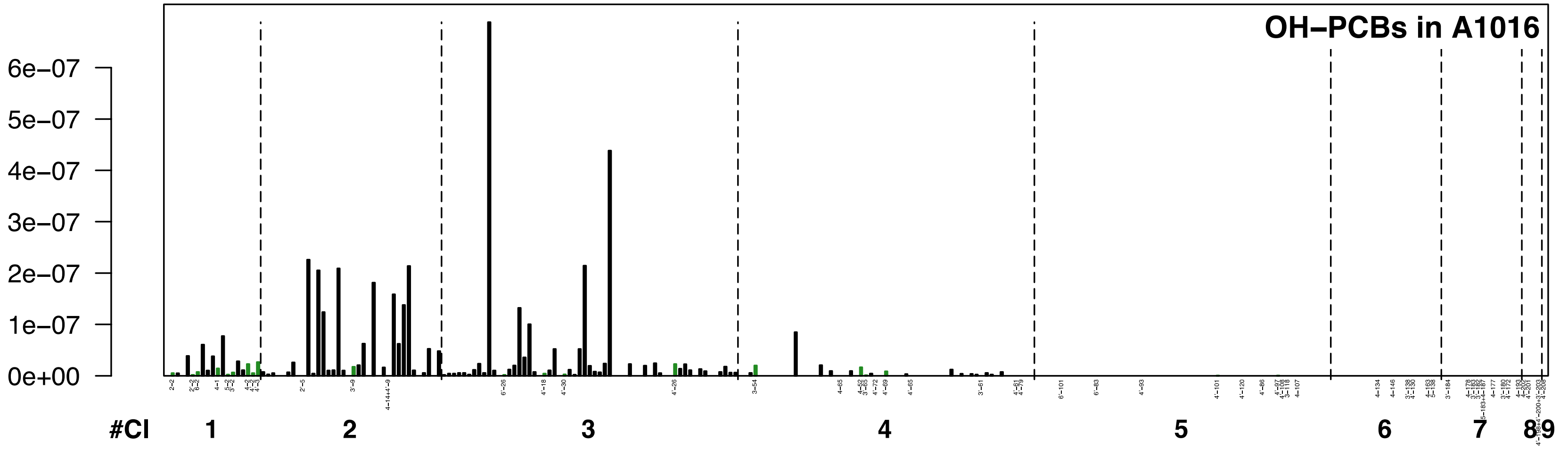

Figure S17. The congener profiles of PCB (above) and OH-PCBs (below) in Aroclor 1016 in µg/g dry weight. OH-PCB congeners are arranged by homologs (#Cl) and peak elution order from Supelco SPB-Octyl capillary column with green bars indicating knowns and black bars indicating unknown.

Individual congener concentrations are available at Saktrakulkla et al. (2021).[13]

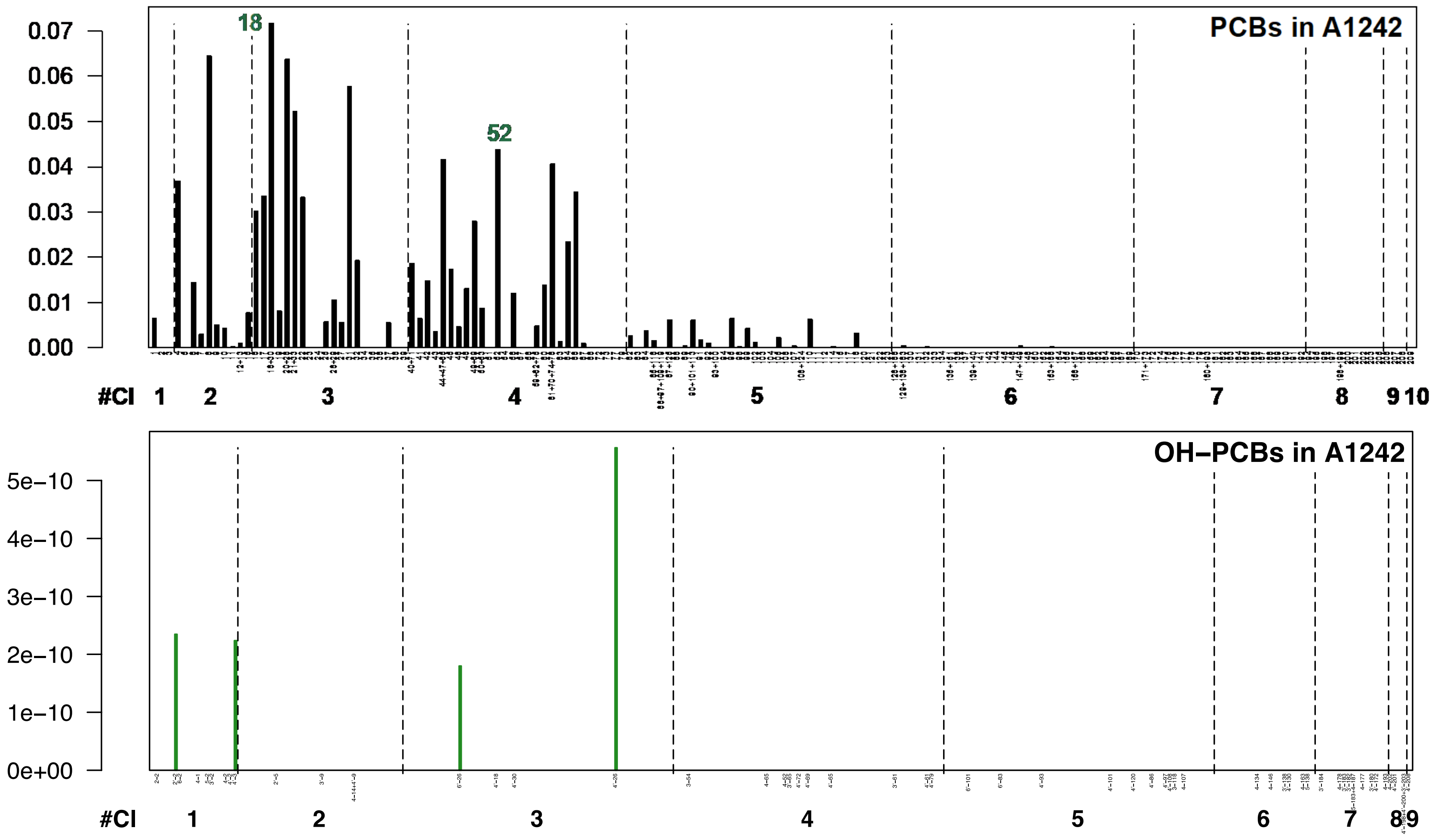

Figure S18. The congener profiles of PCB (above) and OH-PCBs (below) in Aroclor 1242 in µg/g dry weight. OH-PCB congeners are arranged by homologs (#Cl) and peak elution order from Supelco SPB-Octyl capillary column with green bars indicating knowns and black bars indicating unknown.

Individual congener concentrations are available at Saktrakulkla et al. (2021).[13]

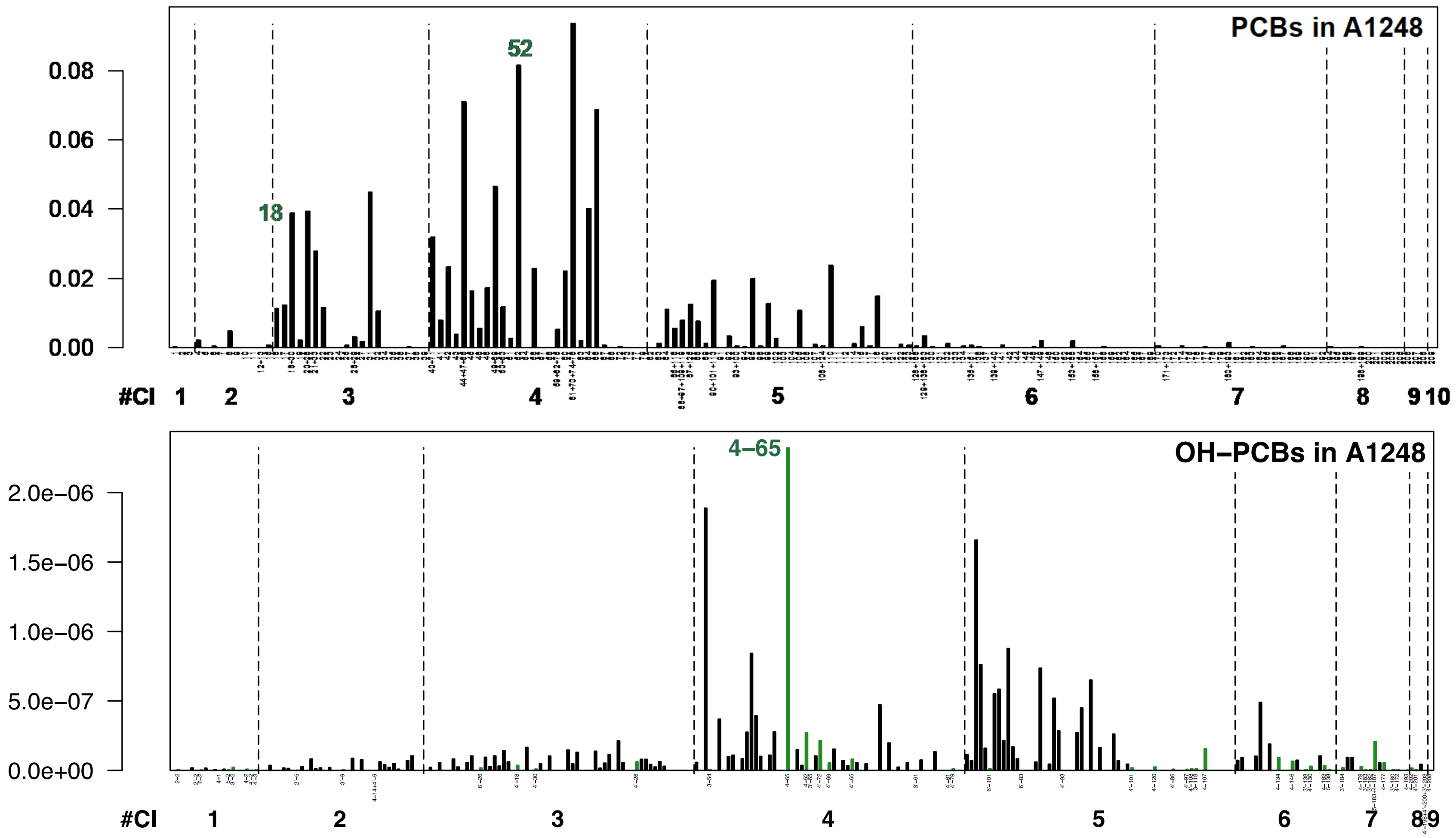

Figure S19. The congener profiles of PCB (above) and OH-PCBs (below) in Aroclor 1248 in µg/g dry weight. OH-PCB congeners are arranged by homologs (#Cl) and peak elution order from Supelco SPB-Octyl capillary column with green bars indicating knowns and black bars indicating unknown.
Individual congener concentrations are available at Saktrakulkla et al. (2021).[13]

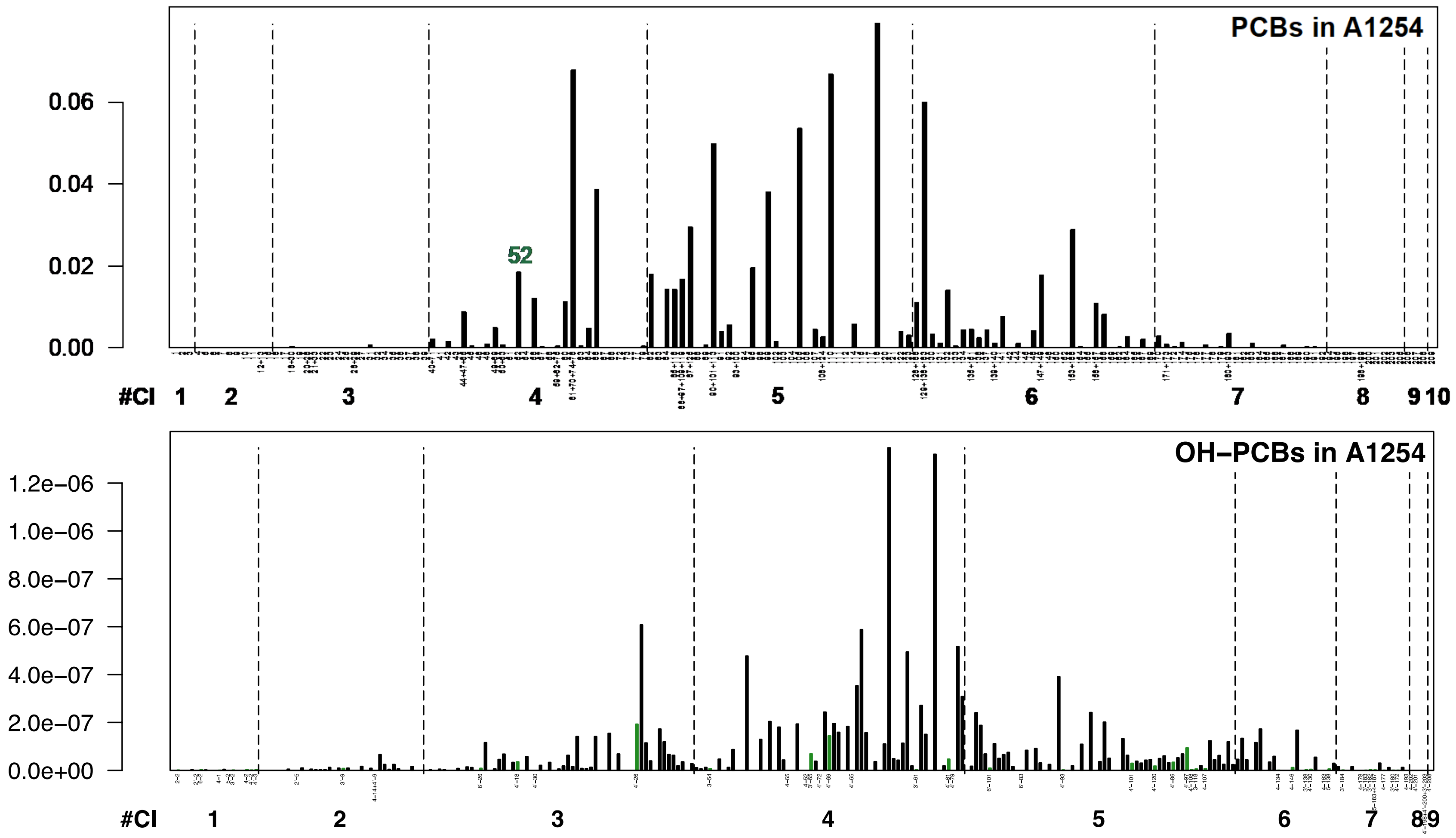

Figure S20. The congener profiles of PCB (above) and OH-PCBs (below) in Aroclor 1254 in µg/g dry weight. OH-PCB congeners are arranged by homologs (#Cl) and peak elution order from Supelco SPB-Octyl capillary column with green bars indicating knowns and black bars indicating unknown.
Individual congener concentrations are available at Saktrakulkla et al. (2021).[13]

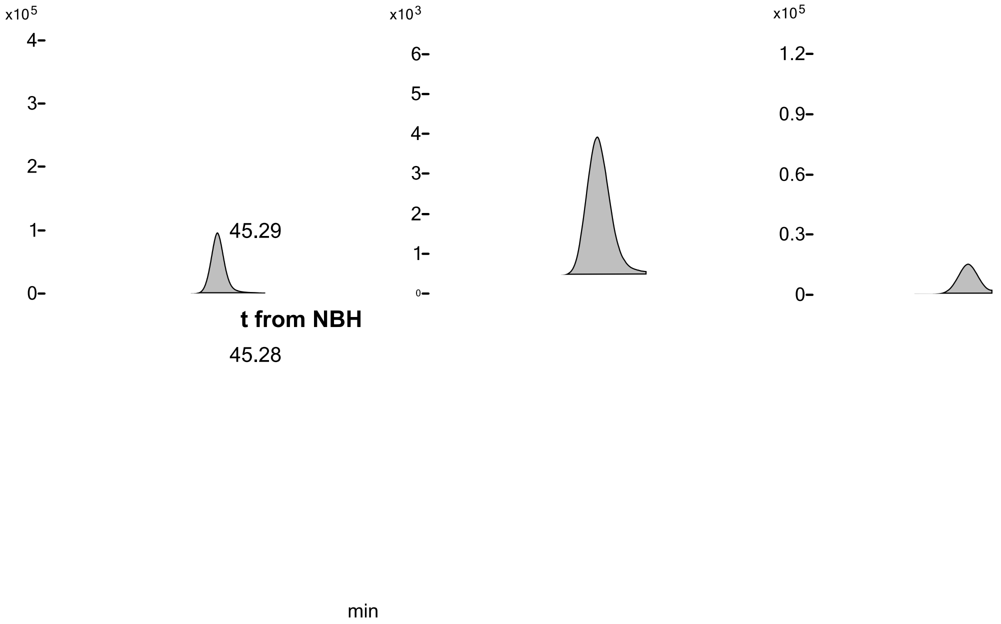

Figure S21. Chromatographic evidence of three major OH-PCB congeners found in sediments. The OH-PCB congeners derivatized to MeO-PCBs were captured with the QqQ mass spectrometer in positive EI in MRM mode.
Peaks highlighted in gray represent 4-OH-PCB52 (left panels, MRM transition: 321.9 → 278.9 m/z by CID at 20 eV), 4'-OH-PCB18 (middle panels, MRM transition 268.0 → 243.0 m/z by CID at 25 eV), and 4-OH-PCB2 (right panels, MRM transition 218.0 → 203.0 m/z by CID at 15 eV) found in authentic standards (top panels) and in sediments from NBH and IHSC (bottom panels). Unhighlighted peaks are other authentic standards (top panels) or known/unknown OH-PCBs (bottom panels).

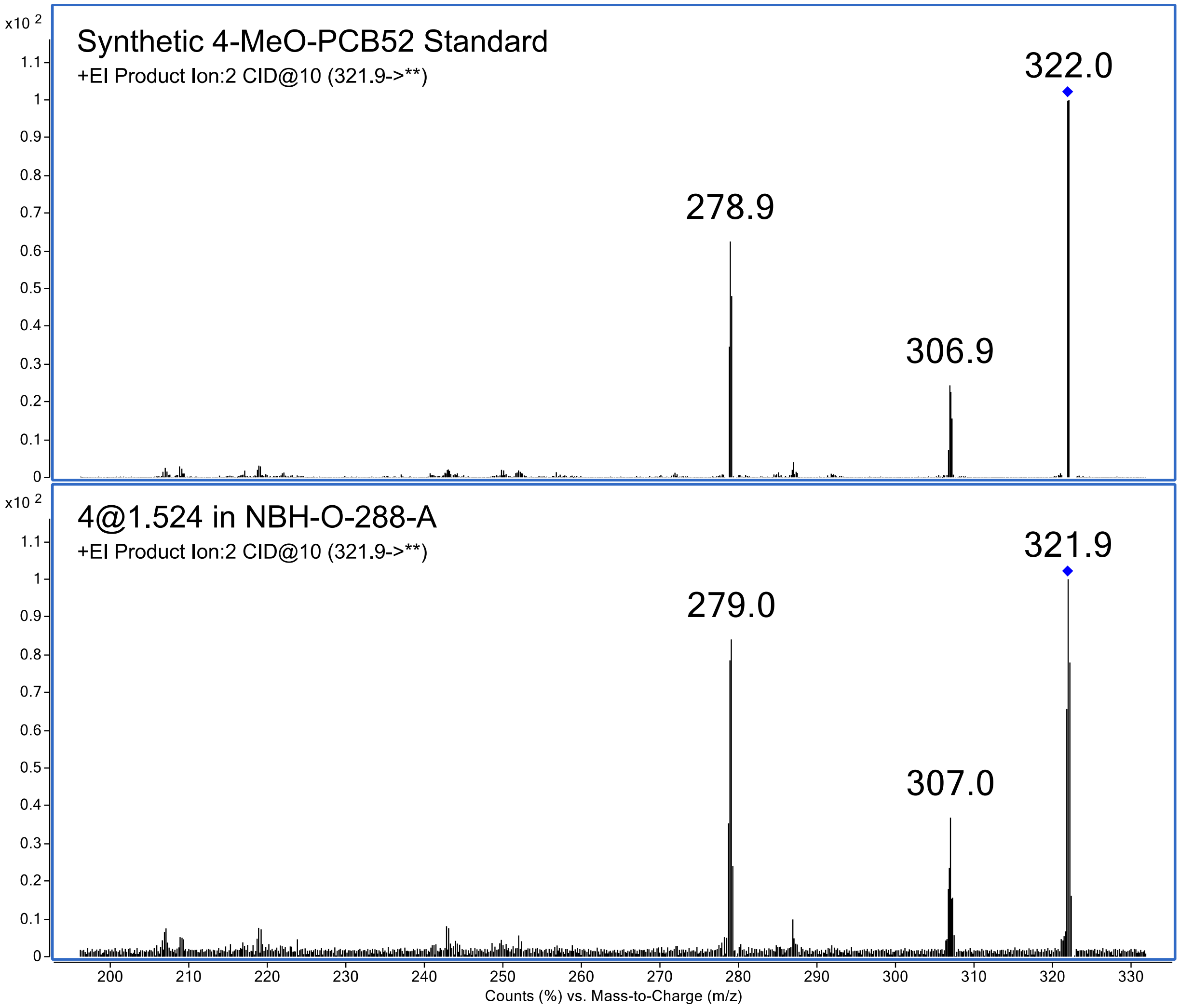

Figure S22. The product ion mass spectrum captured with CID energy of 10 eV from the 321.9-m/z parent ion of synthetic 4-MeO-PCB52 standard (above) and that of 4@1.524 in O-288-A sediments from NBH (below).

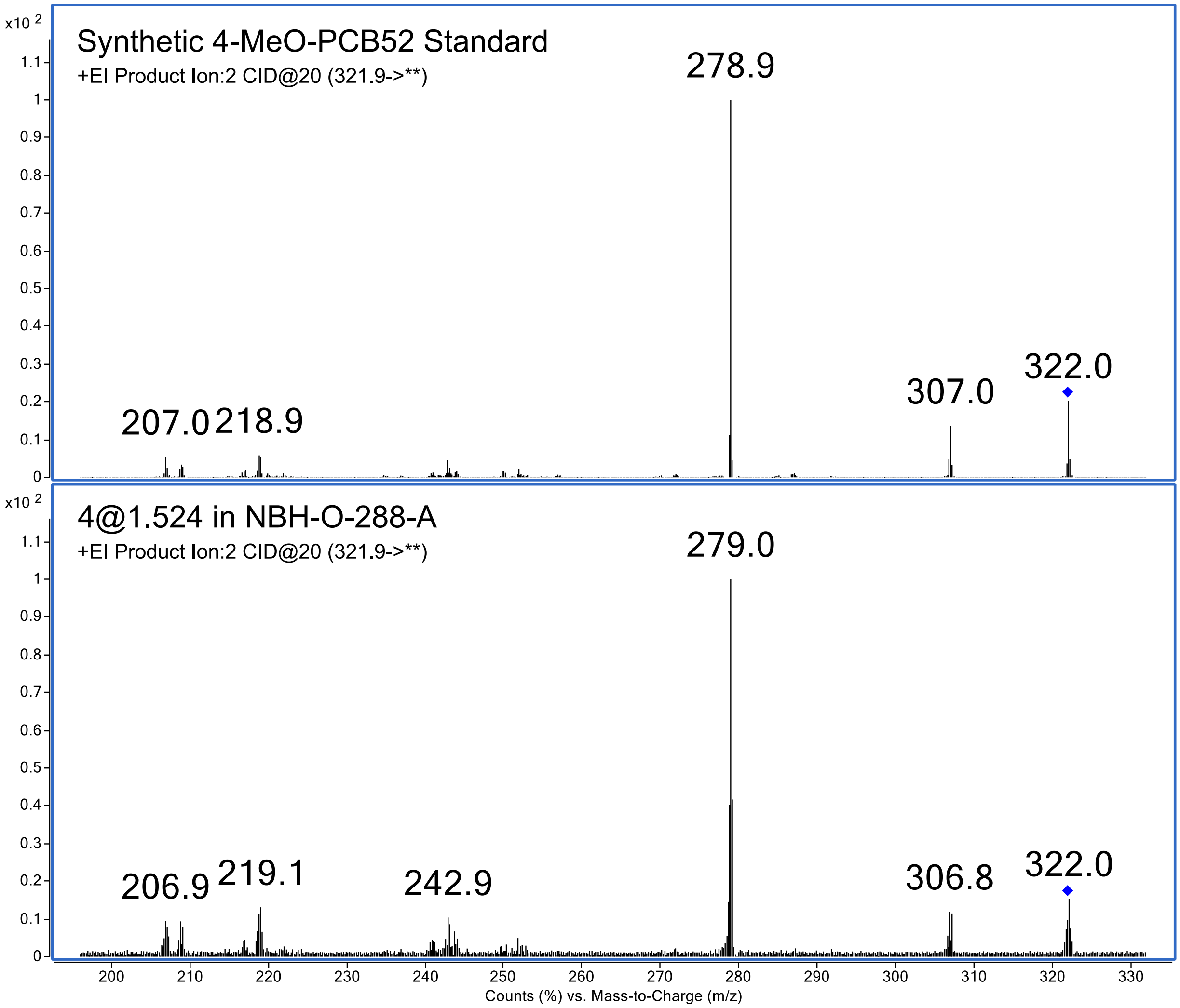

Figure S23. The product ion mass spectrum captured with CID energy of 20 eV from the 321.9-m/z parent ion of synthetic 4-MeO-PCB52 standard (above) and that of 4@1.524 in O-288-A sediments from NBH (below).

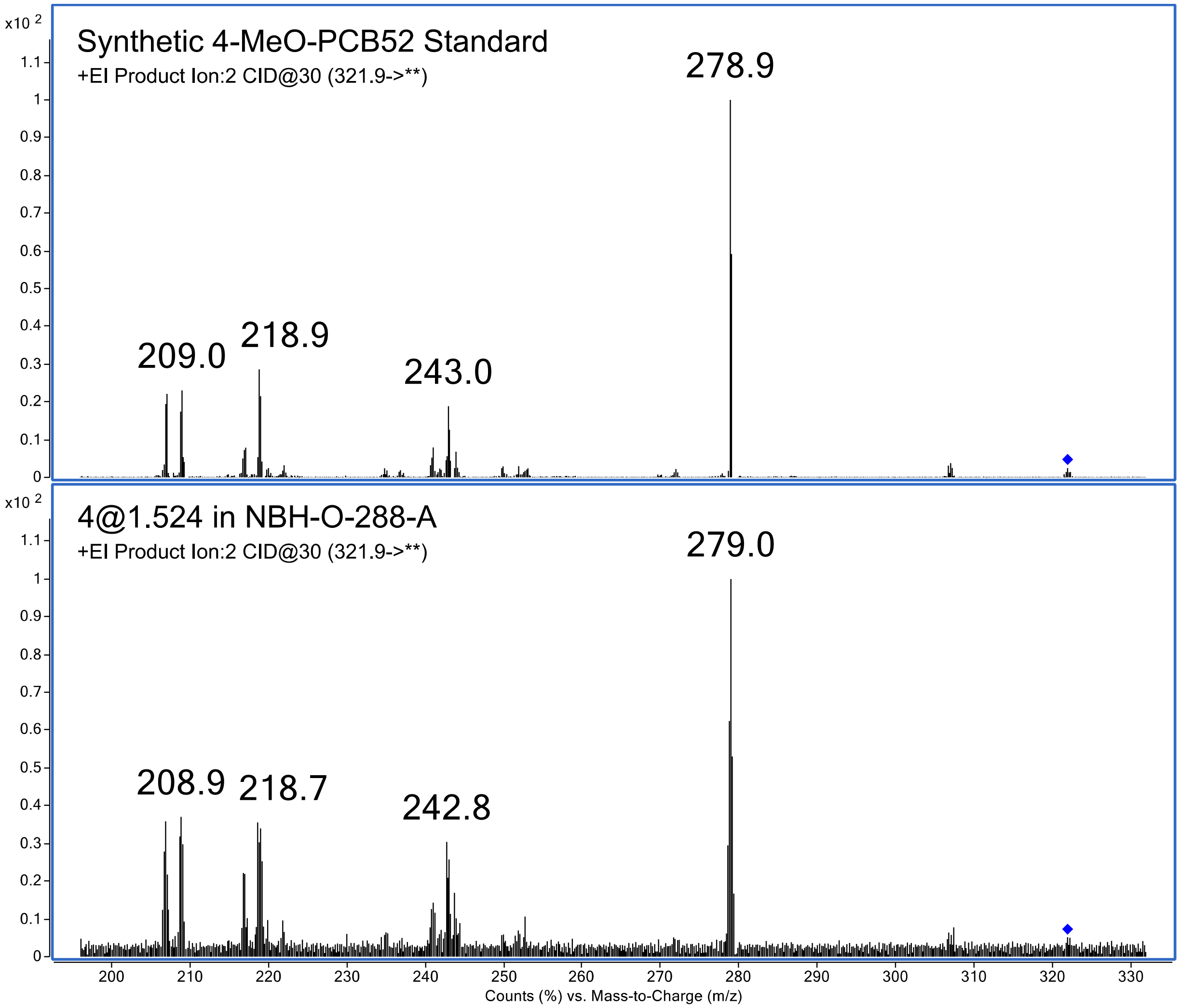

Figure S24. The product ion mass spectrum captured with CID energy of 30 eV from the 321.9-m/z parent ion of synthetic 4-MeO-PCB52 standard (above) and that of 4@1.524 in O-288-A sediments from NBH (below).

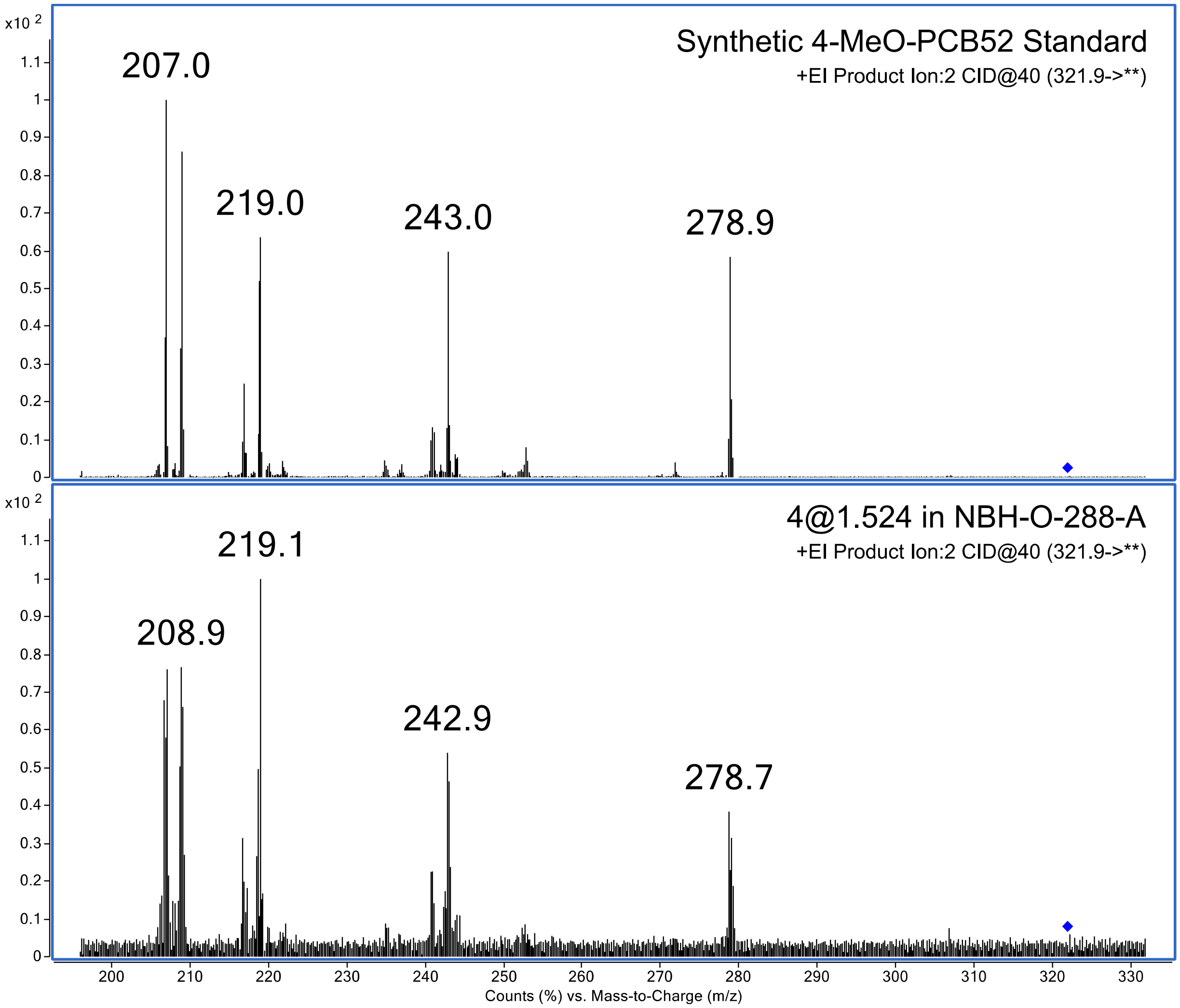

Figure S25. The product ion mass spectrum captured with CID energy of 40 eV from the 321.9-m/z parent ion of synthetic 4-MeO-PCB52 standard (above) and that of 4@1.524 in O-288-A sediments from NBH (below).

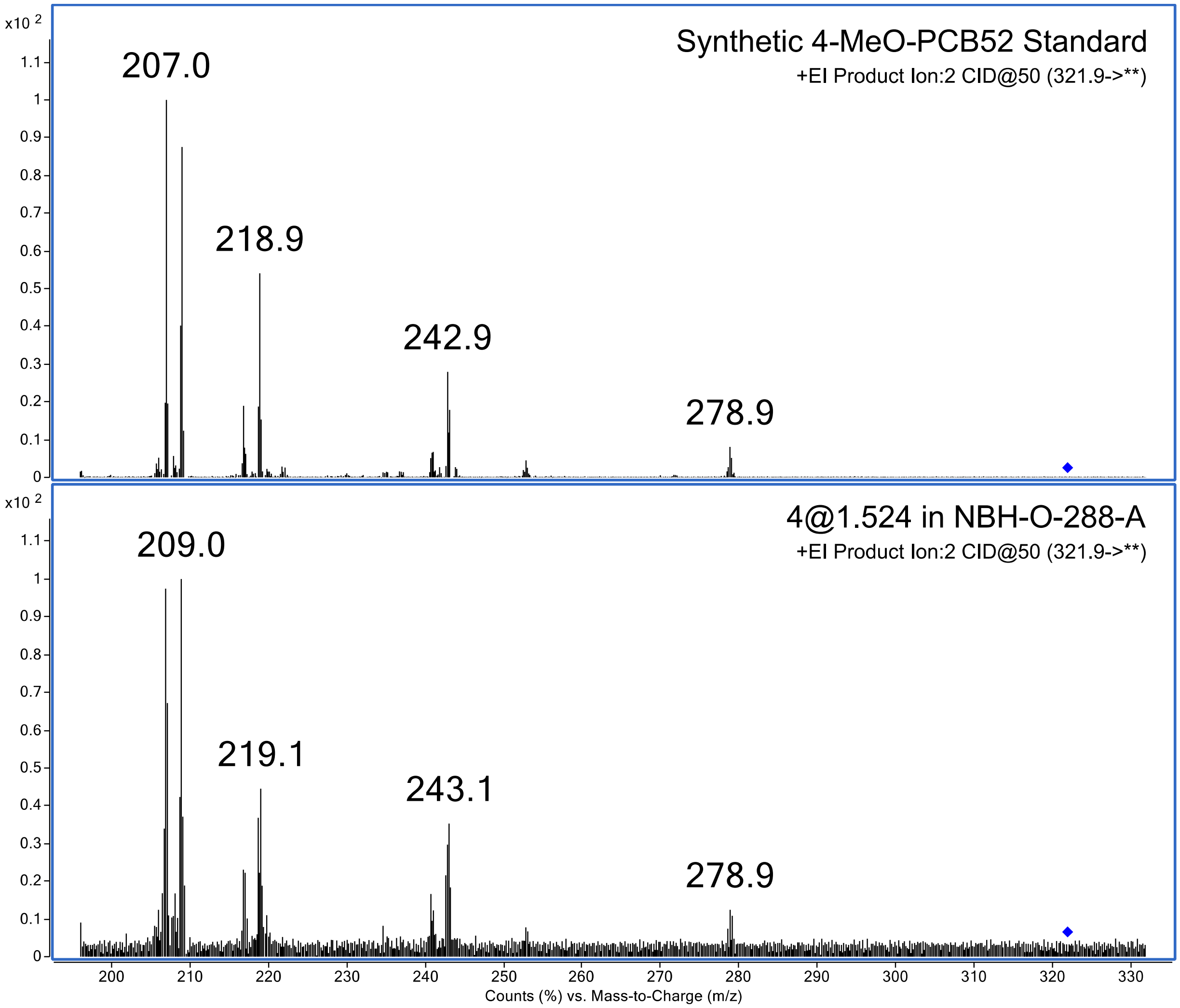

Figure S26. The product ion mass spectrum captured with CID energy of 40 eV from the 321.9-m/z parent ion of synthetic 4-MeO-PCB52 standard (above) and that of 4@1.524 in O-288-A sediments from NBH (below).

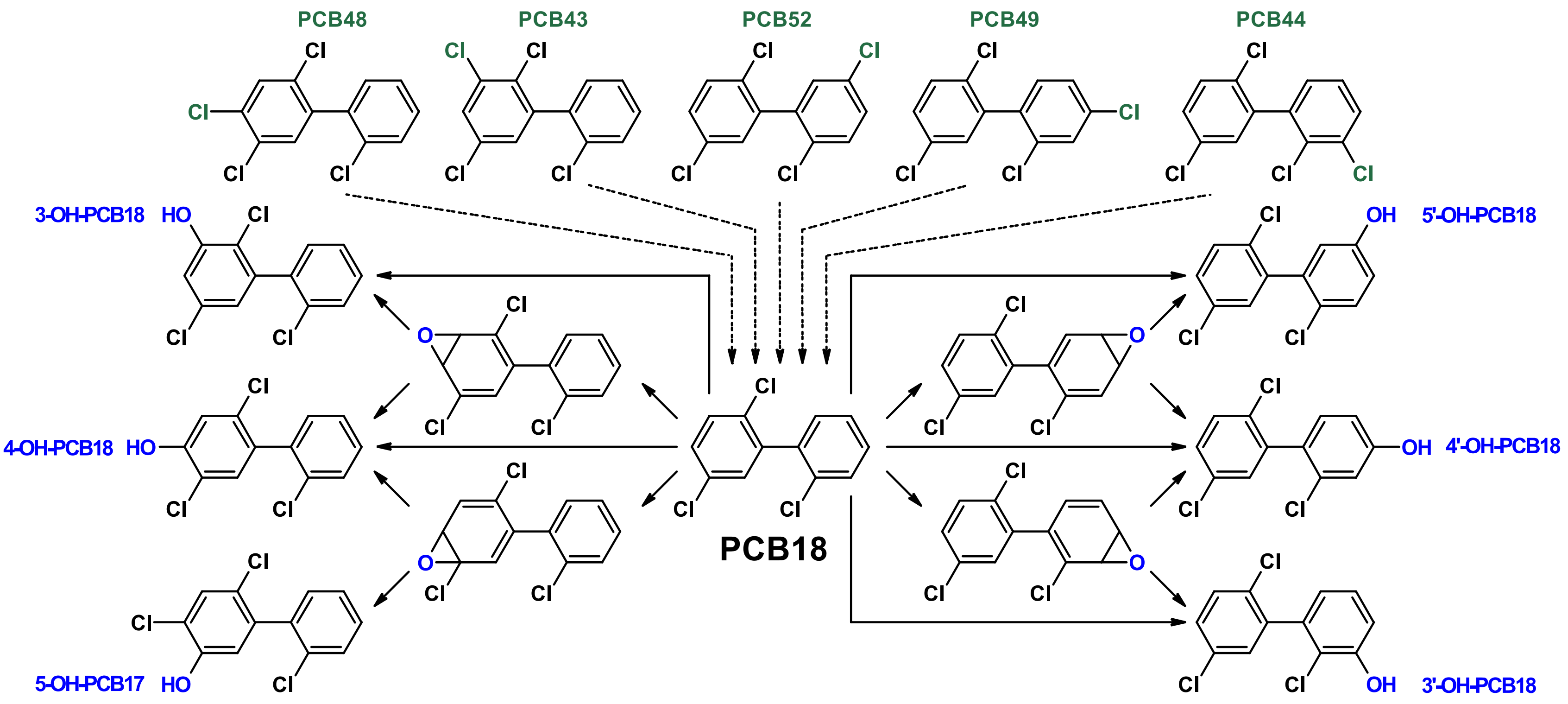

Figure S27. General hydroxylation through CYP450 of PCB18 (bold lines) and its potential dechlorination parent PCBs (dash lines).
The hydroxylation scheme is modified from Grimm et al. (2015),[14] and the dechlorination scheme is modified from Pieper and Seeger (2008).[15]

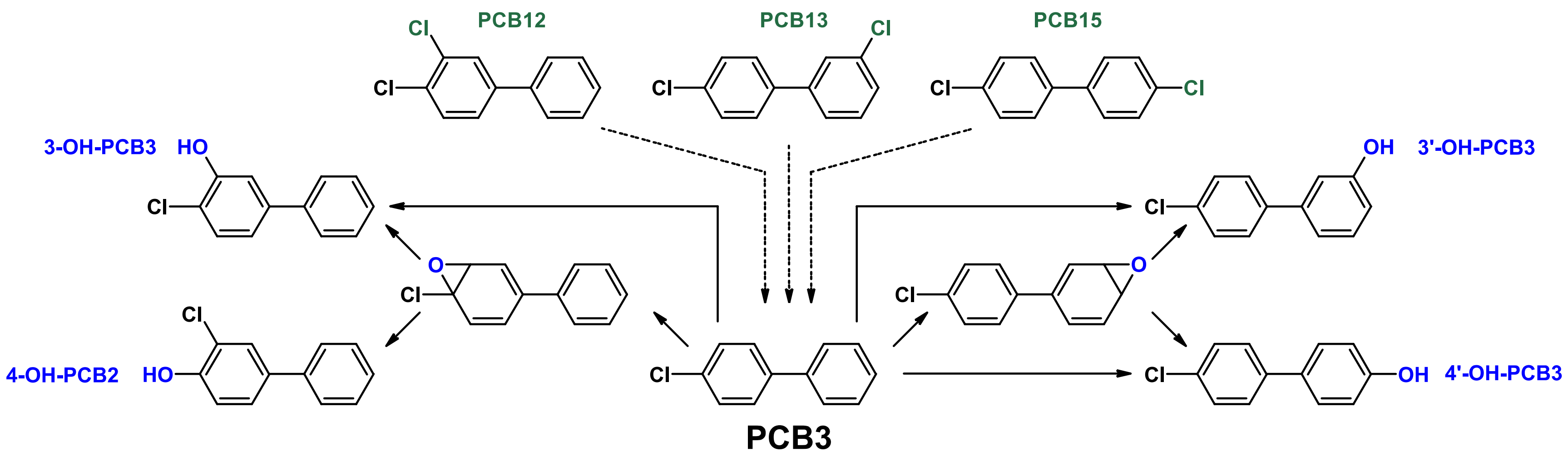

Figure S28. General hydroxylation through CYP450 of PCB3 (bold lines) and its potential dechlorination parent PCBs (dash lines).
The hydroxylation scheme is modified from Grimm et al. (2015),[14] and the dechlorination scheme is modified from Pieper and Seeger (2008).[15]

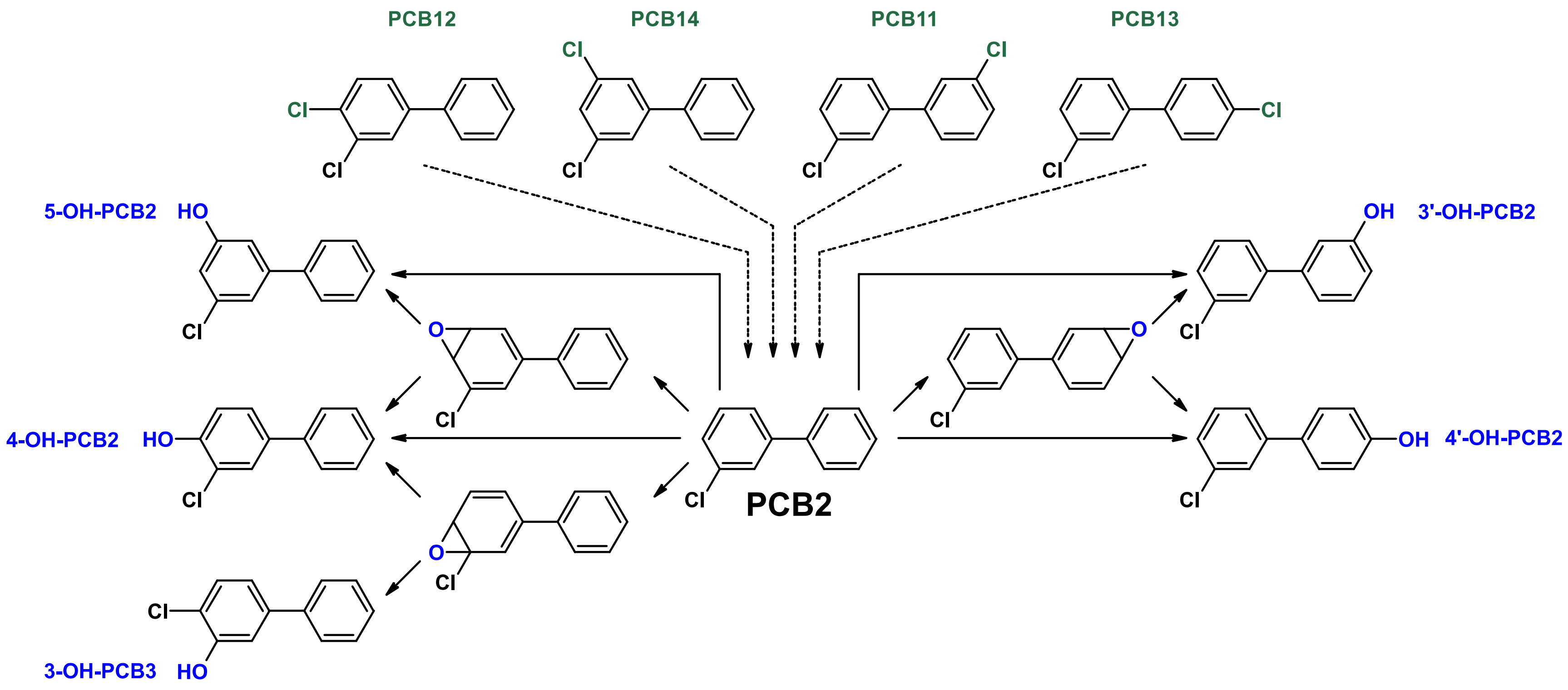

Figure S29. General hydroxylation through CYP450 of PCB2 (bold lines) and its potential dechlorination parent PCBs (dash lines).
The hydroxylation scheme is modified from Grimm et al. (2015),[14] and the dechlorination scheme is modified from Pieper and Seeger (2008).[15]

REFERENCES

1. United States Environmental Protection Agency (US EPA). Table of PCB Species by Congener Number. https://www.epa.gov/sites/production/files/2015-09/documents/congenertable.pdf (accessed 2021-04-12).
2. Patrick Curran. New Bedford Harbor Superfund Site: Final Dredge Areas I/N and O Hybrid Dredge Data Report. United States Environmental Protection Agency (US EPA),. https://semspub.epa.gov/src/document/01/100012483.pdf (accessed 2021-04-12).
3. Battelle Memorial Institute. Draft Final: Sediment Monitoring Summary Report 2014 Remedial Dredging Season. United States Environmental Protection Agency (US EPA),. https://www.epa.gov/sites/production/files/2015-09/documents/580304.pdf (accessed 2021-04-12).
4. Mattes, T. E.; Ewald, J. M.; Liang, Y.; Martinez, A.; Awad, A.; Richards, P.; Hornbuckle, K. C.; Schnoor, J. L., PCB dechlorination hotspots and reductive dehalogenase genes in sediments from a contaminated wastewater lagoon. *Environmental science and pollution research international* **2018,** *25*, (17), 16376-16388. DOI: https://doi.org/10.1007/s11356-017-9872-x
5. Martinez, A.; Hornbuckle, K. C., Record of PCB congeners, sorbents and potential toxicity in core samples in Indiana Harbor and Ship Canal. *Chemosphere* **2011,** *85*, (3), 542-547. DOI: https://doi.org/10.1016/j.chemosphere.2011.08.018
6. Saktrakulkla, P.; Lan, T.; Hua, J.; Marek, R. F.; Thorne, P. S.; Hornbuckle, K. C., Polychlorinated Biphenyls in Food. *Environmental Science & Technology* **2020,** *54*, (18), 11443-11452. DOI: https://doi.org/10.1021/acs.est.0c03632
7. Gross, J. H., *Mass Spectrometry: A Textbook*. 3nd ed.; Springer-Verlag: Heidelberg, DE, 2017. ISBN: 978-3-319-54398-7 DOI: https://doi.org/10.1007/978-3-319-54398-7
8. Ma, X.; Li, P.; Ma, T.-X.; Chu, G.-W.; Luo, Y.; Zou, H.-K.; Sun, B.; Chen, J.-F., Study on phenol sulfonation by concentrated sulfuric acid: Kinetics and process optimization. *Chemical Engineering Science* **2019,** *202*, 15-25. DOI: https://doi.org/10.1016/j.ces.2019.03.002
9. Meyer, A. H.; Paetz, D. H., Manufactural Geography of East Chicago-Whiting, Indiana: A Study in Geographic Rehabilitation. *Proceedings of the Indiana Academy of Science* **1961,** *71*, 171. URL: https://journals.iupui.edu/index.php/ias/article/download/5969/5984
10. The Encyclopedia of Chicago. East Chicago, IN. http://www.encyclopedia.chicagohistory.org/pages/402.html (accessed 2021-04-26).
11. CH2MHILL. Current Conditions Report for the DuPont East Chicago Facility Volume 1. https://semspub.epa.gov/src/document/05/1003356.pdf (accessed 2021-04-26).
12. Saktrakulkla, P.; Dhakal, R. C.; Lehmler, H. J.; Hornbuckle, K. C., A semi-target analytical method for quantification of OH-PCBs in environmental samples. *Environmental science and pollution research international* **2019**. DOI: https://doi.org/10.1007/s11356-019-05775-x
13. Saktrakulkla, P.; Martinez, A.; Lehmler, H.-J.; Hornbuckle, K. C., Dataset for OH-PCBs are emerging legacy pollutants in contaminated sediments. *University of Iowa* **2021** DOI: https://doi.org/10.25820/data.006146

14. Grimm, F. A.; Hu, D.; Kania-Korwel, I.; Lehmler, H. J.; Ludewig, G.; Hornbuckle, K. C.; Duffel, M. W.; Bergman, A.; Robertson, L. W., Metabolism and metabolites of polychlorinated biphenyls (PCBs). *Critical reviews in toxicology* **2015,** *45*, (3), 245-272. DOI: https://doi.org/10.3109/10408444.2014.999365
15. Pieper, D. H.; Seeger, M., Bacterial Metabolism of Polychlorinated Biphenyls. *Microbial Physiology* **2008,** *15*, (2-3), 121-138. DOI: https://doi.org/10.1159/000121325
